# Changes in the burden and underlying causes of rheumatic heart disease in children and youths, 1990–2021: an analysis of the Global Burden of Disease Study 2021

**DOI:** 10.3389/fcvm.2025.1597855

**Published:** 2025-06-26

**Authors:** Chang Li, Liuming Gao, Yanggan Wang

**Affiliations:** ^1^ Department of Geriatrics, General Medical Center, Zhongnan Hospital of Wuhan University, Wuhan, China; ^2^ Medical Research Institute of Wuhan University, Wuhan, China; ^3^ Frontier Science Center for Immunology and Metabolism, Wuhan University, Wuhan, China

**Keywords:** rheumatic heart disease, children, youths, global, epidemiology

## Abstract

**Background:**

Understanding the temporal evolutions in the burden of rheumatic heart disease (RHD) in children and youths is vital for devising effective and targeted preventative measures. Our objective was to deliver an accurate and thorough assessment of the prevalence, incidence and deaths of RHD in children and youths aged 5–19 years at global, regional, and national scales.

**Methods:**

We utilized the analytical tools provided by the Global Burden of Disease and Injuries (GBD) 2021 to assess the impact of RHD on the population of children and youths aged 5–19 years. This analysis considered factors such as sex, age, region, and encompassed 204 countries and territories spanning the years 1990–2021.

**Results:**

The global age-standardized incidence rate (ASIR, per 100,000 population) of RHD in children and youths notably increased from 77.98 (95% confidence interval: 51.93, 110.15) in 1990 to 93.96 (62.05, 134) in 2021. Similarly, the age-standardized prevalence rate also significantly increased from 498.49 (340.79, 686.31) to 588.46 (396.8, 816.79), with an estimated annual percentage change (EAPC) of 0.42% (0.4%, 0.44%). In contrast, the global age-standardized mortality rate (ASDR, per 100,000 population) declined moderately from 1.23 (1.020, 19.89) in 1990 to 0.52 (0.45, 0.58) in 2021, with an EAPC of −2.71% (−2.9%, −2.53%). When analyzed by sociodemographic index (SDI), regions with low and low-middle SDI exhibited a greater RHD burden compared to those with high and high-middle SDI. Geographically, Sub-Saharan Africa and South Asia experienced a higher prevalence of RHD than other regions. Additionally, gender disparities were observed: women exhibited a greater prevalence of RHD, while men demonstrated higher mortality rates associated with the condition. These trends highlight the persistent global burden of RHD, particularly in lower-resource settings and among specific demographic groups.

**Conclusions:**

The global burden of RHD among children and adolescents remained significant in 2021. The burden of RHD differs based on factors such as age, gender, SDI, region and country. RHD in children and youths is predominantly preventable, highlighting the need for increased focus on the targeted execution of efficient primary prevention strategies and the enhancement of healthcare systems that cater to young individuals.

## 1 Introduction

RHD, frequently overlooked by the media and policymakers, poses a significant challenge in developing nations, responsible for a large proportion of cardiovascular disease and death among the youth (1). According to data from the Global Burden Disease and Injuries (GBD) 2021, the total count of RHD cases rose markedly from 32.3 million to 54.8 million across the general population from 1990 to 2021 (2). Rheumatic heart disease is responsible for approximately 200,000–250,000 premature deaths annually (3) continues to be the primary contributor to cardiovascular-related deaths in children and adolescents within developing countries. During the late 20th century in high-income nations, the decrease in rheumatic heart disease cases was partly linked to better socioeconomic status and the extensive use of penicillin G benzathine for treating streptococcal pharyngitis (4). Despite these improvements, a high prevalence of RHD and related deaths persists in numerous areas, including Africa, South Asia, and the Pacific Islands (5–8). Consequently, there is a critical requirement to devise and execute efficient, targeted interventions focused on the primary prevention of RHD, particularly in low- and middle-income countries.

RHD develops as a result of heart valve impairment caused by an atypical immune reaction following a group A streptococcal infection, most commonly during childhood (9). Preventative strategies, primarily centered around the use of penicillin along with social and economic improvements, have proven to be highly effective and have almost eliminated rheumatic heart disease in developed nations. Nonetheless, the 2008 Population Reference Bureau (PRB) reported that approximately 80%–85% of children under 15 years old (roughly 2 billion) reside in regions where rheumatic heart disease remains prevalent (1). Globally, this condition stands as the primary factor leading to heart failure among children and youths, causing both disability and early mortality, while also significantly impacting the workforce in developing nations (3). Trends in demographics within these regions, such as restricted access to contraceptives and rural-to-urban migration are expected to result in a substantial increase in individuals at risk for RHD over the next decade (1).

Although previous studies have analyzed global RHD trends, few have focused specifically on the pediatric and adolescent population over a 30-year timespan. This study aims to fill this gap by providing a detailed age- and sex-stratified evaluation of global, regional, and national trends using GBD 2021 data (10).

## 2 Methods

### 2.1 Study participants

The Global Burden of Disease (GBD) 2021 offers a comprehensive and methodical analysis of publicly accessible, peer-reviewed, and contributed datasets, showcasing improved methodological methods and standardization concerning the prevalence, incidence, and deaths associated with 369 diseases and injuries across 204 countries and territories from 1990 to 2021, based on age, sex, and location. Details regarding the GBD 2021 had been previously documented (11). Our focus was on rheumatic heart disease (RHD) for individuals aged 5–19 years, specifically within the groups of 5–9 years, 10–14 years, and 15–19 years, consistent with earlier studies utilizing GBD data (12). We gathered information concerning the prevalence, incidence, and deaths related to RHD from the GBD 2021 database [Global Health Data Exchange. Global Burden of Disease (GBD) Study 2021. https://vizhub.healthdata.org/gbd-results]. The research relied on the data available from the GBD 2021 study and did not require ethical approval.

Estimation framework of the disease burden of RHD in children and youths in GBD 2021.

RHD and related fatalities were identified based on standardized definitions established in prior research (2). For the GBD 2021 study, the incidence and prevalence of RHD were estimated using DisMod-MR-2.1, a Bayesian meta-regression modeling tool. This approach integrated data from diverse sources, such as population surveys, cohort studies, registries, health system administrative records, and microdata from registry and cohort analyses (11, 13). Mortality data, classified according to International Classification of Diseases (ICD) codes, were extracted from vital registration systems and analyzed using the Cause of Death Ensemble model to determine RHD-related deaths rates (14). The research followed the Guidelines for Accurate and Transparent Health Estimates Reporting (GATHER) to ensure methodological rigor and transparency (11).

### 2.2 Statistical analysis

Drawing on data from the GBD 2021 analytical tools platform (accessible at https://vizhub.healthdata.org/gbd-results/), we performed a comprehensive evaluation to compute age-standardized prevalence, incidence, and mortality rates per 100,000 population, along with their 95% confidence intervals (CIs) for the years 1990 to 2021. This assessment utilized the world standard population as defined in GBD 2021, leveraging the “epitools” package in R version 4.1.0 (15). Furthermore, we calculated the EAPC and its 95% CI for these metrics over the same period, employing the Joinpoint Regression Program (version 4.9.0.0) developed by the U.S. National Cancer Institute to identify temporal trends (16). Statistical significance was assessed using a Monte Carlo permutation method (17). An increasing trend was inferred if EAPC > 0 and *P* < 0.05, while a decreasing trend was indicated if EAPC < 0 and *P* < 0.05; no significant change was concluded if *P* ≥ 0.05. Subgroup analyses were conducted across age groups (5–9, 10–14, and 15–19 years), sex (male and female), and World Bank regions (e.g., Sub-Saharan Africa, South Asia, North America, and others), categorized by the geometric mean of the mean years of schooling (14). Additionally, data were stratified into 21 GBD regions, including South Asia, East Asia, Western Sub-Saharan Africa, and others, as well as 204 countries and territories. The Pearson correlation between the SDI and age-standardized rates, along with EAPC, was examined. All statistical analyses were conducted using R version 4.1.0, with a two-sided *P* < 0.05 considered statistically significant.

### 2.3 Funding

The study's funding source had no role in the design, data collection, analysis, data interpretation, or manuscript preparation.

## 3 Results

### 3.1 Global burden of RHD by gender aged 5–19 years

Globally in 2021, the age-standardized incidence, prevalence, and mortality rate per 100,000 people of RHD aged 5–19 years was 93.96 (95% CI: 62.05, 134), 588.46 (396.8, 816.79), and 0.52 (0.45, 0.58), respectively (Table 1 and Supplementary file S1).

**Table 1:** Age-standardized incidence, prevalence and deaths rate of the RHD from 1990 to 2021 by SDI.

	Incidence				Prevalence				Death			
	Rate per 1,00,000 (95% CI) in 1990	Rate per 1,00,000 (95% CI) in 2021	Average annual percent change %, (95% CI)	Change (1990–2021)	Rate per 1,00,000 (95% CI) in 1990	Rate per 1,00,000 (95% CI) in 2021	Average annual percent change %, (95% CI)	Change (1990–2021)	Rate per 1,00,000 (95% CI) in 1990	Rate per 1,00,000 (95% CI) in 2,021	Average annual percent change %, (95% CI)	Change (1,990–2,021)
Rheumatic heart disease												
Global	77.98	93.96	0%	0.45%	498.49	588.46	0.96%	0.42%	1.23	0.52	−2.71%	0%
	(51.93–110.15)	(62.05–134)	(0–0)	(0.43–0.47)	(340.79–686.31)	(396.8–816.79)	(0.82–1.1)	(0.4–0.44)	(1.02–1.48)	(0.45–0.58)	(−2.9 to −2.52)	(−0.57 to −0.4)
SDI category												
High	1.67	1.8	0%	0.01%	18.11	18.94	0%	−0.01%	0.09	0.02	0%	−0.79%
	(1.19–2.23)	(1.27–2.4)	(0–0)	(−0.06 to 0.09)	(14.37–21.96)	(14.98–22.96)	(0–0)	(−0.07 to 0.05)	(0.08–0.1)	(0.02–0.02)	(0–0)	(−0.82 to −0.76)
High-middle	36.38	31.21	0%	−0.28%	272.67	227.58	0%	−0.3%	0.4	0.08	0%	−1%
	(24.2–51.55)	(20.55–44.07)	(0–0)	(−0.31 to −0.25)	(191.64–371.06)	(159.03–312.3)	(0–0)	(−0.32 to −0.28)	(0.36–0.47)	(0.07–0.09)	(0–0)	(−0.86 to −0.78)
Middle	90.65	85.74	0%	−0.04%	618.2	571.6	0%	−0.06%	1.03	0.33	0%	−0.68%
	(59.84–129.59)	(56.47–121.46)	(0–0)	(−0.06 to −0.02)	(423.83–853.82)	(388.97–789.88)	(0–0)	(−0.08 to −0.04)	(0.9–1.15)	(0.29–0.37)	(0–0)	(−0.74 to −0.61)
Low-middle	93.86	108.19	0%	0.58%	841.06	964.14	0%	1.54%	2.39	0.96	0%	−0.45%
	(62.59–132.09)	(71.81–155.84)	(0–0)	(0.54–0.63)	(565.52–1,169.56)	(644.67–1,345.34)	(0–0)	(1.5–1.6)	(1.9–2.98)	(0.83–1.1)	(0–0)	(−0.56 to −0.32)
Low	142.84	160.06	0%	1.48%	841.06	964.14	0%	2%	1.66	0.62	0%	−0.17%
	(94.07–201.77)	(104.66–229.87)	(0–0)	(1.43–1.54)	(565.52–1,169.56)	(644.67–1,345.34)	(0–0)	(1.5–1.6)	(1.23–2.17)	(0.51–0.75)	(0–0)	(−0.33–0.05)

CI, confidence interval.

**Figure 1:**
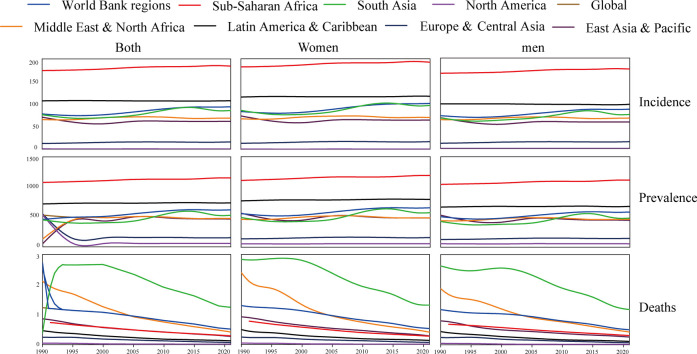
Temporal trends in age-standardized incidence, prevalence and mortality rate of rheumatic heart disease in youths overall and by sex (men and women) and World Bank regions from 1990 to 2021.

In 2021, a global analysis stratified by SDI revealed that countries classified as having low and low-middle SDI exhibited the highest age-standardized prevalence, incidence, and mortality rates associated with RHD. In contrast, nations with high SDI displayed the lowest burden of this disease (Table 1). This disparity highlights the significant impact of socio-economic factors on health outcomes, particularly concerning RHD. Further examination at regional and national levels indicated that territories within Sub-Saharan Africa recorded the highest age-standardized incidence and prevalence rates of RHD (Figure 1). Additionally, regions in South Asia presented the highest age-standardized mortality rates related to RHD (Figure 1 and Supplementary file S2).

### 3.2 Global burden of RHD by age in children and youths

**Figure 2:**
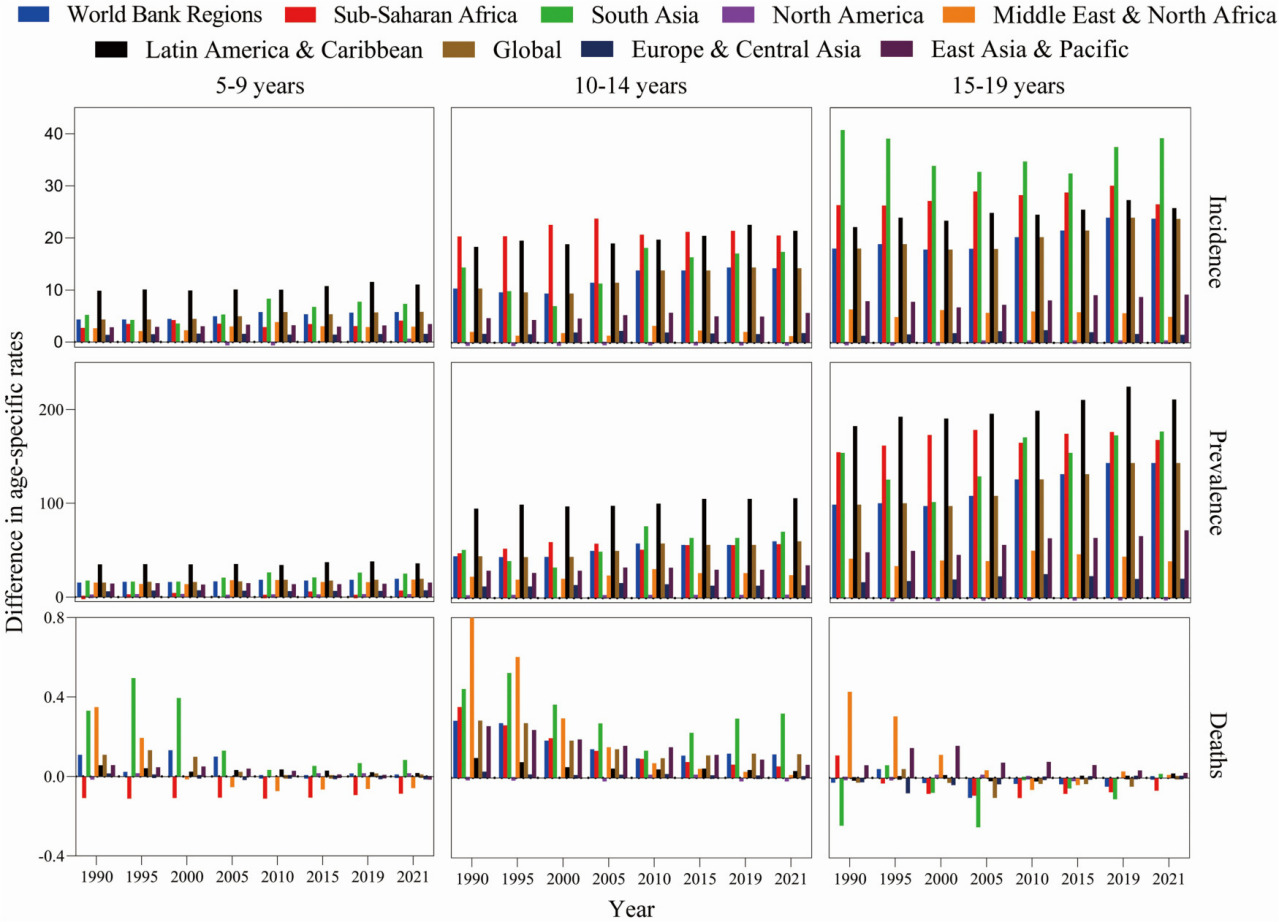
Difference in age-standardized incidence, prevalence and mortality rate of RHD in children and youths between men and women by age and World Bank regions from 1990 to 2021. The difference indicates the age-standardized rate in women minus that in men. A difference >0 suggests that women have a higher rate than men. EAPC, estimated annual percentage change.

In 2021, when examined globally, the age-standardized prevalence rate for women surpassed that of men, while the incidence and mortality rates were greater in men compared to women. When analyzed by age, sex, and World Bank regions, women exhibited a higher prevalence rate across all age groups and regional categories. Notably, women aged 15–19 years bracket displayed the highest incidence and prevalence rates, irrespective of the World Bank regions (Figure 2 and Supplementary file S3). Variations were observed in age-standardized incidence, prevalence, and mortality rates for these metrics across various World Bank regions, influenced by differences in age and sex (Figure 2 and Supplementary file S3). Incidence and prevalence of RHD in aged 5–9, 10–14, and 15–19 years children were significantly higher in women than in men, whereas women aged 15–19 years had a highest incidence and prevalence, regardless of world bank regions. Among the nine bank regions, the gender difference in South Asia was the most obvious. Over the last three decades, the incidence and prevalence of RHD have increased among both genders (Figure 4 and Supplementary file S3). Conversely, the death rates for both women and men have shown a significant reduction. Our findings indicate that the reduction in mortality rates has been more evident in women compared to men.

### 3.3 RHD burden in different countries

**Figure 3:**
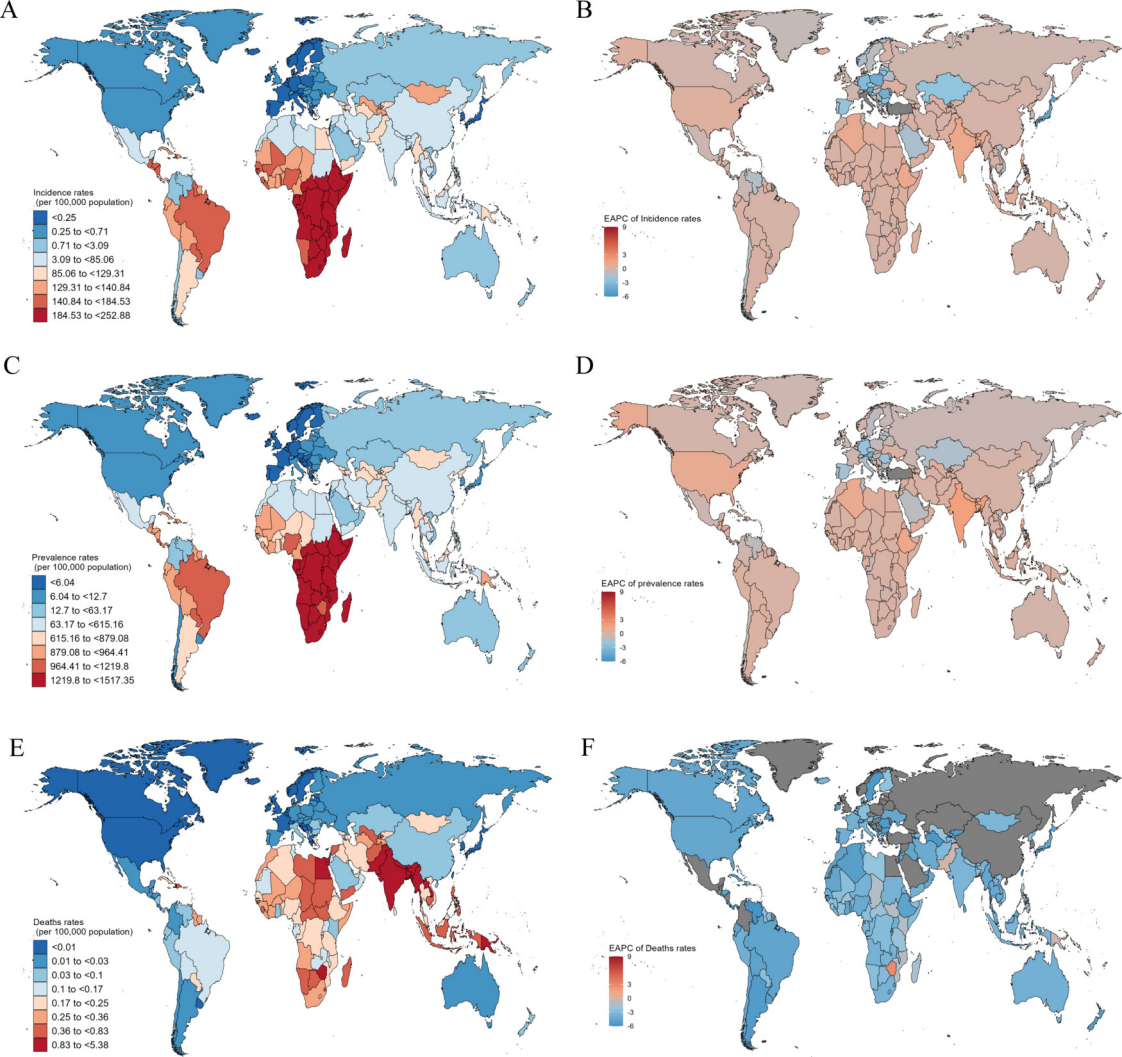
Incidence, prevalence, and death rates of children with RHD in different countries and territories in 2021. **(A)** Incidence rates; **(B)** the EAPC of RHD-related incidence between 1990 and 2021; **(C)** prevalence rates; **(D)** the EAPC of RHD-related prevalence between 1990 and 2021. **(E)** deaths rates; **(F)** the EAPC of RHD-related deaths between 1990 and 2021.

Across countries, Eritrea had the highest ASIR of RHD among children and youths aged 5–19 from 1990 [246.5 (159.65–353.8) per 100 000 population] to 2021 [252.88 (166.5–363.19) per 100 000 population], and increased 0.26% (0.01% to 0.51%) in EAPCs since 1990. The second highest ASIR attributable to RHD was in Central African Republic and Democratic Republic of the Congo in 1990 and 2019, increased 0.13% (0.02% to 0.14%) and 0.08% (0.03% to 0.12%) in EAPCs since 1990, respectively (Figures 3A, B). The highest and lowest age-standardized prevalence rate (ASPR) of RHD were in Congo and Sweden in 2021, with rates of 1,517.35 (1,018.72–2,121) and 2.79 (2.01–3.66) per 100,000 population, respectively (Figures 3C, D). Eritrea's persistently high ASIR may reflect limited access to primary healthcare and low uptake of prophylactic treatment for streptococcal pharyngitis. In contrast, Sweden's low rates likely reflect successful public health measures and early treatment programs. These country-level differences underscore the importance of health system capacity and socioeconomic development in shaping RHD burden.

Commensurate with the ASPRs, the highest age-standardized death rate (ASDR) of RHD in 2021 was at the Niue, followed by Tokelau (Figures 3E, F), with rates of 5.38 (4.16–6.88) and 5.29 (3.94–7.17) per 100 000 population, respectively. Norway and Sweden had the lowest ASDRs for RHD in 2021, with rates of 0.

### 3.4 RHD burden in different regions

**Figure 4:**
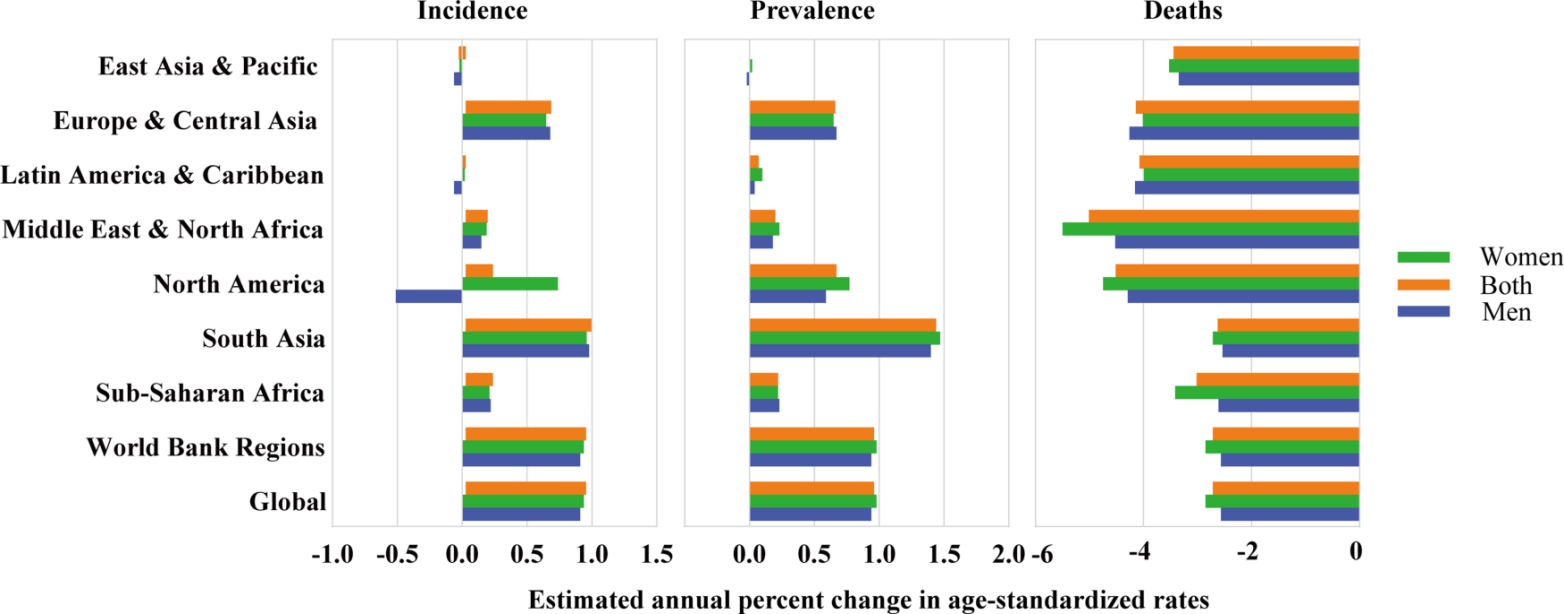
Estimated annual percent change in age-standardized incidence, prevalence, and death rate of RHD in children and youths by sex and world bank regions from 1990 to 2021.

When categorized by World Bank regions, from 1990 to 2021, 7 of 8 regions experienced a rise in the prevalence rate of RHDs, while 6 of 8 regions saw an increase in incidence rates (Figure 4 and Supplementary file S3). Compared with territories with East Asia & Pacific [0% (−0.25%, −0.24%)] and Latin America & Caribbean [0.07% (0.05%, 0.09%)], territories with Europe & Central Asia [0.66% (0.44%, 0.88%)], Middle East & North Africa [0.2% (0.05%, 0.36%)], North America [0.67% (0.34%, 1.01%)], South Asia [1.44% (1.08%, 1.8%)], Sub-Saharan Africa [0.22% (0.21%, 0.24%)], Global [0.96% (0.82%, 0.11%)] increased faster, and the South Asia region had the fastest increase in incidence and prevalence rate, but mortality rates decreased in all regions. Comparable findings were observed in both genders (Figure 3 and Supplementary file S4). When age groups were analyzed, the prevalence rate exhibited an increase from 1990 to 2019, with the highest EAPC recorded at 10–14 years (0.84 at 5–9 years, 0.99 at 10–14 years, and 0.91 at 15–19 years). Conversely, the mortality rate showed a decline across nearly all age groups, with the most significant EAPC noted in those aged 5–19 years (mortality: −4.04 at 5–9 years, −2.64 at 10–14 years, and −2.25 at 15–19 years). Analyzing data by sex, both males and females exhibited rising incidence and prevalence rates, alongside falling mortality rates, with a more pronounced reduction in the global mortality EAPC for women (−2.85%) relative to men (−2.56%) (Supplementary file S4). Changes in these metrics when stratified by age, sex, and Socio-Demographic Index (SDI) are illustrated in Supplementary files S1–S3. The impact of rheumatic heart disease (RHD) from 1990 to 2021, categorized by country, is displayed in Supplementary files S4.

## 4 Discussion

RHD continues to be the most prevalent form of valvular heart disease worldwide (18), yet it is frequently overlooked. In pediatric patients, RHD can lead to several adverse health outcomes, including congestive heart failure, irreversible injury to heart valves, arrhythmias, and thromboembolic events (19–21). Reliable data is crucial for disease modeling for epidemiological analysis (22). The GBD 2021 employs comprehensive and meticulous data collection and screening techniques to ensure that the estimates produced by modeling are trustworthy and comprehensible (15). For the GBD 2021, all data sources were consolidated and analyzed as a whole. Advanced methodologies were utilized to process the data, guaranteeing consistency and completeness across different demographics such as locations, age brackets, genders, years, and health conditions. This research provides a novel and trustworthy evaluation of the epidemiological characteristics of RHD among children and youths. The results can serve as a resource for governments in various regions to develop focused prevention and treatment approaches for RHD.

The study indicated that more than 11.6 million children and youths aged 5–19 years around the globe were impacted by RHD. During the course of the study, the total number of case count and ASRs for RHD incidence and prevalence rose worldwide among children and youths. In contrast, a decline in mortality rates was noted, likely due to swift advancements in medical diagnostic and therapeutic methods. Our analysis revealed a consistent increase in ASIRs from 1995 to 2015, potentially associated with the growing use of echocardiographic diagnosis and screening (23). From 2015 to 2021, a decline in ASIR was noted, potentially connected to the civil society initiative “RHD Action” that was introduced in 2015 (24, 25). This initiative aims to assist patient and community organizations in raising awareness, advocating for change, and enhancing the capabilities of healthcare workers to prevent and manage RHD in low- and middle-income countries (LMICs). Remarkably, the most pronounced rises in incidence and prevalence were observed in the 10–14 years age category, whereas the smaller reductions in mortality rates were noted in the 5–9 years age group. The significant reduction in RHD mortality among children aged 5–9 years may be due to the improvement of medical standards and the increased attention paid by parents. The burden of RHD, in comparative terms, was notably greater in children aged 10–14 years. The peak burden in the 10–14 age group may reflect increased school exposure to infections and the natural history of disease progression after repeated streptococcal infections during childhood. Therefore, this population group will be a primary focus for upcoming RHD prevention and therapeutic initiatives.

Regarding differences related to gender, our research indicated that the incidence of RHD was significantly higher in females than in males, which aligns with previous research findings (26). An Australian study involving 1,425 individuals revealed a pronounced 2-fold higher occurrence of RHD in women as opposed to men, highlighting the increased risk of autoimmunity in women (27, 28). Similarly, a Tanzanian study that assessed 4,436 school-aged children across 11 educational institutions also corroborated that the prevalence of RHD is elevated in females (29). The field of gender medicine has garnered interest concerning the understanding of cardiovascular diseases, as multiple studies have demonstrated variances between men and women regarding disease manifestations, treatment responses, and outcomes (30–33). While autoimmunity may contribute to higher RHD prevalence in females, disparities in healthcare access, education, and care-seeking behavior—especially in low-income settings—may also play a role. In some regions, girls may receive delayed or inadequate treatment for streptococcal infections, increasing their long-term risk. Nonetheless, it remains uncertain whether these gender disparities represent authentic biological differences or are indicative of a gender bias within healthcare, with females potentially receiving inadequate screening and preventive measures due to their socioeconomic circumstances (34). To summarize, further comprehensive research is needed on gender disparities in pediatric RHD.

It is widely recognized that the socioeconomic status significantly impacts pediatric RHD. According to the classification of SDI, it can be found that the burden of RHD is negatively correlated with economic level, and the incidence, prevalence and death of High SDI areas are the lowest, with no significant change between 2021 and 1990. The prevalence, prevalence and death of low-middle and Low regions are the highest. The incidence and prevalence in 2021 is significantly higher than that in 1990, but the mortality rate is lower (Table 1), which may be attributed to the progress of detection technology, especially the improvement of echocardiography has led to more subclinical RHD diagnoses (35). Early detection allows timely antibiotic prophylaxis, thus reducing progression and fatality. However, subclinical cases also inflate prevalence statistics while contributing less to mortality figures. Despite the availability of global diagnostic guidelines, diagnosing RHD remains challenging in tropical and subtropical areas. This difficulty arises due to the broad differential diagnoses for febrile illnesses accompanied by joint pain, low awareness among healthcare providers, and limited laboratory facilities (20, 36).

RHD continues to pose a significant public health issue, particularly in countries with low to middle incomes. Our research identified an inverse relationship between the burden of RHD and the SDI in children across all global regions, aligning with numerous earlier studies (37–40). In 2021, the number of incident rates in Eritrea, Central African Republic, Democratic Republic of the Congo were the most populous in the world, with 252.88, 244.87, and 241.12 rates respectively during 5–19 years. the number of incident rates in Finland, Austria, Denmark had the lowest incidence rates worldwide with all 0.13 rates. During the past more than 30 years, among the vulnerable groups in poorer countries, the incidence rate of RHD has been continuously increasing. In North Africa and the Middle East, sub-Saharan Africa east of the Sahara, and Southeast Asia regions, there has been a significant growth in the incidence rate and prevalence of RHD in children and youths. In the rural Ethiopian group of individuals aged 6–25 years, the statistical prevalence of RHD reached 37.5 per 1,000 population (95% CI: 26.9–51.8) (41). Overcrowded housing, inadequate living standards, and suboptimal public healthcare infrastructure may contribute to the elevated risk of RHD (24).

Additionally, echocardiography is crucial for the management of RHD. In 2004, the World Health Organization (WHO) advised the implementation of echocardiographic screening for RHD in areas with high prevalence. The first set of international evidence-based guidelines, which included clearly defined echocardiographic criteria for diagnosing RHD, was established in 2012. The criteria set forth by the World Heart Federation (WHF) have rapidly become the benchmark for echocardiographic screening, resulting in the detection of more subclinical cases of RHD (23). However, both research and practical applications of the WHF 2012 guidelines have revealed certain limitations within these criteria, particularly regarding the varied outcomes associated with echocardiography-detected RHD. In response, the WHF published revised criteria for the echocardiographic diagnosis of RHD in 2023 (42), which revise the fundamental criteria necessary for diagnosing the condition (43). From a long-term perspective, the extensive use of echocardiography significantly contributes to reducing the global incidence of RHD and transforming its management and prognosis. It is essential to utilize all accessible echocardiographic techniques to acquire precise anatomical and hemodynamic information regarding the affected valve lesion(s) to facilitate risk assessment, diagnostics, and pre-treatment strategy formulation (42).

RHD, resulting from acute rheumatic fever, is an illness characterized by fever and is caused by an infection from group A streptococcus (44). The use of secondary antibiotic prophylaxis has proven effective in hindering the advancement of latent RHD (45). Penicillin administration has been a primary contributor to the reduction in RHD mortality (41, 46).

This study presents several limitations. Firstly, we evaluated the burden of RHD in children taking into account age, gender, and geographical regions, but did not consider other potential risk factors. Moreover, the data quality utilized in this research is dependent on the reliability of the original GBD data collection methods, which means that some level of bias is unavoidable. It is advisable to further validate these findings through a larger cohort study. Lastly, the present investigation focused solely on the existing conditions of the pediatric population, moving forward, it will be essential to incorporate a broader array of data and develop pertinent models to forecast RHD prevalence in children and youths, thereby offering a more comprehensive foundation for its prevention and management. We found no clear risk factors for RHD in children and youths by GBD, which has limited implications for future treatment directions.

## 5 Conclusions

Although the age-standardized mortality rate for RHD among children and adolescents decreased substantially from 1990 to 2021, the incidence and prevalence rates continue to rise within the younger demographic, suggesting that the global burden of RHD remains substantial in this population. Certain areas, particularly nations/territories with low and middle SDI levels, Sub-Saharan Africa and South Asia, are experiencing alarming increases in the RHD burden in children and youths. The age group of 10–14 years is particularly critical and necessitates targeted interventions to alleviate the RHD disease burden, while also ensuring that attention is given to women. There is an urgent necessity to create and execute effective strategies and interventions designed to alleviate the burden of RHD burden in children and adolescents.

## Data Availability

The original contributions presented in the study are included in the article/Supplementary Material, further inquiries can be directed to the corresponding author.
