# Supplementary material for: Changes in the burden and underlying causes of rheumatic heart disease in children and youths, 1990–2021: an analysis of the Global Burden of Disease Study 2021
Source: Front Cardiovasc Med. 2025 Jun 26;12:1597855. doi: 10.3389/fcvm.2025.1597855 (PMC12241001; doi:10.3389/fcvm.2025.1597855)
Supplement: Supplementary file 1 [file Table1.docx]

Table S1. Incidence of Rheumatic heart diseasein 1990 and 2021 for Both sexes and all locations, with EAPC from 1990 and 2021.

| location | Num_1990 | ASR_1990 | Num_2021 | ASR_2021 | Num_change | EAPC_CI |
| --- | --- | --- | --- | --- | --- | --- |
| Andean Latin America | 18274 (12040 to 26123) | 133.64 (88.04 to 191.04) | 23823 (15645 to 34314) | 136 (89.31 to 195.88) | 0.3% (0.25 to 0.36) | 0% (0 to 0) |
| Australasia | 44 (24 to 71) | 0.93 (0.5 to 1.5) | 48 (27 to 81) | 0.84 (0.46 to 1.41) | 0.1% (-0.1 to 0.38) | 0% (0 to 0) |
| Caribbean | 13947 (9265 to 19862) | 127.17 (84.48 to 181.1) | 15910 (10557 to 22591) | 139.63 (92.65 to 198.26) | 0.14% (0.1 to 0.18) | 0% (0 to 0) |
| Central Asia | 22381 (14836 to 31792) | 101.48 (67.27 to 144.14) | 25798 (16697 to 36696) | 104.75 (67.8 to 149) | 0.15% (0.11 to 0.2) | 0.13% (0.03 to 0.22) |
| Central Europe | 1567 (1063 to 2175) | 5.2 (3.53 to 7.22) | 660 (451 to 910) | 3.67 (2.51 to 5.06) | -0.58% (-0.61 to -0.54) | -0.97% (-1.24 to -0.7) |
| Central Latin America | 34612 (22842 to 49344) | 58.06 (38.32 to 82.77) | 39149 (26094 to 56048) | 60.05 (40.02 to 85.97) | 0.13% (0.09 to 0.17) | 0% (0 to 0) |
| Central Sub-Saharan Africa | 48046 (31670 to 68070) | 233.19 (153.71 to 330.38) | 124401 (81352 to 179850) | 236.98 (154.97 to 342.61) | 1.59% (1.44 to 1.73) | 0% (0 to 0) |
| East Asia | 315157 (207897 to 453092) | 91.51 (60.37 to 131.56) | 200317 (131183 to 284686) | 75.63 (49.53 to 107.48) | -0.36% (-0.41 to -0.33) | -0.04% (-0.3 to 0.22) |
| Eastern Europe | 1105 (810 to 1466) | 2.21 (1.62 to 2.93) | 813 (567 to 1102) | 2.26 (1.57 to 3.06) | -0.26% (-0.33 to -0.19) | -0.23% (-0.37 to -0.09) |
| Eastern Sub-Saharan Africa | 146431 (96485 to 207602) | 195.73 (128.97 to 277.5) | 353490 (230563 to 513153) | 215.82 (140.77 to 313.3) | 1.41% (1.35 to 1.49) | 0% (0 to 0) |
| Global | 1277805 (850948 to 1804886) | 77.98 (51.93 to 110.15) | 1858128 (1227167 to 2650123) | 93.96 (62.05 to 134) | 0.45% (0.43 to 0.47) | 0% (0 to 0) |
| High-income Asia Pacific | 155 (87 to 264) | 0.39 (0.22 to 0.66) | 47 (22 to 82) | 0.19 (0.09 to 0.34) | -0.7% (-0.8 to -0.57) | 0% (0 to 0) |
| High-income North America | 313 (187 to 501) | 0.52 (0.31 to 0.83) | 343 (216 to 515) | 0.5 (0.31 to 0.75) | 0.1% (-0.05 to 0.35) | 0.21% (-0.16 to 0.58) |
| High-middle SDI | 100870 (67098 to 142927) | 36.38 (24.2 to 51.55) | 72825 (47942 to 102833) | 31.21 (20.55 to 44.07) | -0.28% (-0.31 to -0.25) | 0% (0 to 0) |
| High SDI | 3172 (2256 to 4229) | 1.67 (1.19 to 2.23) | 3217 (2278 to 4285) | 1.8 (1.27 to 2.4) | 0.01% (-0.06 to 0.09) | 0% (0 to 0) |
| Low-middle SDI | 391907 (261319 to 551531) | 93.86 (62.59 to 132.09) | 619750 (411329 to 892651) | 108.19 (71.81 to 155.84) | 0.58% (0.54 to 0.63) | 0% (0 to 0) |
| Low SDI | 269660 (177592 to 380898) | 142.84 (94.07 to 201.77) | 670070 (438121 to 962298) | 160.06 (104.66 to 229.87) | 1.48% (1.43 to 1.54) | 0% (0 to 0) |
| Middle SDI | 511277 (337486 to 730927) | 90.65 (59.84 to 129.59) | 490940 (323323 to 695473) | 85.74 (56.47 to 121.46) | -0.04% (-0.06 to -0.02) | 0% (0 to 0) |
| North Africa and Middle East | 71063 (47857 to 99839) | 56.61 (38.12 to 79.53) | 110261 (73167 to 154513) | 62.88 (41.73 to 88.11) | 0.55% (0.49 to 0.6) | 0% (0 to 0) |
| Oceania | 2715 (1829 to 3803) | 114.93 (77.43 to 161) | 5536 (3713 to 7814) | 124.35 (83.41 to 175.52) | 1.04% (0.95 to 1.17) | 0.27% (0.21 to 0.34) |
| South Asia | 298573 (199278 to 422796) | 77.47 (51.71 to 109.7) | 455443 (299350 to 654149) | 86.77 (57.03 to 124.62) | 0.53% (0.47 to 0.58) | 0% (0 to 0) |
| Southeast Asia | 70225 (46250 to 99113) | 43.45 (28.62 to 61.33) | 81485 (53404 to 116711) | 47.1 (30.87 to 67.47) | 0.16% (0.13 to 0.19) | 0.42% (0.33 to 0.5) |
| Southern Latin America | 10994 (7209 to 15456) | 77.25 (50.65 to 108.6) | 12637 (8338 to 17831) | 82.97 (54.74 to 117.07) | 0.15% (0.06 to 0.24) | 0.29% (0.24 to 0.34) |
| Southern Sub-Saharan Africa | 38425 (25067 to 54763) | 202.37 (132.02 to 288.41) | 47579 (31352 to 69014) | 204.77 (134.93 to 297.02) | 0.24% (0.21 to 0.28) | 0% (0 to 0) |
| Tropical Latin America | 83573 (54951 to 120316) | 160.16 (105.31 to 230.57) | 79079 (52122 to 113629) | 160.14 (105.55 to 230.11) | -0.05% (-0.08 to -0.02) | 0% (0 to 0) |
| Western Europe | 234 (132 to 406) | 0.31 (0.17 to 0.54) | 143 (68 to 247) | 0.2 (0.1 to 0.35) | -0.39% (-0.56 to -0.23) | -1.97% (-2.49 to -1.45) |
| Western Sub-Saharan Africa | 99970 (65040 to 142947) | 139.33 (90.65 to 199.23) | 281163 (182385 to 401902) | 149.06 (96.7 to 213.08) | 1.81% (1.77 to 1.86) | 0% (0 to 0) |
