# Supplementary material for: Changes in the burden and underlying causes of rheumatic heart disease in children and youths, 1990–2021: an analysis of the Global Burden of Disease Study 2021
Source: Front Cardiovasc Med. 2025 Jun 26;12:1597855. doi: 10.3389/fcvm.2025.1597855 (PMC12241001; doi:10.3389/fcvm.2025.1597855)
Supplement: Supplementary file 2 [file Table2.docx]

Table S2. Prevalence of Rheumatic heart diseasein 1990 and 2021 for Both sexes and all locations, with EAPC from 1990 and 2021.

| location | Num_1990 | ASR_1990 | Num_2021 | ASR_2021 | Num_change | EAPC_CI |
| --- | --- | --- | --- | --- | --- | --- |
| Andean Latin America | 116953 (79031 to 162085) | 855.25 (577.94 to 1185.3) | 156116 (105089 to 218482) | 891.18 (599.9 to 1247.2) | 0.33% (0.28 to 0.39) | 0% (0 to 0) |
| Australasia | 863 (579 to 1221) | 18.25 (12.24 to 25.82) | 1000 (710 to 1384) | 17.46 (12.41 to 24.17) | 0.16% (0.06 to 0.29) | 0% (0 to 0) |
| Caribbean | 92340 (62954 to 127023) | 841.94 (574 to 1158.17) | 102486 (69031 to 141857) | 899.44 (605.84 to 1244.97) | 0.11% (0.07 to 0.15) | 0% (0 to 0) |
| Central Asia | 145386 (99528 to 201273) | 659.19 (451.26 to 912.58) | 165600 (112798 to 229548) | 672.41 (458.01 to 932.08) | 0.14% (0.1 to 0.18) | 0.21% (0.05 to 0.37) |
| Central Europe | 11839 (8771 to 15104) | 39.28 (29.11 to 50.12) | 5595 (4186 to 7219) | 31.13 (23.29 to 40.17) | -0.53% (-0.56 to -0.49) | -0.53% (-0.75 to -0.31) |
| Central Latin America | 221462 (152125 to 303947) | 371.5 (255.19 to 509.86) | 255160 (174395 to 349225) | 391.38 (267.5 to 535.67) | 0.15% (0.12 to 0.18) | 0% (0 to 0) |
| Central Sub-Saharan Africa | 294207 (199912 to 408726) | 1427.93 (970.27 to 1983.74) | 764245 (506531 to 1066874) | 1455.86 (964.93 to 2032.36) | 1.6% (1.47 to 1.72) | 0% (0 to 0) |
| East Asia | 2329558 (1612326 to 3189514) | 676.43 (468.17 to 926.13) | 1403651 (965826 to 1937136) | 529.92 (364.63 to 731.33) | -0.4% (-0.43 to -0.37) | 0% (-0.32 to 0.32) |
| Eastern Europe | 11043 (8701 to 13670) | 22.07 (17.39 to 27.32) | 7766 (6109 to 9550) | 21.55 (16.95 to 26.5) | -0.3% (-0.35 to -0.25) | -0.37% (-0.49 to -0.25) |
| Eastern Sub-Saharan Africa | 864746 (584215 to 1202055) | 1155.91 (780.92 to 1606.79) | 2146796 (1430823 to 3004207) | 1310.71 (873.58 to 1834.2) | 1.48% (1.41 to 1.55) | 0% (0 to 0) |
| Global | 8168364 (5584392 to 11246055) | 498.49 (340.79 to 686.31) | 11637889 (7847347 to 16153391) | 588.46 (396.8 to 816.79) | 0.42% (0.4 to 0.44) | 0.96% (0.82 to 1.1) |
| High-income Asia Pacific | 3796 (2824 to 5008) | 9.46 (7.04 to 12.49) | 1846 (1447 to 2324) | 7.59 (5.95 to 9.55) | -0.51% (-0.57 to -0.44) | -1.07% (-1.26 to -0.87) |
| High-income North America | 5563 (4263 to 7343) | 9.26 (7.1 to 12.23) | 6476 (5236 to 8033) | 9.38 (7.58 to 11.63) | 0.16% (0.05 to 0.29) | 0% (0 to 0) |
| High-middle SDI | 756025 (531355 to 1028813) | 272.67 (191.64 to 371.06) | 530973 (371048 to 728652) | 227.58 (159.03 to 312.3) | -0.3% (-0.32 to -0.28) | 0% (0 to 0) |
| High SDI | 34336 (27243 to 41637) | 18.11 (14.37 to 21.96) | 33879 (26801 to 41063) | 18.94 (14.98 to 22.96) | -0.01% (-0.07 to 0.05) | 0% (0 to 0) |
| Low-middle SDI | 2297233 (1560464 to 3168461) | 550.18 (373.73 to 758.84) | 3755232 (2512847 to 5212971) | 655.58 (438.69 to 910.07) | 0.63% (0.59 to 0.67) | 0% (0 to 0) |
| Low SDI | 1587760 (1067586 to 2207905) | 841.06 (565.52 to 1169.56) | 4036178 (2698779 to 5631992) | 964.14 (644.67 to 1345.34) | 1.54% (1.5 to 1.6) | 0% (0 to 0) |
| Middle SDI | 3486785 (2390465 to 4815722) | 618.2 (423.83 to 853.82) | 3272833 (2227165 to 4522666) | 571.6 (388.97 to 789.88) | -0.06% (-0.08 to -0.04) | 0% (0 to 0) |
| North Africa and Middle East | 450234 (311946 to 616831) | 358.63 (248.48 to 491.33) | 701832 (474914 to 974062) | 400.24 (270.83 to 555.48) | 0.56% (0.5 to 0.62) | 0% (0 to 0) |
| Oceania | 21021 (14905 to 28656) | 889.87 (630.98 to 1213.12) | 42507 (29886 to 57939) | 954.8 (671.29 to 1301.42) | 1.02% (0.93 to 1.15) | 0.29% (0.2 to 0.37) |
| South Asia | 1651340 (1094831 to 2284301) | 428.47 (284.07 to 592.71) | 2623649 (1707047 to 3664912) | 499.84 (325.21 to 698.21) | 0.59% (0.53 to 0.64) | 0% (0 to 0) |
| Southeast Asia | 466919 (322405 to 641145) | 288.91 (199.49 to 396.72) | 562173 (385875 to 780316) | 324.98 (223.07 to 451.08) | 0.2% (0.17 to 0.23) | 0.54% (0.45 to 0.63) |
| Southern Latin America | 77240 (53457 to 104687) | 542.71 (375.6 to 735.56) | 90677 (62145 to 125260) | 595.34 (408.01 to 822.39) | 0.17% (0.1 to 0.25) | 0.36% (0.31 to 0.42) |
| Southern Sub-Saharan Africa | 245837 (166452 to 343069) | 1294.71 (876.62 to 1806.79) | 307236 (209837 to 426387) | 1322.28 (903.09 to 1835.08) | 0.25% (0.22 to 0.29) | 0% (0 to 0) |
| Tropical Latin America | 532542 (362152 to 732600) | 1020.54 (694.01 to 1403.92) | 520367 (353930 to 718573) | 1053.8 (716.75 to 1455.19) | -0.02% (-0.05 to 0) | 0% (0 to 0) |
| Western Europe | 5029 (3644 to 6759) | 6.67 (4.83 to 8.96) | 3654 (2653 to 4811) | 5.18 (3.76 to 6.83) | -0.27% (-0.36 to -0.2) | 0% (0 to 0) |
| Western Sub-Saharan Africa | 620447 (420787 to 867328) | 864.74 (586.46 to 1208.82) | 1769059 (1191290 to 2465671) | 937.91 (631.59 to 1307.23) | 1.85% (1.81 to 1.89) | 0% (0 to 0) |
