# Supplementary material for: Changes in the burden and underlying causes of rheumatic heart disease in children and youths, 1990–2021: an analysis of the Global Burden of Disease Study 2021
Source: Front Cardiovasc Med. 2025 Jun 26;12:1597855. doi: 10.3389/fcvm.2025.1597855 (PMC12241001; doi:10.3389/fcvm.2025.1597855)
Supplement: Supplementary file 3 [file Table3.docx]

Table S3. Deaths of Rheumatic heart diseasein 1990 and 2021 for Both sexes and all locations, with EAPC from 1990 and 2021.

| location | Num_1990 | ASR_1990 | Num_2021 | ASR_2021 | Num_change | EAPC_CI |
| --- | --- | --- | --- | --- | --- | --- |
| Andean Latin America | 50 (41 to 64) | 0.36 (0.3 to 0.47) | 15 (12 to 19) | 0.09 (0.07 to 0.11) | -0.69% (-0.78 to -0.58) | 0% (0 to 0) |
| Australasia | 7 (6 to 7) | 0.14 (0.13 to 0.15) | 2 (2 to 2) | 0.04 (0.03 to 0.04) | -0.67% (-0.72 to -0.62) | -4.26% (-4.55 to -3.97) |
| Caribbean | 158 (123 to 193) | 1.44 (1.12 to 1.76) | 94 (67 to 123) | 0.83 (0.58 to 1.08) | -0.41% (-0.55 to -0.21) | 0% (0 to 0) |
| Central Asia | 183 (167 to 201) | 0.83 (0.76 to 0.91) | 70 (60 to 82) | 0.29 (0.24 to 0.33) | -0.62% (-0.69 to -0.55) | -3.71% (-4.08 to -3.34) |
| Central Europe | 51 (49 to 54) | 0.17 (0.16 to 0.18) | 4 (4 to 5) | 0.02 (0.02 to 0.03) | -0.92% (-0.93 to -0.91) | 0% (0 to 0) |
| Central Latin America | 150 (143 to 156) | 0.25 (0.24 to 0.26) | 21 (16 to 24) | 0.03 (0.03 to 0.04) | -0.86% (-0.89 to -0.84) | 0% (0 to 0) |
| Central Sub-Saharan Africa | 145 (97 to 195) | 0.7 (0.47 to 0.94) | 138 (84 to 221) | 0.26 (0.16 to 0.42) | -0.05% (-0.42 to 0.48) | -3% (-3.15 to -2.84) |
| East Asia | 2210 (1845 to 2617) | 0.64 (0.54 to 0.76) | 176 (145 to 212) | 0.07 (0.05 to 0.08) | -0.92% (-0.94 to -0.89) | 0% (0 to 0) |
| Eastern Europe | 110 (108 to 113) | 0.22 (0.22 to 0.23) | 6 (5 to 6) | 0.02 (0.02 to 0.02) | -0.95% (-0.95 to -0.94) | 0% (0 to 0) |
| Eastern Sub-Saharan Africa | 400 (307 to 491) | 0.54 (0.41 to 0.66) | 343 (252 to 468) | 0.21 (0.15 to 0.29) | -0.14% (-0.4 to 0.16) | 0% (0 to 0) |
| Global | 20189 (16716 to 24209) | 1.23 (1.02 to 1.48) | 10198 (8981 to 11383) | 0.52 (0.45 to 0.58) | -0.49% (-0.57 to -0.4) | -2.71% (-2.9 to -2.52) |
| High-income Asia Pacific | 20 (17 to 22) | 0.05 (0.04 to 0.06) | 3 (2 to 3) | 0.01 (0.01 to 0.01) | -0.87% (-0.89 to -0.85) | -5.3% (-5.52 to -5.07) |
| High-income North America | 30 (29 to 31) | 0.05 (0.05 to 0.05) | 9 (9 to 10) | 0.01 (0.01 to 0.01) | -0.69% (-0.72 to -0.67) | -4.51% (-5.09 to -3.92) |
| High-middle SDI | 1117 (990 to 1296) | 0.4 (0.36 to 0.47) | 195 (172 to 221) | 0.08 (0.07 to 0.09) | -0.83% (-0.86 to -0.78) | 0% (0 to 0) |
| High SDI | 172 (155 to 193) | 0.09 (0.08 to 0.1) | 36 (32 to 40) | 0.02 (0.02 to 0.02) | -0.79% (-0.82 to -0.76) | 0% (0 to 0) |
| Low-middle SDI | 9962 (7949 to 12449) | 2.39 (1.9 to 2.98) | 5488 (4766 to 6288) | 0.96 (0.83 to 1.1) | -0.45% (-0.56 to -0.32) | 0% (0 to 0) |
| Low SDI | 3125 (2323 to 4099) | 1.66 (1.23 to 2.17) | 2585 (2125 to 3141) | 0.62 (0.51 to 0.75) | -0.17% (-0.33 to 0.05) | 0% (0 to 0) |
| Middle SDI | 5796 (5079 to 6461) | 1.03 (0.9 to 1.15) | 1881 (1644 to 2115) | 0.33 (0.29 to 0.37) | -0.68% (-0.74 to -0.61) | 0% (0 to 0) |
| North Africa and Middle East | 2379 (1584 to 3165) | 1.9 (1.26 to 2.52) | 770 (595 to 1030) | 0.44 (0.34 to 0.59) | -0.68% (-0.78 to -0.53) | 0% (0 to 0) |
| Oceania | 110 (62 to 162) | 4.67 (2.63 to 6.87) | 178 (121 to 258) | 4.01 (2.71 to 5.79) | 0.62% (0.23 to 1.3) | -0.44% (-0.6 to -0.28) |
| South Asia | 10622 (8521 to 13630) | 2.76 (2.21 to 3.54) | 6622 (5709 to 7690) | 1.26 (1.09 to 1.46) | -0.38% (-0.5 to -0.25) | 0% (0 to 0) |
| Southeast Asia | 2417 (1879 to 2840) | 1.5 (1.16 to 1.76) | 940 (727 to 1154) | 0.54 (0.42 to 0.67) | -0.61% (-0.71 to -0.5) | 0% (0 to 0) |
| Southern Latin America | 24 (22 to 26) | 0.17 (0.15 to 0.19) | 5 (4 to 5) | 0.03 (0.03 to 0.03) | -0.81% (-0.84 to -0.77) | 0% (0 to 0) |
| Southern Sub-Saharan Africa | 185 (156 to 216) | 0.98 (0.82 to 1.14) | 232 (182 to 293) | 1 (0.78 to 1.26) | 0.25% (-0.11 to 0.67) | 0% (0 to 0) |
| Tropical Latin America | 327 (305 to 351) | 0.63 (0.58 to 0.67) | 68 (59 to 75) | 0.14 (0.12 to 0.15) | -0.79% (-0.82 to -0.77) | -4.65% (-4.84 to -4.45) |
| Western Europe | 46 (44 to 48) | 0.06 (0.06 to 0.06) | 14 (13 to 14) | 0.02 (0.02 to 0.02) | -0.7% (-0.72 to -0.68) | 0% (0 to 0) |
| Western Sub-Saharan Africa | 564 (406 to 737) | 0.79 (0.57 to 1.03) | 488 (373 to 644) | 0.26 (0.2 to 0.34) | -0.13% (-0.35 to 0.17) | -3.47% (-3.62 to -3.32) |
