# Supplementary material for: Changes in the burden and underlying causes of rheumatic heart disease in children and youths, 1990–2021: an analysis of the Global Burden of Disease Study 2021
Source: Front Cardiovasc Med. 2025 Jun 26;12:1597855. doi: 10.3389/fcvm.2025.1597855 (PMC12241001; doi:10.3389/fcvm.2025.1597855)
Supplement: Supplementary file 5 [file Table5.docx]

Table S5. Incidence of Rheumatic heart diseasein 1990 and 2021 for Both sexes and all locations, with EAPC from 1990 and 2021.

| location | Num_1990 | ASR_1990 | Num_2021 | ASR_2021 | Num_change | EAPC_CI |
| --- | --- | --- | --- | --- | --- | --- |
| East Asia & Pacific - WB | 388700 (256190 to 556637) | 70.97 (46.78 to 101.63) | 287940 (189433 to 407548) | 61.64 (40.55 to 87.24) | -0.26% (-0.3 to -0.23) | -0.05% (-0.23 to 0.14) |
| Europe & Central Asia - WB | 24298 (16301 to 34246) | 12.53 (8.41 to 17.66) | 26323 (17230 to 37310) | 16.03 (10.49 to 22.72) | 0.08% (0.04 to 0.12) | 0.66% (0.47 to 0.85) |
| Global | 1277805 (850948 to 1804886) | 77.98 (51.93 to 110.15) | 1858128 (1227167 to 2650123) | 93.96 (62.05 to 134) | 0.45% (0.43 to 0.47) | 0.93% (0.81 to 1.04) |
| Latin America & Caribbean - WB | 160927 (106034 to 229871) | 107.08 (70.55 to 152.95) | 170060 (112419 to 243750) | 107.41 (71.01 to 153.96) | 0.06% (0.03 to 0.08) | -0.02% (-0.03 to -0.01) |
| Middle East & North Africa - WB | 63036 (42361 to 88340) | 65.62 (44.1 to 91.96) | 90045 (59318 to 126735) | 68.79 (45.32 to 96.82) | 0.43% (0.37 to 0.48) | 0.17% (0.07 to 0.27) |
| North America | 313 (187 to 501) | 0.52 (0.31 to 0.83) | 343 (216 to 515) | 0.5 (0.31 to 0.75) | 0.1% (-0.05 to 0.35) | 0.21% (-0.16 to 0.58) |
| South Asia - WB | 301799 (201411 to 427443) | 76.44 (51.01 to 108.26) | 465307 (305764 to 667986) | 85.75 (56.35 to 123.1) | 0.54% (0.48 to 0.6) | 0.97% (0.75 to 1.19) |
| Sub-Saharan Africa - WB | 337810 (221570 to 479000) | 174.26 (114.3 to 247.1) | 816785 (532232 to 1176105) | 184.13 (119.98 to 265.13) | 1.42% (1.38 to 1.46) | 0.21% (0.19 to 0.23) |
| World Bank Regions | 1276883 (850326 to 1803592) | 78.02 (51.95 to 110.2) | 1856802 (1226285 to 2648250) | 93.97 (62.06 to 134.03) | 0.45% (0.43 to 0.47) | 0.93% (0.81 to 1.04) |
