# Supplementary material for: Changes in the burden and underlying causes of rheumatic heart disease in children and youths, 1990–2021: an analysis of the Global Burden of Disease Study 2021
Source: Front Cardiovasc Med. 2025 Jun 26;12:1597855. doi: 10.3389/fcvm.2025.1597855 (PMC12241001; doi:10.3389/fcvm.2025.1597855)
Supplement: Supplementary file 6 [file Table6.docx]

Table S6. Incidence of Rheumatic heart diseasein 1990 and 2021 for Female sexes and all locations, with EAPC from 1990 and 2021.

| location | Num_1990 | ASR_1990 | Num_2021 | ASR_2021 | Num_change | EAPC_CI |
| --- | --- | --- | --- | --- | --- | --- |
| East Asia & Pacific - WB | 196606 (130674 to 277766) | 73.71 (48.99 to 104.14) | 143769 (94973 to 202722) | 64.79 (42.8 to 91.35) | -0.27% (-0.3 to -0.24) | -0.02% (-0.2 to 0.16) |
| Europe & Central Asia - WB | 12609 (8513 to 17811) | 13.29 (8.97 to 18.77) | 13473 (8922 to 19010) | 16.89 (11.19 to 23.83) | 0.07% (0.01 to 0.12) | 0.65% (0.45 to 0.85) |
| Global | 668581 (449675 to 942280) | 83.49 (56.15 to 117.67) | 972176 (648051 to 1391827) | 101.36 (67.56 to 145.11) | 0.45% (0.43 to 0.48) | 0.94% (0.83 to 1.06) |
| Latin America & Caribbean - WB | 86416 (57172 to 123381) | 115.49 (76.41 to 164.9) | 91155 (60520 to 129348) | 117.32 (77.89 to 166.48) | 0.05% (0.02 to 0.08) | 0.02% (0.01 to 0.03) |
| Middle East & North Africa - WB | 31530 (21446 to 43728) | 67.4 (45.85 to 93.48) | 44516 (29929 to 62054) | 70.32 (47.28 to 98.02) | 0.41% (0.35 to 0.47) | 0.19% (0.08 to 0.29) |
| North America | 168 (99 to 268) | 0.57 (0.34 to 0.91) | 204 (133 to 302) | 0.6 (0.39 to 0.89) | 0.21% (0.04 to 0.54) | 0.74% (0.31 to 1.18) |
| South Asia - WB | 163384 (108765 to 229895) | 86.04 (57.28 to 121.06) | 252794 (166657 to 363769) | 96.95 (63.92 to 139.52) | 0.55% (0.48 to 0.61) | 0.96% (0.73 to 1.18) |
| Sub-Saharan Africa - WB | 177376 (116139 to 252386) | 182.03 (119.18 to 259) | 425565 (279173 to 617384) | 192.46 (126.25 to 279.21) | 1.4% (1.35 to 1.45) | 0.21% (0.19 to 0.23) |
| World Bank Regions | 668090 (449339 to 941594) | 83.53 (56.18 to 117.72) | 971475 (647590 to 1390835) | 101.38 (67.58 to 145.14) | 0.45% (0.43 to 0.48) | 0.94% (0.83 to 1.06) |
