# Supplementary material for: Changes in the burden and underlying causes of rheumatic heart disease in children and youths, 1990–2021: an analysis of the Global Burden of Disease Study 2021
Source: Front Cardiovasc Med. 2025 Jun 26;12:1597855. doi: 10.3389/fcvm.2025.1597855 (PMC12241001; doi:10.3389/fcvm.2025.1597855)
Supplement: Supplementary file 7 [file Table7.docx]

Table S7. Incidence of Rheumatic heart diseasein 1990 and 2021 for Male sexes and all locations, with EAPC from 1990 and 2021.

| location | Num_1990 | ASR_1990 | Num_2021 | ASR_2021 | Num_change | EAPC_CI |
| --- | --- | --- | --- | --- | --- | --- |
| East Asia & Pacific - WB | 192093 (125631 to 277653) | 68.37 (44.71 to 98.82) | 144171 (94391 to 205057) | 58.78 (38.49 to 83.61) | -0.25% (-0.29 to -0.21) | -0.06% (-0.25 to 0.13) |
| Europe & Central Asia - WB | 11689 (7799 to 16384) | 11.8 (7.88 to 16.55) | 12850 (8329 to 18183) | 15.22 (9.87 to 21.54) | 0.1% (0.04 to 0.16) | 0.68% (0.5 to 0.86) |
| Global | 609224 (399713 to 867124) | 72.71 (47.71 to 103.5) | 885952 (579017 to 1264057) | 86.98 (56.85 to 124.11) | 0.45% (0.43 to 0.48) | 0.91% (0.8 to 1.02) |
| Latin America & Caribbean - WB | 74511 (48732 to 106327) | 98.74 (64.58 to 140.9) | 78905 (51611 to 114402) | 97.87 (64.01 to 141.89) | 0.06% (0.03 to 0.09) | -0.06% (-0.08 to -0.05) |
| Middle East & North Africa - WB | 31506 (21002 to 44584) | 63.93 (42.62 to 90.47) | 45529 (29799 to 64735) | 67.36 (44.09 to 95.77) | 0.45% (0.38 to 0.51) | 0.15% (0.06 to 0.25) |
| North America | 145 (81 to 236) | 0.47 (0.26 to 0.77) | 139 (79 to 216) | 0.39 (0.22 to 0.61) | -0.04% (-0.2 to 0.19) | -0.51% (-0.81 to -0.21) |
| South Asia - WB | 138415 (91570 to 196512) | 67.55 (44.69 to 95.9) | 212513 (138322 to 305765) | 75.39 (49.07 to 108.47) | 0.54% (0.47 to 0.61) | 0.98% (0.77 to 1.2) |
| Sub-Saharan Africa - WB | 160434 (104595 to 228565) | 166.41 (108.49 to 237.09) | 391219 (254082 to 561913) | 175.85 (114.21 to 252.57) | 1.44% (1.39 to 1.5) | 0.22% (0.2 to 0.23) |
| World Bank Regions | 608793 (399425 to 866513) | 72.75 (47.73 to 103.55) | 885326 (578599 to 1263185) | 87 (56.86 to 124.14) | 0.45% (0.43 to 0.48) | 0.91% (0.8 to 1.02) |
