# Supplementary material for: Changes in the burden and underlying causes of rheumatic heart disease in children and youths, 1990–2021: an analysis of the Global Burden of Disease Study 2021
Source: Front Cardiovasc Med. 2025 Jun 26;12:1597855. doi: 10.3389/fcvm.2025.1597855 (PMC12241001; doi:10.3389/fcvm.2025.1597855)
Supplement: Supplementary file 8 [file Table8.docx]

Table S8. Prevalence of Rheumatic heart diseasein 1990 and 2021 for Both sexes and all locations, with EAPC from 1990 and 2021.

| location | Num_1990 | ASR_1990 | Num_2021 | ASR_2021 | Num_change | EAPC_CI |
| --- | --- | --- | --- | --- | --- | --- |
| East Asia & Pacific - WB | 2823273 (1955953 to 3854533) | 515.48 (357.12 to 703.76) | 2012580 (1389169 to 2781882) | 430.81 (297.36 to 595.48) | -0.29% (-0.31 to -0.26) | 0% (-0.25 to 0.24) |
| Europe & Central Asia - WB | 169908 (121906 to 227374) | 87.62 (62.87 to 117.26) | 178076 (124237 to 242385) | 108.46 (75.67 to 147.63) | 0.05% (0.01 to 0.09) | 0.66% (0.44 to 0.88) |
| Global | 8168364 (5584392 to 11246055) | 498.49 (340.79 to 686.31) | 11637889 (7847347 to 16153391) | 588.46 (396.8 to 816.79) | 0.42% (0.4 to 0.44) | 0.96% (0.82 to 1.1) |
| Latin America & Caribbean - WB | 1037405 (709494 to 1425585) | 690.28 (472.09 to 948.58) | 1121332 (764111 to 1541302) | 708.26 (482.63 to 973.52) | 0.08% (0.06 to 0.1) | 0.07% (0.05 to 0.09) |
| Middle East & North Africa - WB | 398558 (276353 to 548040) | 414.91 (287.69 to 570.52) | 576717 (389328 to 801862) | 440.58 (297.43 to 612.58) | 0.45% (0.39 to 0.51) | 0.2% (0.05 to 0.36) |
| North America | 5563 (4263 to 7343) | 9.26 (7.1 to 12.23) | 6475 (5236 to 8033) | 9.38 (7.58 to 11.63) | 0.16% (0.05 to 0.29) | 0.67% (0.34 to 1.01) |
| South Asia - WB | 1671483 (1108863 to 2310825) | 423.35 (280.85 to 585.29) | 2682588 (1748168 to 3746136) | 494.37 (322.17 to 690.37) | 0.6% (0.55 to 0.66) | 1.44% (1.08 to 1.8) |
| Sub-Saharan Africa - WB | 2055904 (1390463 to 2865425) | 1060.56 (717.29 to 1478.16) | 5051303 (3389803 to 7064410) | 1138.71 (764.16 to 1592.52) | 1.46% (1.42 to 1.49) | 0.22% (0.21 to 0.24) |
| World Bank Regions | 8162094 (5579976 to 11237509) | 498.7 (340.93 to 686.61) | 11629071 (7841246 to 16141110) | 588.56 (396.85 to 816.92) | 0.42% (0.4 to 0.44) | 0.96% (0.82 to 1.1) |
