# Supplementary material for: Changes in the burden and underlying causes of rheumatic heart disease in children and youths, 1990–2021: an analysis of the Global Burden of Disease Study 2021
Source: Front Cardiovasc Med. 2025 Jun 26;12:1597855. doi: 10.3389/fcvm.2025.1597855 (PMC12241001; doi:10.3389/fcvm.2025.1597855)
Supplement: Supplementary file 9 [file Table9.docx]

Table S9. Prevalence of Rheumatic heart diseasein 1990 and 2021 for Female sexes and all locations, with EAPC from 1990 and 2021.

| location | Num_1990 | ASR_1990 | Num_2021 | ASR_2021 | Num_change | EAPC_CI |
| --- | --- | --- | --- | --- | --- | --- |
| East Asia & Pacific - WB | 1421626 (989372 to 1936767) | 532.98 (370.92 to 726.11) | 1001560 (695301 to 1382446) | 451.34 (313.33 to 622.98) | -0.3% (-0.32 to -0.27) | 0.02% (-0.22 to 0.26) |
| Europe & Central Asia - WB | 88844 (63715 to 118409) | 93.63 (67.15 to 124.78) | 92071 (64840 to 125005) | 115.43 (81.29 to 156.72) | 0.04% (-0.01 to 0.08) | 0.65% (0.42 to 0.88) |
| Global | 4210787 (2892525 to 5758194) | 525.82 (361.21 to 719.06) | 6007154 (4058969 to 8324675) | 626.3 (423.18 to 867.92) | 0.43% (0.4 to 0.45) | 0.98% (0.83 to 1.12) |
| Latin America & Caribbean - WB | 556328 (381946 to 760320) | 743.52 (510.46 to 1016.15) | 597234 (408941 to 821085) | 768.67 (526.33 to 1056.78) | 0.07% (0.05 to 0.1) | 0.1% (0.09 to 0.12) |
| Middle East & North Africa - WB | 199975 (139497 to 273536) | 427.49 (298.2 to 584.74) | 287441 (196169 to 396505) | 454.05 (309.87 to 626.33) | 0.44% (0.38 to 0.51) | 0.23% (0.07 to 0.38) |
| North America | 2622 (1982 to 3526) | 8.96 (6.77 to 12.05) | 3189 (2546 to 3995) | 9.45 (7.55 to 11.84) | 0.22% (0.08 to 0.36) | 0.77% (0.45 to 1.09) |
| South Asia - WB | 871752 (576620 to 1205021) | 459.06 (303.65 to 634.56) | 1414484 (923922 to 1967754) | 542.5 (354.35 to 754.7) | 0.62% (0.56 to 0.68) | 1.47% (1.1 to 1.83) |
| Sub-Saharan Africa - WB | 1066324 (723989 to 1479046) | 1094.29 (742.97 to 1517.83) | 2606558 (1752654 to 3642102) | 1178.79 (792.62 to 1647.11) | 1.44% (1.4 to 1.49) | 0.22% (0.2 to 0.24) |
| World Bank Regions | 4207473 (2890234 to 5753683) | 526.04 (361.35 to 719.36) | 6002536 (4055808 to 8318331) | 626.39 (423.24 to 868.05) | 0.43% (0.4 to 0.45) | 0.98% (0.83 to 1.12) |
