# Supplementary material for: Changes in the burden and underlying causes of rheumatic heart disease in children and youths, 1990–2021: an analysis of the Global Burden of Disease Study 2021
Source: Front Cardiovasc Med. 2025 Jun 26;12:1597855. doi: 10.3389/fcvm.2025.1597855 (PMC12241001; doi:10.3389/fcvm.2025.1597855)
Supplement: Supplementary file 10 [file Table10.docx]

Table 10. Prevalence of Rheumatic heart diseasein 1990 and 2021 for Male sexes and all locations, with EAPC from 1990 and 2021.

| location | Num_1990 | ASR_1990 | Num_2021 | ASR_2021 | Num_change | EAPC_CI |
| --- | --- | --- | --- | --- | --- | --- |
| East Asia & Pacific - WB | 1401647 (959298 to 1929049) | 498.86 (341.42 to 686.57) | 1011020 (693775 to 1398586) | 412.22 (282.87 to 570.25) | -0.28% (-0.31 to -0.25) | -0.02% (-0.27 to 0.23) |
| Europe & Central Asia - WB | 81065 (57930 to 108732) | 81.87 (58.5 to 109.81) | 86005 (59448 to 117207) | 101.87 (70.42 to 138.83) | 0.06% (0.01 to 0.11) | 0.67% (0.46 to 0.88) |
| Global | 3957577 (2696584 to 5472422) | 472.36 (321.85 to 653.16) | 5630736 (3793844 to 7823418) | 552.84 (372.49 to 768.12) | 0.42% (0.4 to 0.44) | 0.94% (0.8 to 1.09) |
| Latin America & Caribbean - WB | 481077 (325967 to 670181) | 637.5 (431.95 to 888.09) | 524098 (355415 to 731949) | 650.04 (440.82 to 907.83) | 0.09% (0.07 to 0.11) | 0.04% (0.02 to 0.06) |
| Middle East & North Africa - WB | 198583 (136824 to 272152) | 402.96 (277.64 to 552.25) | 289276 (193059 to 401904) | 427.97 (285.62 to 594.6) | 0.46% (0.38 to 0.53) | 0.18% (0.03 to 0.33) |
| North America | 2940 (2246 to 3807) | 9.55 (7.3 to 12.37) | 3287 (2668 to 4027) | 9.31 (7.56 to 11.4) | 0.12% (0.01 to 0.25) | 0.59% (0.23 to 0.94) |
| South Asia - WB | 799731 (533738 to 1107850) | 390.26 (260.46 to 540.62) | 1268104 (825571 to 1775369) | 449.85 (292.86 to 629.8) | 0.59% (0.53 to 0.64) | 1.4% (1.05 to 1.76) |
| Sub-Saharan Africa - WB | 989579 (662819 to 1382095) | 1026.47 (687.53 to 1433.62) | 2444745 (1637521 to 3425706) | 1098.87 (736.04 to 1539.79) | 1.47% (1.43 to 1.52) | 0.23% (0.21 to 0.24) |
| World Bank Regions | 3954621 (2694515 to 5468332) | 472.57 (321.99 to 653.45) | 5626535 (3790958 to 7817560) | 552.93 (372.54 to 768.25) | 0.42% (0.4 to 0.44) | 0.94% (0.8 to 1.09) |
