# Supplementary material for: Changes in the burden and underlying causes of rheumatic heart disease in children and youths, 1990–2021: an analysis of the Global Burden of Disease Study 2021
Source: Front Cardiovasc Med. 2025 Jun 26;12:1597855. doi: 10.3389/fcvm.2025.1597855 (PMC12241001; doi:10.3389/fcvm.2025.1597855)
Supplement: Supplementary file 11 [file Table11.docx]

Table S11. Deaths of Rheumatic heart diseasein 1990 and 2021 for Both sexes and all locations, with EAPC from 1990 and 2021.

| location | Num_1990 | ASR_1990 | Num_2021 | ASR_2021 | Num_change | EAPC_CI |
| --- | --- | --- | --- | --- | --- | --- |
| East Asia & Pacific - WB | 4698 (3893 to 5388) | 0.86 (0.71 to 0.98) | 1279 (1084 to 1497) | 0.27 (0.23 to 0.32) | -0.73% (-0.78 to -0.67) | -3.44% (-3.53 to -3.35) |
| Europe & Central Asia - WB | 478 (439 to 533) | 0.25 (0.23 to 0.28) | 115 (102 to 127) | 0.07 (0.06 to 0.08) | -0.76% (-0.8 to -0.72) | -4.14% (-4.35 to -3.93) |
| Global | 20189 (16716 to 24209) | 1.23 (1.02 to 1.48) | 10198 (8981 to 11383) | 0.52 (0.45 to 0.58) | -0.49% (-0.57 to -0.4) | -2.71% (-2.9 to -2.52) |
| Latin America & Caribbean - WB | 703 (655 to 760) | 0.47 (0.44 to 0.51) | 199 (172 to 231) | 0.13 (0.11 to 0.15) | -0.72% (-0.76 to -0.68) | -4.07% (-4.18 to -3.95) |
| Middle East & North Africa - WB | 2038 (1361 to 2723) | 2.12 (1.42 to 2.84) | 550 (434 to 743) | 0.42 (0.33 to 0.57) | -0.73% (-0.82 to -0.59) | -5.01% (-5.11 to -4.92) |
| North America | 30 (29 to 31) | 0.05 (0.05 to 0.05) | 9 (9 to 10) | 0.01 (0.01 to 0.01) | -0.69% (-0.72 to -0.67) | -4.51% (-5.09 to -3.93) |
| South Asia - WB | 10758 (8638 to 13795) | 2.72 (2.19 to 3.49) | 6729 (5817 to 7824) | 1.24 (1.07 to 1.44) | -0.37% (-0.49 to -0.25) | -2.62% (-2.89 to -2.35) |
| Sub-Saharan Africa - WB | 1464 (1126 to 1789) | 0.76 (0.58 to 0.92) | 1304 (1056 to 1664) | 0.29 (0.24 to 0.38) | -0.11% (-0.31 to 0.16) | -3.01% (-3.04 to -2.98) |
| World Bank Regions | 20170 (16700 to 24186) | 1.23 (1.02 to 1.48) | 10184 (8970 to 11366) | 0.52 (0.45 to 0.58) | -0.5% (-0.57 to -0.4) | -2.71% (-2.9 to -2.52) |
