# Supplementary material for: Changes in the burden and underlying causes of rheumatic heart disease in children and youths, 1990–2021: an analysis of the Global Burden of Disease Study 2021
Source: Front Cardiovasc Med. 2025 Jun 26;12:1597855. doi: 10.3389/fcvm.2025.1597855 (PMC12241001; doi:10.3389/fcvm.2025.1597855)
Supplement: Supplementary file 12 [file Table12.docx]

Table S12. Deaths of Rheumatic heart diseasein 1990 and 2021 for Female sexes and all locations, with EAPC from 1990 and 2021.

| location | Num_1990 | ASR_1990 | Num_2021 | ASR_2021 | Num_change | EAPC_CI |
| --- | --- | --- | --- | --- | --- | --- |
| East Asia & Pacific - WB | 2463 (1791 to 3056) | 0.92 (0.67 to 1.15) | 643 (528 to 803) | 0.29 (0.24 to 0.36) | -0.74% (-0.8 to -0.65) | -3.52% (-3.58 to -3.45) |
| Europe & Central Asia - WB | 238 (208 to 297) | 0.25 (0.22 to 0.31) | 57 (50 to 64) | 0.07 (0.06 to 0.08) | -0.76% (-0.82 to -0.7) | -4.01% (-4.2 to -3.82) |
| Global | 10394 (7535 to 13621) | 1.3 (0.94 to 1.7) | 5124 (4500 to 5791) | 0.53 (0.47 to 0.6) | -0.51% (-0.61 to -0.34) | -2.85% (-3.01 to -2.69) |
| Latin America & Caribbean - WB | 372 (333 to 422) | 0.5 (0.44 to 0.56) | 108 (84 to 135) | 0.14 (0.11 to 0.17) | -0.71% (-0.77 to -0.65) | -3.99% (-4.13 to -3.84) |
| Middle East & North Africa - WB | 1119 (593 to 1760) | 2.39 (1.27 to 3.76) | 261 (196 to 363) | 0.41 (0.31 to 0.57) | -0.77% (-0.86 to -0.56) | -5.49% (-5.61 to -5.36) |
| North America | 15 (15 to 16) | 0.05 (0.05 to 0.05) | 4 (4 to 5) | 0.01 (0.01 to 0.01) | -0.71% (-0.73 to -0.7) | -4.74% (-5.38 to -4.09) |
| South Asia - WB | 5386 (3805 to 7512) | 2.84 (2 to 3.96) | 3426 (2900 to 4014) | 1.31 (1.11 to 1.54) | -0.36% (-0.52 to -0.14) | -2.71% (-2.95 to -2.47) |
| Sub-Saharan Africa - WB | 790 (525 to 1036) | 0.81 (0.54 to 1.06) | 617 (477 to 780) | 0.28 (0.22 to 0.35) | -0.22% (-0.44 to 0.11) | -3.41% (-3.46 to -3.37) |
| World Bank Regions | 10383 (7527 to 13606) | 1.3 (0.94 to 1.7) | 5116 (4493 to 5782) | 0.53 (0.47 to 0.6) | -0.51% (-0.61 to -0.34) | -2.85% (-3.01 to -2.69) |
