# Supplementary material for: Changes in the burden and underlying causes of rheumatic heart disease in children and youths, 1990–2021: an analysis of the Global Burden of Disease Study 2021
Source: Front Cardiovasc Med. 2025 Jun 26;12:1597855. doi: 10.3389/fcvm.2025.1597855 (PMC12241001; doi:10.3389/fcvm.2025.1597855)
Supplement: Supplementary file 13 [file Table13.docx]

Table S13. Deaths of Rheumatic heart diseasein 1990 and 2021 for Male sexes and all locations, with EAPC from 1990 and 2021.

| location | Num_1990 | ASR_1990 | Num_2021 | ASR_2021 | Num_change | EAPC_CI |
| --- | --- | --- | --- | --- | --- | --- |
| East Asia & Pacific - WB | 2235 (1702 to 2620) | 0.8 (0.61 to 0.93) | 636 (501 to 800) | 0.26 (0.2 to 0.33) | -0.72% (-0.77 to -0.64) | -3.34% (-3.47 to -3.2) |
| Europe & Central Asia - WB | 240 (217 to 268) | 0.24 (0.22 to 0.27) | 58 (50 to 66) | 0.07 (0.06 to 0.08) | -0.76% (-0.8 to -0.72) | -4.26% (-4.51 to -4.01) |
| Global | 9795 (8409 to 11494) | 1.17 (1 to 1.37) | 5074 (4310 to 5945) | 0.5 (0.42 to 0.58) | -0.48% (-0.56 to -0.39) | -2.56% (-2.78 to -2.33) |
| Latin America & Caribbean - WB | 331 (309 to 355) | 0.44 (0.41 to 0.47) | 91 (74 to 109) | 0.11 (0.09 to 0.13) | -0.72% (-0.78 to -0.67) | -4.15% (-4.25 to -4.04) |
| Middle East & North Africa - WB | 919 (546 to 1416) | 1.86 (1.11 to 2.87) | 288 (221 to 408) | 0.43 (0.33 to 0.6) | -0.69% (-0.81 to -0.45) | -4.52% (-4.61 to -4.42) |
| North America | 15 (14 to 15) | 0.05 (0.05 to 0.05) | 5 (4 to 5) | 0.01 (0.01 to 0.01) | -0.67% (-0.71 to -0.64) | -4.29% (-4.84 to -3.75) |
| South Asia - WB | 5372 (4289 to 6883) | 2.62 (2.09 to 3.36) | 3303 (2699 to 4173) | 1.17 (0.96 to 1.48) | -0.39% (-0.5 to -0.25) | -2.53% (-2.84 to -2.21) |
| Sub-Saharan Africa - WB | 675 (533 to 832) | 0.7 (0.55 to 0.86) | 687 (529 to 926) | 0.31 (0.24 to 0.42) | 0.02% (-0.21 to 0.42) | -2.61% (-2.68 to -2.54) |
| World Bank Regions | 9787 (8402 to 11485) | 1.17 (1 to 1.37) | 5068 (4305 to 5938) | 0.5 (0.42 to 0.58) | -0.48% (-0.56 to -0.39) | -2.56% (-2.79 to -2.33) |
