# Supplementary material for: Changes in the burden and underlying causes of rheumatic heart disease in children and youths, 1990–2021: an analysis of the Global Burden of Disease Study 2021
Source: Front Cardiovasc Med. 2025 Jun 26;12:1597855. doi: 10.3389/fcvm.2025.1597855 (PMC12241001; doi:10.3389/fcvm.2025.1597855)
Supplement: Supplementary file 14 [file Table14.docx]

Table S14. Incidence of Rheumatic heart disease in 1990 and 2021 for both sexes in 204 countries, with EAPC from 1990 and 2021.

| location | Num_1990 | ASR_1990 | Num_2021 | ASR_2021 | EAPC_CI |
| --- | --- | --- | --- | --- | --- |
| Afghanistan | 3100.8 (2036.77 to 4386.31) | 80.36 (52.78 to 113.67) | 9732.15 (6466.11 to 13535.1) | 79.15 (52.59 to 110.08) | 0.04% (-0.04 to 0.12) |
| Albania | 1382.04 (902.09 to 1921.65) | 132.62 (86.56 to 184.39) | 598.39 (391.97 to 846.82) | 126.1 (82.6 to 178.46) | -0.13% (-0.15 to -0.1) |
| Algeria | 4577.92 (3215.79 to 6459.24) | 46.81 (32.88 to 66.04) | 7116.77 (4696.12 to 10132.47) | 60.6 (39.99 to 86.28) | 0.84% (0.53 to 1.15) |
| American Samoa | 19.51 (13.25 to 27.15) | 119.68 (81.28 to 166.51) | 19.55 (13.13 to 27.47) | 126.79 (85.16 to 178.12) | 0.12% (0.1 to 0.14) |
| Andorra | 0.02 (0.01 to 0.04) | 0.21 (0.08 to 0.41) | 0.02 (0.01 to 0.05) | 0.19 (0.07 to 0.38) | -0.08% (-0.13 to -0.03) |
| Angola | 8681.43 (5709.44 to 12343.26) | 227.63 (149.7 to 323.64) | 29599.37 (19435.81 to 41855.9) | 226.95 (149.02 to 320.92) | -0.03% (-0.05 to -0.01) |
| Antigua and Barbuda | 23.89 (15.9 to 33.59) | 134.01 (89.16 to 188.4) | 24.52 (16.41 to 34.6) | 135.55 (90.7 to 191.24) | 0.06% (0.04 to 0.08) |
| Argentina | 10947.68 (7162.08 to 15413.36) | 114.16 (74.68 to 160.72) | 12601.87 (8311.56 to 17794.6) | 117.61 (77.57 to 166.07) | 0.13% (0.09 to 0.17) |
| Armenia | 1275.86 (840.92 to 1793.34) | 135.03 (89 to 189.79) | 730.63 (477.29 to 1029.07) | 126.65 (82.73 to 178.38) | -0.3% (-0.37 to -0.23) |
| Australia | 33.42 (16.5 to 55.18) | 0.85 (0.42 to 1.41) | 38.96 (19.88 to 67.53) | 0.82 (0.42 to 1.43) | -0.1% (-0.23 to 0.03) |
| Austria | 5.42 (2.7 to 9.6) | 0.38 (0.19 to 0.67) | 1.77 (0.72 to 3.14) | 0.13 (0.05 to 0.24) | -4.35% (-5.27 to -3.41) |
| Azerbaijan | 2847.7 (1853.54 to 4055.14) | 127.91 (83.26 to 182.14) | 2847.43 (1836.3 to 4134.88) | 122.43 (78.96 to 177.79) | -0.18% (-0.25 to -0.12) |
| Bahamas | 110.21 (72.56 to 156.37) | 134.15 (88.32 to 190.33) | 132.63 (85.79 to 189.54) | 140.78 (91.07 to 201.19) | 0.1% (0.08 to 0.13) |
| Bahrain | 1.35 (0.71 to 2.34) | 0.97 (0.51 to 1.69) | 2.37 (1.19 to 4.13) | 0.76 (0.38 to 1.33) | -1.09% (-1.19 to -0.98) |
| Bangladesh | 27989.17 (19557.68 to 39649.9) | 67.56 (47.21 to 95.71) | 36922.05 (24635.88 to 51916.4) | 79.19 (52.84 to 111.35) | 0.51% (0.45 to 0.57) |
| Barbados | 87.45 (56.68 to 125.4) | 133.87 (86.77 to 191.97) | 74.21 (48.47 to 105.51) | 140.65 (91.86 to 199.96) | 0.16% (0.12 to 0.2) |
| Belarus | 20.25 (10.87 to 35.45) | 0.87 (0.47 to 1.52) | 9.48 (3.88 to 17.74) | 0.61 (0.25 to 1.14) | -1.33% (-1.49 to -1.18) |
| Belgium | 4.38 (1.78 to 8.77) | 0.23 (0.09 to 0.47) | 4.7 (1.84 to 9.07) | 0.24 (0.09 to 0.46) | -0.99% (-2.39 to 0.42) |
| Belize | 100.95 (65.66 to 142.72) | 138.22 (89.91 to 195.42) | 187.62 (123.72 to 271.14) | 143.31 (94.5 to 207.11) | 0.11% (0.1 to 0.13) |
| Benin | 2478.03 (1601.98 to 3570.16) | 131.6 (85.08 to 189.6) | 7193.37 (4686.91 to 10360.12) | 139.03 (90.59 to 200.24) | 0.18% (0.15 to 0.22) |
| Bermuda | 0.13 (0.07 to 0.21) | 1.09 (0.63 to 1.79) | 0.06 (0.03 to 0.11) | 0.7 (0.35 to 1.2) | -1.81% (-2.01 to -1.62) |
| Bhutan | 182.19 (120.78 to 255.2) | 74.32 (49.27 to 104.1) | 148.94 (98.59 to 210.38) | 76.8 (50.83 to 108.47) | 0.14% (0.11 to 0.17) |
| Bolivia (Plurinational State of) | 3255.02 (2156.42 to 4715.73) | 139.17 (92.2 to 201.62) | 4693.03 (3067.88 to 6726.55) | 139.98 (91.51 to 200.64) | 0.04% (0.02 to 0.05) |
| Bosnia and Herzegovina | 3.43 (1.51 to 6.37) | 0.3 (0.13 to 0.56) | 1.48 (0.64 to 2.83) | 0.29 (0.12 to 0.55) | -0.15% (-0.27 to -0.04) |
| Botswana | 991.18 (645.89 to 1454.05) | 187.92 (122.45 to 275.68) | 1259.26 (825.84 to 1798.9) | 184.77 (121.18 to 263.96) | -0.06% (-0.07 to -0.05) |
| Brazil | 81537.94 (53611.09 to 117405.32) | 160.73 (105.68 to 231.43) | 76214.29 (50223.76 to 109526.86) | 160.93 (106.05 to 231.27) | -0.01% (-0.04 to 0.01) |
| Brunei Darussalam | 0.52 (0.25 to 0.88) | 0.65 (0.31 to 1.1) | 0.38 (0.16 to 0.71) | 0.38 (0.17 to 0.72) | -2.27% (-2.44 to -2.09) |
| Bulgaria | 11.4 (5.57 to 19.66) | 0.62 (0.3 to 1.08) | 3.43 (1.53 to 6.31) | 0.35 (0.15 to 0.64) | -2.32% (-2.59 to -2.05) |
| Burkina Faso | 4980.38 (3238.42 to 7107.95) | 131.73 (85.66 to 188.01) | 11747.59 (7672.25 to 16683.48) | 135.04 (88.19 to 191.78) | 0.06% (0.05 to 0.07) |
| Burundi | 4216.62 (2781.46 to 6107.96) | 202.72 (133.72 to 293.65) | 11131.08 (7206.59 to 16089.77) | 217.65 (140.91 to 314.61) | 0.25% (0.23 to 0.28) |
| Cabo Verde | 186.81 (123.47 to 267.4) | 138.42 (91.48 to 198.12) | 205.61 (133.58 to 296.62) | 138.29 (89.84 to 199.49) | 0.02% (0 to 0.04) |
| Cambodia | 3315.49 (2148.12 to 4708.65) | 85.71 (55.53 to 121.72) | 4077.92 (2687.42 to 5827.57) | 83.52 (55.04 to 119.35) | -0.24% (-0.4 to -0.08) |
| Cameroon | 4980.86 (3253.96 to 7121.62) | 126.72 (82.78 to 181.18) | 16786.27 (10788.6 to 24043.18) | 139.43 (89.61 to 199.71) | 0.27% (0.21 to 0.34) |
| Canada | 42.27 (20.8 to 74.57) | 0.74 (0.36 to 1.3) | 43.42 (21.52 to 75.58) | 0.69 (0.34 to 1.19) | -0.23% (-0.27 to -0.19) |
| Central African Republic | 2367.16 (1536.6 to 3350.76) | 238.28 (154.67 to 337.29) | 5032.52 (3226.12 to 7230.23) | 244.87 (156.98 to 351.81) | 0.13% (0.12 to 0.14) |
| Chad | 3062.25 (1968.42 to 4406.42) | 134.17 (86.24 to 193.06) | 9890.71 (6332.44 to 14184.93) | 135.23 (86.58 to 193.94) | 0.04% (0.02 to 0.05) |
| Chile | 39.31 (21.45 to 68.49) | 1.02 (0.56 to 1.78) | 29.15 (14.84 to 50.94) | 0.77 (0.39 to 1.34) | -1.2% (-1.38 to -1.03) |
| China | 310285.19 (204578.57 to 446420.56) | 93.11 (61.39 to 133.97) | 196004.75 (128460.92 to 278931.64) | 76.37 (50.06 to 108.69) | -0.05% (-0.31 to 0.22) |
| Colombia | 108.94 (60.53 to 183.39) | 1 (0.56 to 1.69) | 96.51 (50.89 to 168.47) | 0.87 (0.46 to 1.52) | -0.58% (-0.8 to -0.37) |
| Comoros | 372.11 (247.65 to 530.55) | 204.57 (136.15 to 291.67) | 499.42 (326.36 to 738.91) | 214.67 (140.29 to 317.62) | 0.2% (0.18 to 0.21) |
| Congo | 2170.9 (1426.34 to 3111.9) | 232.88 (153.01 to 333.83) | 4354.88 (2856.85 to 6277.59) | 234.89 (154.09 to 338.6) | -0.41% (-0.65 to -0.16) |
| Cook Islands | 0.12 (0.07 to 0.19) | 1.89 (1.04 to 3) | 0.08 (0.04 to 0.12) | 1.86 (0.99 to 2.93) | -0.07% (-0.29 to 0.14) |
| Costa Rica | 1410.58 (923.65 to 2024.9) | 139.36 (91.25 to 200.05) | 1493.72 (990.38 to 2110.89) | 140.83 (93.37 to 199.01) | 0.05% (0.03 to 0.07) |
| Côte d'Ivoire | 6210.05 (4023.8 to 8875.8) | 134.77 (87.32 to 192.62) | 13960.2 (8976.3 to 19929.02) | 139.18 (89.49 to 198.69) | 0.09% (0.07 to 0.11) |
| Croatia | 2.29 (0.84 to 4.59) | 0.22 (0.08 to 0.45) | 0.86 (0.31 to 1.57) | 0.14 (0.05 to 0.25) | -1.8% (-2.95 to -0.63) |
| Cuba | 3829.07 (2599.79 to 5466.59) | 138.89 (94.3 to 198.28) | 2539.61 (1692.19 to 3579.61) | 136.78 (91.14 to 192.79) | -0.06% (-0.09 to -0.04) |
| Cyprus | 0.32 (0.11 to 0.64) | 0.16 (0.06 to 0.33) | 0.35 (0.13 to 0.69) | 0.16 (0.06 to 0.33) | -0.05% (-0.42 to 0.32) |
| Czechia | 7.41 (2.66 to 13.82) | 0.31 (0.11 to 0.58) | 4.98 (2.07 to 9.36) | 0.3 (0.12 to 0.56) | -2.19% (-3.8 to -0.55) |
| Democratic People's Republic of Korea | 4838.38 (3169.93 to 6743.01) | 88.73 (58.13 to 123.66) | 4286.96 (2794.34 to 6018.64) | 84.24 (54.91 to 118.26) | -0.23% (-0.26 to -0.2) |
| Democratic Republic of the Congo | 33723.62 (22328.85 to 47955.17) | 234.93 (155.55 to 334.08) | 82815.38 (53861.09 to 121039.15) | 241.12 (156.82 to 352.41) | 0.08% (0.03 to 0.12) |
| Denmark | 1.42 (0.54 to 2.87) | 0.15 (0.06 to 0.3) | 1.3 (0.52 to 2.69) | 0.13 (0.05 to 0.27) | -0.2% (-1.03 to 0.63) |
| Djibouti | 318.96 (210.03 to 465.42) | 199.8 (131.56 to 291.54) | 795.67 (511.84 to 1133.37) | 207.92 (133.75 to 296.17) | 0.18% (0.16 to 0.2) |
| Dominica | 34.63 (22.92 to 49.73) | 143.63 (95.07 to 206.26) | 23.32 (15.34 to 33.42) | 146.24 (96.18 to 209.58) | 0.04% (0.02 to 0.05) |
| Dominican Republic | 3489.46 (2303.57 to 5008.7) | 139.23 (91.91 to 199.84) | 3935.33 (2573.98 to 5452.52) | 137.72 (90.08 to 190.81) | -0.02% (-0.03 to 0) |
| Ecuador | 4785.34 (3090.94 to 6866.24) | 132.49 (85.58 to 190.1) | 6673.31 (4372.64 to 9711.42) | 134.67 (88.24 to 195.99) | 0.07% (0.06 to 0.08) |
| Egypt | 16823.01 (10979.1 to 23619.5) | 86.76 (56.62 to 121.81) | 28884.7 (19140.63 to 40668.83) | 86.19 (57.11 to 121.35) | 0.18% (0.07 to 0.29) |
| El Salvador | 2753.79 (1784.74 to 3985.41) | 138.71 (89.9 to 200.74) | 2476.89 (1627.29 to 3493.77) | 139.05 (91.35 to 196.13) | 0.07% (0.03 to 0.1) |
| Equatorial Guinea | 368.09 (244.03 to 530.14) | 233.38 (154.72 to 336.12) | 1276.92 (835.44 to 1807.65) | 219.1 (143.35 to 310.17) | -0.3% (-0.39 to -0.22) |
| Eritrea | 3311.23 (2144.63 to 4752.54) | 246.5 (159.65 to 353.8) | 5812.67 (3827.25 to 8348.43) | 252.88 (166.5 to 363.19) | 0.26% (0.01 to 0.51) |
| Estonia | 2.36 (1.12 to 4.09) | 0.7 (0.33 to 1.21) | 1.2 (0.49 to 2.29) | 0.57 (0.23 to 1.08) | -0.71% (-0.95 to -0.47) |
| Eswatini | 613.25 (400 to 887.63) | 184.87 (120.59 to 267.59) | 739.19 (478.34 to 1043.23) | 187.33 (121.22 to 264.38) | 0.08% (0.06 to 0.09) |
| Ethiopia | 33193.43 (21744.93 to 47511.83) | 167.41 (109.67 to 239.62) | 88489.98 (57801.92 to 128660) | 214.61 (140.18 to 312.03) | 1.04% (0.9 to 1.18) |
| Fiji | 187.79 (98.98 to 264.11) | 71.01 (37.42 to 99.86) | 256.44 (170.99 to 357.9) | 98.79 (65.87 to 137.87) | 1.76% (1.19 to 2.33) |
| Finland | 1.46 (0.48 to 2.92) | 0.15 (0.05 to 0.31) | 1.21 (0.46 to 2.51) | 0.13 (0.05 to 0.28) | -0.07% (-1.13 to 1) |
| France | 26.41 (9.01 to 54.54) | 0.22 (0.07 to 0.45) | 24.38 (8.12 to 45.52) | 0.2 (0.07 to 0.37) | -0.34% (-0.39 to -0.29) |
| Gabon | 735.07 (479.75 to 1048.92) | 208.83 (136.3 to 298) | 1321.61 (865.02 to 1869.07) | 215.42 (140.99 to 304.65) | 0.14% (0.12 to 0.16) |
| Gambia | 507.51 (329.41 to 733.18) | 134.94 (87.59 to 194.94) | 1326.2 (859.78 to 1958.93) | 145.89 (94.58 to 215.5) | 0.24% (0.22 to 0.25) |
| Georgia | 1730.23 (1159.64 to 2506.54) | 130.26 (87.31 to 188.71) | 869.87 (574.19 to 1224.86) | 126.8 (83.7 to 178.55) | -0.09% (-0.12 to -0.05) |
| Germany | 29.82 (10.57 to 59.23) | 0.23 (0.08 to 0.46) | 23.95 (7.67 to 46.94) | 0.2 (0.06 to 0.39) | -2.83% (-4.63 to -0.98) |
| Ghana | 7608.45 (5018.95 to 10843.73) | 135.25 (89.22 to 192.76) | 15809.23 (10315.39 to 22578.89) | 135.42 (88.36 to 193.41) | 0.02% (0 to 0.04) |
| Greece | 4.4 (1.85 to 8.31) | 0.2 (0.08 to 0.37) | 2.41 (0.96 to 4.61) | 0.16 (0.06 to 0.31) | -0.81% (-0.92 to -0.7) |
| Greenland | 0.1 (0.05 to 0.18) | 0.84 (0.42 to 1.46) | 0.08 (0.04 to 0.15) | 0.71 (0.35 to 1.29) | -0.7% (-0.77 to -0.63) |
| Grenada | 42.91 (28.43 to 61.34) | 142.32 (94.29 to 203.44) | 33.36 (21.89 to 46.89) | 143.54 (94.22 to 201.78) | 0% (-0.02 to 0.02) |
| Guam | 40.8 (27.89 to 56.34) | 107.44 (73.45 to 148.37) | 40.75 (26.84 to 57.52) | 114.12 (75.18 to 161.08) | 0.1% (0.08 to 0.12) |
| Guatemala | 4413.23 (2902.85 to 6193.48) | 130.69 (85.97 to 183.42) | 7322.12 (4785.55 to 10421.75) | 144.83 (94.66 to 206.14) | 0.45% (0.4 to 0.51) |
| Guinea | 2054.71 (1298.18 to 2954.56) | 96.62 (61.04 to 138.93) | 6080.36 (3893.29 to 8717.07) | 116.62 (74.67 to 167.19) | 0.39% (0.29 to 0.49) |
| Guinea-Bissau | 544.94 (350.65 to 778.8) | 136.23 (87.66 to 194.69) | 1123.24 (718.81 to 1618.11) | 142.93 (91.47 to 205.9) | 0.17% (0.15 to 0.18) |
| Guyana | 398.57 (260.06 to 575.96) | 148.28 (96.76 to 214.28) | 302.16 (196.76 to 432.69) | 146.54 (95.42 to 209.84) | 0.05% (0.01 to 0.1) |
| Haiti | 3452.07 (2263.08 to 5010.57) | 151.03 (99.01 to 219.21) | 6535.34 (4330.67 to 9333.83) | 161.85 (107.25 to 231.16) | 0.2% (0.17 to 0.23) |
| Honduras | 2641.71 (1740.94 to 3845.08) | 139.73 (92.08 to 203.38) | 4701.06 (3056.95 to 6685.99) | 144.43 (93.92 to 205.41) | 0.14% (0.13 to 0.15) |
| Hungary | 8.42 (3.6 to 15.43) | 0.37 (0.16 to 0.68) | 3.9 (1.6 to 7.31) | 0.27 (0.11 to 0.51) | -1.15% (-1.41 to -0.89) |
| Iceland | 0.13 (0.05 to 0.23) | 0.2 (0.08 to 0.37) | 0.15 (0.06 to 0.27) | 0.22 (0.09 to 0.4) | 0.29% (0.19 to 0.39) |
| India | 224263.6 (148096.3 to 320884.31) | 76.24 (50.34 to 109.08) | 322236.52 (210200.82 to 464939.05) | 82.79 (54 to 119.45) | 1.13% (0.77 to 1.49) |
| Indonesia | 13065.51 (8849.87 to 18406.28) | 20.06 (13.59 to 28.26) | 15029.56 (9756.9 to 21793.8) | 22 (14.28 to 31.9) | 0.31% (0.25 to 0.36) |
| Iran (Islamic Republic of) | 17924.96 (11853.26 to 25351.87) | 78.83 (52.13 to 111.5) | 15681.71 (10417.6 to 22123.92) | 79.6 (52.88 to 112.31) | 0.01% (-0.04 to 0.07) |
| Iraq | 5567.55 (3623.07 to 7880.78) | 77.8 (50.63 to 110.13) | 10586.58 (7035.58 to 15054.49) | 79.34 (52.73 to 112.82) | 0.03% (0.01 to 0.05) |
| Ireland | 2.22 (0.86 to 4.4) | 0.21 (0.08 to 0.42) | 1.99 (0.75 to 3.92) | 0.19 (0.07 to 0.38) | -0.3% (-0.34 to -0.26) |
| Israel | 3.69 (1.32 to 7.17) | 0.25 (0.09 to 0.48) | 5.25 (1.6 to 10.4) | 0.21 (0.07 to 0.42) | -0.61% (-0.66 to -0.56) |
| Italy | 70.5 (43.49 to 111.39) | 0.65 (0.4 to 1.02) | 11.57 (5.77 to 20.17) | 0.14 (0.07 to 0.24) | -6.3% (-7.36 to -5.22) |
| Jamaica | 1077.63 (707.86 to 1511.14) | 132.47 (87.01 to 185.76) | 907.47 (586.16 to 1305.03) | 140.87 (90.99 to 202.59) | 0.26% (0.23 to 0.29) |
| Japan | 114.7 (64.65 to 186.96) | 0.43 (0.24 to 0.7) | 28.1 (13.41 to 47.06) | 0.17 (0.08 to 0.28) | -4.61% (-5.49 to -3.72) |
| Jordan | 12.51 (6.44 to 20.85) | 0.84 (0.43 to 1.39) | 28.04 (13.6 to 51.4) | 0.73 (0.36 to 1.34) | -0.57% (-0.68 to -0.45) |
| Kazakhstan | 151.44 (100.01 to 209.57) | 3.18 (2.1 to 4.4) | 89.24 (49.24 to 140.14) | 1.87 (1.03 to 2.93) | -2.27% (-2.49 to -2.05) |
| Kenya | 17746.28 (11680.56 to 25568.41) | 186.83 (122.97 to 269.17) | 38093.92 (24888.22 to 55370.44) | 204.25 (133.45 to 296.89) | 0.28% (0.22 to 0.33) |
| Kiribati | 31.82 (20.84 to 44.67) | 127.91 (83.78 to 179.58) | 51.61 (33.99 to 72.91) | 131.83 (86.81 to 186.23) | 0.08% (0.06 to 0.09) |
| Kuwait | 4.48 (2.31 to 7.59) | 0.93 (0.48 to 1.58) | 5.85 (2.63 to 10.14) | 0.7 (0.32 to 1.22) | -1.01% (-1.14 to -0.89) |
| Kyrgyzstan | 1867.17 (1216.15 to 2653.56) | 127.14 (82.81 to 180.68) | 2540.69 (1664.01 to 3604.13) | 125.22 (82.01 to 177.64) | -0.09% (-0.15 to -0.02) |
| Lao People's Democratic Republic | 1718.66 (1104 to 2470.49) | 110.35 (70.88 to 158.62) | 2349.67 (1520.5 to 3373.34) | 108.68 (70.33 to 156.03) | 0.11% (-0.27 to 0.49) |
| Latvia | 5.6 (3.07 to 9.58) | 1.02 (0.56 to 1.74) | 1.89 (0.85 to 3.47) | 0.64 (0.29 to 1.18) | -2.86% (-3.89 to -1.82) |
| Lebanon | 9.87 (5.09 to 16.01) | 1.04 (0.54 to 1.69) | 10.03 (5.02 to 18.09) | 0.8 (0.4 to 1.44) | -1.03% (-1.26 to -0.8) |
| Lesotho | 1119.42 (725.46 to 1621.18) | 189.41 (122.75 to 274.3) | 1232.47 (806.42 to 1760.19) | 194.06 (126.97 to 277.15) | 0.07% (0.06 to 0.09) |
| Liberia | 1212.22 (799.79 to 1729.45) | 134.43 (88.69 to 191.79) | 2957.1 (1917.4 to 4267.4) | 146.13 (94.75 to 210.88) | 0.19% (0.15 to 0.22) |
| Libya | 1127.44 (753.56 to 1589.91) | 67.46 (45.09 to 95.13) | 1282.09 (863.69 to 1812.99) | 77.03 (51.89 to 108.92) | 0.44% (0.41 to 0.46) |
| Lithuania | 6.03 (2.96 to 10.63) | 0.74 (0.36 to 1.3) | 2.25 (0.95 to 4.16) | 0.56 (0.24 to 1.03) | -2.42% (-3.6 to -1.23) |
| Luxembourg | 0.2 (0.08 to 0.38) | 0.31 (0.13 to 0.58) | 0.3 (0.13 to 0.57) | 0.3 (0.13 to 0.56) | -0.47% (-1.06 to 0.12) |
| Madagascar | 9231.2 (6095.42 to 13193.96) | 201.98 (133.37 to 288.68) | 23568 (15250.07 to 33771.97) | 217.1 (140.48 to 311.1) | 0.22% (0.21 to 0.23) |
| Malawi | 7880.74 (5131.67 to 11371.84) | 214.47 (139.65 to 309.47) | 17993.5 (11793.18 to 26152.32) | 228.73 (149.91 to 332.44) | 0.2% (0.18 to 0.22) |
| Malaysia | 5134.61 (3326.83 to 7236.3) | 86.14 (55.81 to 121.4) | 6812.32 (4463.73 to 9541.23) | 87.37 (57.25 to 122.37) | 0.13% (0.09 to 0.17) |
| Maldives | 73.93 (48.8 to 104.34) | 85.4 (56.37 to 120.54) | 83.05 (54.03 to 117.56) | 84.27 (54.82 to 119.29) | -0.09% (-0.12 to -0.05) |
| Mali | 4831.5 (3156.19 to 7006.45) | 150.2 (98.12 to 217.82) | 14760.25 (9547.95 to 21263.71) | 152.91 (98.91 to 220.29) | 0.06% (0.04 to 0.09) |
| Malta | 0.14 (0.05 to 0.28) | 0.16 (0.06 to 0.32) | 0.1 (0.03 to 0.18) | 0.15 (0.05 to 0.3) | -0.34% (-0.58 to -0.1) |
| Marshall Islands | 24.13 (16 to 34.12) | 124.75 (82.69 to 176.36) | 22.16 (14.69 to 31.98) | 127.34 (84.44 to 183.81) | 0.06% (0.04 to 0.07) |
| Mauritania | 1025.12 (671.25 to 1469.11) | 134.36 (87.98 to 192.55) | 2275.62 (1471.34 to 3298.92) | 135.84 (87.83 to 196.93) | 0.05% (0.03 to 0.08) |
| Mauritius | 277.53 (179.49 to 393.16) | 85.3 (55.17 to 120.84) | 197.85 (128.46 to 284.54) | 85.06 (55.23 to 122.33) | -0.06% (-0.07 to -0.04) |
| Mexico | 19929.47 (13078.66 to 28671.68) | 63.13 (41.43 to 90.82) | 18731.15 (12318.78 to 26951) | 56.29 (37.02 to 81) | -0.44% (-0.46 to -0.41) |
| Micronesia (Federated States of) | 52.29 (34.37 to 73.75) | 124.97 (82.14 to 176.27) | 40.97 (27.13 to 57.91) | 128.14 (84.84 to 181.1) | 0.02% (0.01 to 0.04) |
| Monaco | 0.01 (0 to 0.01) | 0.2 (0.07 to 0.38) | 0.01 (0 to 0.02) | 0.19 (0.07 to 0.37) | -0.01% (-0.06 to 0.03) |
| Mongolia | 1101.94 (715.98 to 1599.54) | 138.51 (89.99 to 201.05) | 1217.27 (793.39 to 1727.6) | 132.02 (86.05 to 187.37) | -0.16% (-0.23 to -0.1) |
| Montenegro | 0.54 (0.26 to 1) | 0.33 (0.16 to 0.62) | 0.34 (0.15 to 0.65) | 0.3 (0.13 to 0.57) | -0.55% (-0.67 to -0.43) |
| Morocco | 7153.15 (4779.06 to 10041.35) | 80.2 (53.58 to 112.58) | 7837.63 (5046.2 to 11055.95) | 81.09 (52.21 to 114.39) | 0.03% (0 to 0.06) |
| Mozambique | 11250.8 (7343.93 to 15880.13) | 217.97 (142.28 to 307.65) | 27971.38 (18163.44 to 40935.77) | 222.44 (144.44 to 325.53) | 0.02% (-0.11 to 0.15) |
| Myanmar | 15170.83 (9922.43 to 21165.35) | 107.83 (70.53 to 150.44) | 16422.28 (10661.51 to 23480.34) | 105.67 (68.6 to 151.08) | -0.08% (-0.11 to -0.06) |
| Namibia | 996.02 (648.68 to 1431.7) | 186.06 (121.18 to 267.45) | 1460.08 (957.75 to 2120.31) | 184.14 (120.79 to 267.4) | -0.05% (-0.06 to -0.03) |
| Nauru | 0.15 (0.09 to 0.23) | 4.31 (2.56 to 6.37) | 0.16 (0.1 to 0.24) | 4.3 (2.58 to 6.32) | -0.01% (-0.17 to 0.15) |
| Nepal | 4413.06 (3059.29 to 6194.99) | 62.55 (43.36 to 87.8) | 6957.13 (4491.57 to 9851.63) | 74.42 (48.05 to 105.39) | 0.75% (0.64 to 0.86) |
| Netherlands | 5.53 (2.11 to 10.77) | 0.19 (0.07 to 0.37) | 5.53 (2.06 to 10.15) | 0.2 (0.07 to 0.36) | 0.04% (-0.03 to 0.11) |
| New Zealand | 10.68 (6.72 to 16.2) | 1.3 (0.82 to 1.97) | 9.38 (6.42 to 13.46) | 0.95 (0.65 to 1.36) | -0.38% (-0.87 to 0.1) |
| Nicaragua | 2218.61 (1450.69 to 3185.62) | 139.82 (91.42 to 200.76) | 2814.22 (1857.01 to 4000.57) | 144.41 (95.29 to 205.29) | 0.04% (-0.02 to 0.11) |
| Niger | 4226.74 (2698.89 to 6039.35) | 133.56 (85.28 to 190.84) | 14741.98 (9614.22 to 21186.36) | 140.53 (91.65 to 201.96) | 0.15% (0.13 to 0.17) |
| Nigeria | 47795.89 (30916.27 to 68865.52) | 146.51 (94.77 to 211.09) | 144802.22 (94674.9 to 208299.79) | 159.73 (104.43 to 229.77) | 0.29% (0.27 to 0.31) |
| Niue | 0.02 (0.01 to 0.03) | 2.86 (1.68 to 4.34) | 0.01 (0.01 to 0.01) | 2.41 (1.44 to 3.65) | -0.55% (-0.63 to -0.46) |
| North Macedonia | 2.49 (1.16 to 4.4) | 0.47 (0.22 to 0.84) | 1.07 (0.47 to 1.97) | 0.31 (0.14 to 0.57) | -1.98% (-2.19 to -1.78) |
| Northern Mariana Islands | 12.16 (8.2 to 16.78) | 106.1 (71.54 to 146.36) | 13.59 (9.14 to 18.98) | 115.26 (77.54 to 160.97) | 0.17% (0.12 to 0.23) |
| Norway | 1.5 (0.77 to 2.41) | 0.18 (0.09 to 0.29) | 1.66 (0.89 to 2.67) | 0.17 (0.09 to 0.28) | -1.4% (-2.39 to -0.39) |
| Oman | 5.06 (2.33 to 9.24) | 0.75 (0.34 to 1.37) | 8.43 (3.88 to 15.36) | 0.79 (0.36 to 1.44) | 0.3% (0.14 to 0.46) |
| Pakistan | 41724.87 (27223.61 to 59130.19) | 98.17 (64.05 to 139.12) | 89178.39 (59535.46 to 126671.46) | 112.18 (74.89 to 159.34) | 0.31% (0 to 0.63) |
| Palau | 0.15 (0.09 to 0.22) | 3.16 (1.86 to 4.68) | 0.11 (0.06 to 0.16) | 3.04 (1.83 to 4.57) | -0.11% (-0.18 to -0.05) |
| Palestine | 606.63 (395.29 to 863.09) | 75.49 (49.19 to 107.4) | 1355.64 (901.84 to 1906.55) | 75.52 (50.24 to 106.21) | 0.06% (0.03 to 0.09) |
| Panama | 1054.89 (704.85 to 1485.69) | 131.62 (87.94 to 185.37) | 1454.08 (970.36 to 2046.69) | 128 (85.42 to 180.17) | -0.09% (-0.1 to -0.07) |
| Papua New Guinea | 1781.78 (1194.76 to 2499.01) | 120.31 (80.68 to 168.74) | 4237.44 (2848.22 to 5967.3) | 125.45 (84.32 to 176.67) | 0.11% (0.09 to 0.14) |
| Paraguay | 2034.99 (1334.35 to 2889.04) | 140.14 (91.89 to 198.96) | 2864.48 (1883.11 to 4048.48) | 141.76 (93.2 to 200.36) | 0.04% (0.02 to 0.06) |
| Peru | 10233.89 (6780.79 to 14537.83) | 132.5 (87.79 to 188.22) | 12457.13 (8244.71 to 17983.7) | 135.26 (89.52 to 195.26) | 0.07% (0.06 to 0.08) |
| Philippines | 15598.3 (10173.48 to 22301.15) | 68.41 (44.62 to 97.81) | 26638.81 (17195.47 to 38557.23) | 79.37 (51.23 to 114.88) | 0.88% (0.69 to 1.07) |
| Poland | 67.69 (42.13 to 106.52) | 0.71 (0.44 to 1.12) | 13.8 (6.83 to 23.16) | 0.24 (0.12 to 0.4) | -3.95% (-4.21 to -3.68) |
| Portugal | 9.05 (3.92 to 16.85) | 0.38 (0.16 to 0.7) | 3.73 (1.56 to 7.29) | 0.25 (0.1 to 0.49) | -1.65% (-1.87 to -1.43) |
| Puerto Rico | 7.88 (4.09 to 13.67) | 0.78 (0.4 to 1.35) | 3.84 (1.82 to 6.66) | 0.7 (0.33 to 1.22) | -0.38% (-0.52 to -0.23) |
| Qatar | 1.3 (0.68 to 2.13) | 1.27 (0.67 to 2.09) | 3.29 (1.51 to 6.04) | 0.8 (0.37 to 1.46) | -1.98% (-2.13 to -1.82) |
| Republic of Korea | 36.66 (16.15 to 72.05) | 0.29 (0.13 to 0.57) | 16.79 (6.01 to 33.13) | 0.25 (0.09 to 0.48) | -0.76% (-0.85 to -0.67) |
| Republic of Moldova | 20.42 (12.12 to 30.98) | 1.78 (1.05 to 2.69) | 3.51 (1.61 to 6.22) | 0.65 (0.3 to 1.15) | -3.89% (-4.12 to -3.67) |
| Romania | 43.64 (22.28 to 74.01) | 0.76 (0.39 to 1.29) | 13.86 (5.95 to 24.98) | 0.45 (0.19 to 0.81) | -3.78% (-5.24 to -2.3) |
| Russian Federation | 972.23 (710.48 to 1301.38) | 2.9 (2.12 to 3.88) | 746.42 (511.84 to 1026.34) | 2.85 (1.96 to 3.92) | -0.35% (-0.47 to -0.22) |
| Rwanda | 5767.57 (3766.91 to 8100.18) | 206.66 (134.98 to 290.24) | 9965.04 (6489.61 to 14606.45) | 212.49 (138.38 to 311.46) | 0.07% (0.04 to 0.1) |
| Saint Kitts and Nevis | 0.28 (0.17 to 0.41) | 2.01 (1.28 to 2.97) | 0.1 (0.05 to 0.16) | 0.88 (0.45 to 1.44) | -3.27% (-3.55 to -2.98) |
| Saint Lucia | 70.68 (46.7 to 100.66) | 143.9 (95.07 to 204.94) | 48.42 (32.14 to 69.58) | 146.17 (97.02 to 210.05) | 0.07% (0.06 to 0.08) |
| Saint Vincent and the Grenadines | 58.98 (38.8 to 85.11) | 143.93 (94.7 to 207.71) | 38.1 (25.09 to 54.97) | 143.71 (94.65 to 207.36) | -0.01% (-0.02 to 0) |
| Samoa | 82.02 (54.78 to 116.26) | 124.07 (82.87 to 175.88) | 92.79 (60.93 to 131.81) | 129.41 (84.98 to 183.84) | 0.07% (0.04 to 0.09) |
| San Marino | 0.01 (0 to 0.02) | 0.2 (0.07 to 0.39) | 0.01 (0 to 0.02) | 0.2 (0.07 to 0.39) | -0.04% (-0.07 to 0) |
| Sao Tome and Principe | 68.42 (44.91 to 97.78) | 138.5 (90.9 to 197.91) | 111.63 (73.42 to 158.59) | 145.23 (95.52 to 206.34) | 0.12% (0.1 to 0.14) |
| Saudi Arabia | 69.22 (37.97 to 117.22) | 1.19 (0.66 to 2.02) | 61.57 (31.07 to 106.83) | 0.8 (0.4 to 1.39) | -1.54% (-1.62 to -1.46) |
| Senegal | 4189.42 (2742.78 to 6019.87) | 140.5 (91.98 to 201.88) | 8392.18 (5434.41 to 12198.27) | 143.61 (92.99 to 208.74) | -0.1% (-0.19 to -0.01) |
| Serbia | 5.84 (2.26 to 10.85) | 0.26 (0.1 to 0.49) | 4.34 (1.63 to 8.49) | 0.29 (0.11 to 0.56) | -1.66% (-3.25 to -0.05) |
| Seychelles | 20.55 (13.66 to 28.99) | 88.64 (58.9 to 125.04) | 19.27 (12.84 to 27.23) | 85.07 (56.67 to 120.21) | -0.12% (-0.13 to -0.11) |
| Sierra Leone | 1991.42 (1318.87 to 2798.73) | 139.07 (92.11 to 195.45) | 4744.07 (3033.27 to 6949.18) | 147.9 (94.56 to 216.64) | 0.22% (0.2 to 0.23) |
| Singapore | 3.58 (1.54 to 6.51) | 0.5 (0.22 to 0.91) | 1.94 (0.74 to 3.83) | 0.26 (0.1 to 0.51) | -2.91% (-3.27 to -2.56) |
| Slovakia | 5.69 (2.48 to 11) | 0.42 (0.18 to 0.82) | 3.52 (1.52 to 6.49) | 0.42 (0.18 to 0.78) | 0.41% (-0.93 to 1.76) |
| Slovenia | 0.96 (0.39 to 1.93) | 0.22 (0.09 to 0.44) | 0.66 (0.26 to 1.28) | 0.22 (0.09 to 0.42) | -0.74% (-1.6 to 0.13) |
| Solomon Islands | 168.6 (110.73 to 236.52) | 124.92 (82.05 to 175.25) | 302.75 (200.67 to 434.6) | 128.61 (85.24 to 184.62) | 0.06% (0.05 to 0.08) |
| Somalia | 6669.2 (4361.57 to 9411.49) | 209.24 (136.84 to 295.28) | 19251.81 (12485 to 27518.92) | 222.43 (144.25 to 317.94) | 0.17% (0.14 to 0.19) |
| South Africa | 26974.3 (17622.71 to 38603.06) | 211.67 (138.29 to 302.93) | 31901.39 (20871.04 to 46467.73) | 213.29 (139.54 to 310.68) | -0.02% (-0.04 to 0) |
| South Sudan | 4472.1 (2923.11 to 6425.58) | 197.16 (128.87 to 283.28) | 8461.42 (5471.31 to 12373.31) | 215.58 (139.4 to 315.24) | 0.27% (0.23 to 0.31) |
| Spain | 28.96 (12.83 to 53.39) | 0.32 (0.14 to 0.59) | 14.25 (5.71 to 27.37) | 0.2 (0.08 to 0.39) | -2.13% (-2.43 to -1.83) |
| Sri Lanka | 51.16 (25.85 to 86.34) | 0.93 (0.47 to 1.58) | 48.58 (23.51 to 86.62) | 0.91 (0.44 to 1.62) | 0.04% (-0.18 to 0.26) |
| Sudan | 5066.06 (3326.35 to 7175.76) | 66.96 (43.97 to 94.85) | 11040.84 (7315.3 to 15794.45) | 70.16 (46.49 to 100.37) | 0.14% (0.13 to 0.16) |
| Suriname | 173.75 (114.14 to 246.85) | 136.61 (89.74 to 194.08) | 197.3 (128.67 to 281.83) | 135.78 (88.54 to 193.95) | -0.04% (-0.06 to -0.02) |
| Sweden | 2.92 (1.47 to 4.74) | 0.19 (0.1 to 0.31) | 2.63 (1.14 to 4.14) | 0.14 (0.06 to 0.23) | -0.95% (-1.66 to -0.23) |
| Switzerland | 3.25 (1.43 to 6.28) | 0.28 (0.12 to 0.53) | 3.49 (1.23 to 6.6) | 0.26 (0.09 to 0.5) | -0.85% (-1.73 to 0.05) |
| Syrian Arab Republic | 3857.22 (2520.37 to 5470.57) | 74.25 (48.52 to 105.3) | 3528.46 (2282.88 to 5114.78) | 79.56 (51.47 to 115.33) | 0.12% (0.07 to 0.18) |
| Taiwan (Province of China) | 32.97 (15.07 to 57.86) | 0.58 (0.26 to 1.01) | 25.68 (13.95 to 41.83) | 0.81 (0.44 to 1.33) | 1.67% (1.23 to 2.11) |
| Tajikistan | 2546.5 (1676.07 to 3635.46) | 132.02 (86.89 to 188.47) | 4146.42 (2716.3 to 5922.36) | 133.04 (87.15 to 190.02) | 0.05% (-0.02 to 0.12) |
| Thailand | 15124.9 (9971.1 to 21609.03) | 86.2 (56.83 to 123.15) | 8982.37 (5840.52 to 12790.66) | 83.01 (53.97 to 118.2) | -0.1% (-0.12 to -0.09) |
| Timor-Leste | 245.62 (160.18 to 347.5) | 92.13 (60.08 to 130.34) | 467.74 (297.63 to 674.91) | 93.44 (59.46 to 134.83) | 0.02% (-0.01 to 0.04) |
| Togo | 2012.32 (1323.5 to 2886.73) | 137.23 (90.25 to 196.86) | 4252.06 (2721.77 to 5970.28) | 141.6 (90.64 to 198.82) | 0.07% (0.05 to 0.09) |
| Tokelau | 0.02 (0.01 to 0.03) | 2.98 (1.72 to 4.45) | 0.01 (0.01 to 0.02) | 2.42 (1.3 to 3.74) | -0.7% (-0.81 to -0.6) |
| Tonga | 62.92 (42.08 to 88.97) | 165.19 (110.47 to 233.59) | 60.08 (40.11 to 84.61) | 170.81 (114.03 to 240.54) | -0.03% (-0.25 to 0.2) |
| Trinidad and Tobago | 516.23 (337.9 to 727.86) | 133.69 (87.5 to 188.49) | 388.44 (256.85 to 553.03) | 137.03 (90.61 to 195.09) | 0% (-0.05 to 0.06) |
| Tunisia | 25.24 (11.54 to 44.02) | 0.86 (0.39 to 1.51) | 20.69 (9.64 to 36.55) | 0.77 (0.36 to 1.36) | -0.43% (-0.54 to -0.31) |
| Türkiye | 144.36 (63.98 to 261.91) | 0.73 (0.32 to 1.33) | 141.54 (69.86 to 254.58) | 0.74 (0.36 to 1.33) | 0% (-0.09 to 0.08) |
| Turkmenistan | 1672.39 (1108.09 to 2371.78) | 128.68 (85.26 to 182.49) | 1800.65 (1178 to 2579.95) | 126.73 (82.91 to 181.58) | -0.08% (-0.11 to -0.05) |
| Tuvalu | 0.12 (0.07 to 0.17) | 4.2 (2.49 to 6.18) | 0.12 (0.07 to 0.19) | 3.43 (2.06 to 5.28) | -0.65% (-0.71 to -0.58) |
| Uganda | 14018.69 (9239.95 to 19725.75) | 209.04 (137.78 to 294.14) | 36399.26 (23975.8 to 53231.21) | 206.51 (136.03 to 302.01) | 0.18% (-0.07 to 0.44) |
| Ukraine | 78.52 (45.36 to 126.09) | 0.7 (0.4 to 1.12) | 48.67 (27.7 to 78.92) | 0.71 (0.4 to 1.15) | 0.27% (0.1 to 0.44) |
| United Arab Emirates | 323.91 (213.98 to 450.67) | 67.61 (44.66 to 94.07) | 862.22 (568.61 to 1206.15) | 68.3 (45.04 to 95.54) | 0.07% (0.05 to 0.09) |
| United Kingdom | 31.83 (17.79 to 51.98) | 0.29 (0.16 to 0.47) | 31.7 (17.01 to 50.81) | 0.26 (0.14 to 0.42) | -0.36% (-0.4 to -0.31) |
| United Republic of Tanzania | 20619.59 (13416.86 to 29119.94) | 203.85 (132.64 to 287.88) | 48178.02 (31074.62 to 69429.37) | 218.87 (141.17 to 315.41) | 0.24% (0.22 to 0.27) |
| United States of America | 270.82 (161.46 to 427.4) | 0.5 (0.3 to 0.79) | 299.54 (190.65 to 448.34) | 0.48 (0.3 to 0.71) | 0.3% (-0.13 to 0.72) |
| United States Virgin Islands | 0.37 (0.21 to 0.6) | 1.21 (0.69 to 1.94) | 0.11 (0.06 to 0.2) | 0.78 (0.4 to 1.37) | -2.15% (-2.39 to -1.9) |
| Uruguay | 6.35 (3.25 to 10.75) | 0.79 (0.4 to 1.34) | 5.5 (2.76 to 9.7) | 0.78 (0.39 to 1.37) | -0.08% (-0.19 to 0.03) |
| Uzbekistan | 9187.9 (6034.13 to 13097.28) | 125.81 (82.63 to 179.35) | 11556.21 (7444.24 to 16504.07) | 131.7 (84.84 to 188.08) | 0.24% (0.19 to 0.28) |
| Vanuatu | 77.31 (52 to 108.31) | 137.06 (92.19 to 192.01) | 148.71 (97.78 to 207.75) | 141.24 (92.87 to 197.32) | 0.06% (0.05 to 0.08) |
| Venezuela (Bolivarian Republic of) | 80.61 (44.93 to 129.7) | 1.23 (0.69 to 1.98) | 59.19 (31.05 to 98.52) | 0.9 (0.47 to 1.5) | -1.35% (-1.64 to -1.06) |
| Viet Nam | 325.97 (183.37 to 526.99) | 1.34 (0.76 to 2.17) | 242.06 (132.69 to 397.57) | 1.03 (0.57 to 1.69) | -1.16% (-1.35 to -0.97) |
| Yemen | 4622.52 (3165.88 to 6520.06) | 82.08 (56.22 to 115.78) | 11967.52 (7809.49 to 17111.59) | 93.68 (61.13 to 133.95) | 0.28% (-0.32 to 0.88) |
| Zambia | 7257.62 (4819.45 to 10122.61) | 227.42 (151.02 to 317.19) | 16571.16 (10951.29 to 23850.31) | 220.09 (145.45 to 316.77) | -0.1% (-0.16 to -0.04) |
| Zimbabwe | 7730.72 (5022.86 to 10989.32) | 181.52 (117.94 to 258.04) | 10987.07 (7270.43 to 15693.36) | 190.27 (125.91 to 271.77) | 0.17% (0.12 to 0.21) |

Table S15. Prevalence of Rheumatic heart disease in 1990 and 2021 for both sexes in 204 countries, with EAPC from 1990 and 2021.

| location | Num_1990 | ASR_1990 | Num_2021 | ASR_2021 | EAPC_CI |
| --- | --- | --- | --- | --- | --- |
| Afghanistan | 18632.18 (12334.56 to 26140.29) | 482.85 (319.65 to 677.42) | 57345.8 (38057.58 to 80316.14) | 466.4 (309.53 to 653.23) | 0.08% (-0.06 to 0.22) |
| Albania | 9242.69 (6306.02 to 12664.17) | 886.89 (605.1 to 1215.2) | 4263.62 (2923.01 to 5902.42) | 898.52 (616 to 1243.88) | 0.25% (0.18 to 0.32) |
| Algeria | 29609.34 (21105.09 to 41118.28) | 302.74 (215.79 to 420.41) | 46461.66 (31552.49 to 63486) | 395.61 (268.67 to 540.58) | 0.91% (0.52 to 1.3) |
| American Samoa | 170.23 (123.65 to 225.14) | 1044.07 (758.33 to 1380.81) | 172.69 (123.39 to 231.68) | 1119.96 (800.2 to 1502.52) | 0.19% (0.18 to 0.21) |
| Andorra | 0.55 (0.35 to 0.78) | 5.09 (3.27 to 7.21) | 0.56 (0.36 to 0.82) | 4.76 (3.03 to 6.88) | -0.3% (-0.37 to -0.24) |
| Angola | 53439.45 (36407.38 to 74629.79) | 1401.19 (954.61 to 1956.81) | 182134.3 (122540.36 to 254208.64) | 1396.47 (939.55 to 1949.08) | -0.08% (-0.1 to -0.05) |
| Antigua and Barbuda | 168.33 (113.96 to 233.04) | 944.13 (639.19 to 1307.08) | 179.52 (120.91 to 249.1) | 992.27 (668.3 to 1376.89) | 0.24% (0.19 to 0.28) |
| Argentina | 76614.16 (52833.34 to 104206.28) | 798.9 (550.92 to 1086.61) | 90136.04 (61512.28 to 124803.18) | 841.2 (574.06 to 1164.73) | 0.2% (0.16 to 0.25) |
| Armenia | 8652.46 (5951.29 to 11812.69) | 915.71 (629.84 to 1250.16) | 5048.62 (3468.05 to 6832.69) | 875.11 (601.14 to 1184.36) | -0.17% (-0.35 to 0.02) |
| Australia | 693.36 (449.97 to 994.69) | 17.74 (11.51 to 25.44) | 792.78 (529.19 to 1139.04) | 16.74 (11.18 to 24.06) | -0.18% (-0.26 to -0.11) |
| Austria | 149.92 (111.78 to 193.67) | 10.51 (7.84 to 13.58) | 53.69 (38.91 to 71.21) | 4.06 (2.94 to 5.38) | -3.75% (-4.07 to -3.42) |
| Azerbaijan | 18871.89 (12826.82 to 26078.76) | 847.66 (576.14 to 1171.37) | 18857.02 (12810.22 to 26270.19) | 810.8 (550.8 to 1129.54) | -0.01% (-0.15 to 0.13) |
| Bahamas | 775.93 (520.51 to 1055.94) | 944.43 (633.55 to 1285.25) | 957.35 (649.11 to 1322.12) | 1016.18 (689 to 1403.36) | 0.18% (0.12 to 0.23) |
| Bahrain | 26.04 (18.44 to 36.06) | 18.78 (13.3 to 26) | 52.2 (35.73 to 74.33) | 16.85 (11.53 to 23.99) | -0.61% (-0.69 to -0.53) |
| Bangladesh | 151211.02 (104843.38 to 206088.05) | 364.99 (253.07 to 497.46) | 210649.04 (140098.42 to 293157.52) | 451.79 (300.48 to 628.76) | 0.7% (0.6 to 0.81) |
| Barbados | 626.3 (424.98 to 852.33) | 958.8 (650.6 to 1304.82) | 536.5 (360.88 to 747.94) | 1016.8 (683.95 to 1417.53) | 0.18% (0.13 to 0.24) |
| Belarus | 316.07 (205.43 to 479.95) | 13.55 (8.81 to 20.58) | 181.37 (115.18 to 278) | 11.64 (7.39 to 17.84) | -0.54% (-0.59 to -0.49) |
| Belgium | 107.17 (69.33 to 154.9) | 5.71 (3.7 to 8.26) | 110.74 (70.06 to 163.74) | 5.67 (3.59 to 8.39) | -0.94% (-2.27 to 0.4) |
| Belize | 643.41 (437.83 to 897.56) | 880.97 (599.49 to 1228.95) | 1270.93 (848.96 to 1758.39) | 970.79 (648.47 to 1343.13) | 0.29% (0.27 to 0.32) |
| Benin | 14910.75 (9757.78 to 20753.28) | 791.87 (518.21 to 1102.15) | 44998.97 (30365.67 to 63527.71) | 869.72 (586.9 to 1227.84) | 0.3% (0.28 to 0.31) |
| Bermuda | 1.87 (1.2 to 2.7) | 16.08 (10.38 to 23.23) | 1.14 (0.76 to 1.68) | 12.72 (8.55 to 18.82) | -1.01% (-1.13 to -0.89) |
| Bhutan | 1080 (722.54 to 1490.82) | 440.55 (294.74 to 608.13) | 898.91 (589.37 to 1240.64) | 463.49 (303.88 to 639.68) | 0.23% (0.19 to 0.27) |
| Bolivia (Plurinational State of) | 19875.82 (13152.48 to 27657.39) | 849.79 (562.33 to 1182.49) | 29726.7 (19841.72 to 41665.23) | 886.68 (591.83 to 1242.77) | 0.2% (0.16 to 0.23) |
| Bosnia and Herzegovina | 65.5 (39.71 to 98.89) | 5.8 (3.51 to 8.75) | 30.69 (18.64 to 45.91) | 5.97 (3.63 to 8.93) | 0.13% (0.06 to 0.19) |
| Botswana | 6327.95 (4265.46 to 8928.15) | 1199.72 (808.69 to 1692.7) | 8437.36 (5724.6 to 11701.36) | 1238.04 (839.99 to 1716.98) | 0.06% (0.02 to 0.11) |
| Brazil | 519406.14 (353020.67 to 715474.06) | 1023.86 (695.88 to 1410.34) | 500737.6 (340290.82 to 691901.37) | 1057.32 (718.53 to 1460.96) | 0.1% (0.05 to 0.15) |
| Brunei Darussalam | 10.25 (6.97 to 14.49) | 12.73 (8.65 to 17.99) | 9.18 (6.21 to 13.44) | 9.34 (6.32 to 13.68) | -1.32% (-1.42 to -1.23) |
| Bulgaria | 158.9 (99.73 to 241.38) | 8.7 (5.46 to 13.22) | 62.86 (40.16 to 94.35) | 6.34 (4.05 to 9.51) | -1.29% (-1.45 to -1.13) |
| Burkina Faso | 29858.43 (20005.18 to 41521.47) | 789.76 (529.14 to 1098.25) | 72571.56 (48253.18 to 100814.58) | 834.22 (554.68 to 1158.88) | 0.13% (0.11 to 0.14) |
| Burundi | 24209.26 (16260.47 to 33448.24) | 1163.89 (781.74 to 1608.07) | 64660.66 (43082.78 to 89151.04) | 1264.35 (842.42 to 1743.22) | 0.27% (0.21 to 0.33) |
| Cabo Verde | 1245.85 (846.26 to 1736.15) | 923.09 (627.02 to 1286.37) | 1487.69 (1003.48 to 2048.29) | 1000.55 (674.9 to 1377.59) | 0.33% (0.26 to 0.4) |
| Cambodia | 20585.79 (13953.58 to 28412.87) | 532.16 (360.71 to 734.49) | 26421.94 (17661.94 to 36639.58) | 541.15 (361.73 to 750.42) | -0.06% (-0.23 to 0.11) |
| Cameroon | 31170.74 (21469.89 to 43463.03) | 793.01 (546.22 to 1105.74) | 106223.78 (70064.89 to 148929.84) | 882.33 (581.98 to 1237.05) | 0.27% (0.2 to 0.35) |
| Canada | 723.6 (477.67 to 1098.18) | 12.61 (8.32 to 19.13) | 751.58 (491.48 to 1111.13) | 11.88 (7.77 to 17.56) | -0.17% (-0.2 to -0.13) |
| Central African Republic | 14007.06 (9250.71 to 19797.91) | 1409.95 (931.17 to 1992.85) | 30402.26 (20108.57 to 42699.62) | 1479.31 (978.44 to 2077.68) | 0.16% (0.15 to 0.18) |
| Chad | 18421.61 (12217.8 to 25979.22) | 807.13 (535.31 to 1138.26) | 60234.78 (40566.43 to 83605.63) | 823.55 (554.64 to 1143.08) | 0.05% (0.02 to 0.07) |
| Chile | 527.03 (344.22 to 794.61) | 13.73 (8.97 to 20.71) | 451.82 (281.67 to 699.69) | 11.87 (7.4 to 18.39) | -0.6% (-0.67 to -0.53) |
| China | 2293147.32 (1585270.65 to 3141821.61) | 688.15 (475.72 to 942.82) | 1371396.36 (943285.61 to 1891961.89) | 534.38 (367.56 to 737.22) | -0.01% (-0.33 to 0.32) |
| Colombia | 1730.8 (1122.61 to 2485.26) | 15.96 (10.35 to 22.91) | 1631.12 (1045.12 to 2429.14) | 14.67 (9.4 to 21.85) | -0.32% (-0.47 to -0.18) |
| Comoros | 2272.96 (1547.44 to 3145.33) | 1249.57 (850.71 to 1729.15) | 3113.65 (2075.56 to 4373.91) | 1338.39 (892.17 to 1880.11) | 0.26% (0.24 to 0.28) |
| Congo | 13846.82 (9286.88 to 19410.36) | 1485.4 (996.24 to 2082.23) | 28131.76 (18886.99 to 39323.35) | 1517.35 (1018.72 to 2121) | -0.55% (-0.84 to -0.25) |
| Cook Islands | 3.24 (2.5 to 4.17) | 50.89 (39.22 to 65.42) | 2.05 (1.56 to 2.68) | 50.57 (38.44 to 66.13) | -0.16% (-0.3 to -0.02) |
| Costa Rica | 9537.61 (6535.07 to 13225.3) | 942.28 (645.64 to 1306.61) | 10631.77 (7221.28 to 14544.6) | 1002.36 (680.82 to 1371.26) | 0.26% (0.2 to 0.32) |
| Côte d'Ivoire | 38637.06 (25753.92 to 53794.09) | 838.47 (558.89 to 1167.39) | 88367.48 (59327.98 to 123293.9) | 881.01 (591.49 to 1229.22) | 0.09% (0.04 to 0.13) |
| Croatia | 44.96 (27.66 to 66.43) | 4.39 (2.7 to 6.48) | 21.13 (14 to 30.46) | 3.39 (2.25 to 4.89) | -1.55% (-2.28 to -0.83) |
| Cuba | 28302.86 (19184.84 to 38585.35) | 1026.6 (695.87 to 1399.56) | 17804.49 (12017.42 to 24615.26) | 958.92 (647.24 to 1325.73) | 0% (-0.08 to 0.08) |
| Cyprus | 9.83 (6.21 to 13.9) | 5.04 (3.18 to 7.12) | 7.97 (4.81 to 12.06) | 3.79 (2.29 to 5.74) | -1.35% (-1.7 to -1.01) |
| Czechia | 144.85 (87.41 to 224.21) | 6.05 (3.65 to 9.36) | 102.14 (63.33 to 155.08) | 6.16 (3.82 to 9.35) | -1.54% (-2.78 to -0.29) |
| Democratic People's Republic of Korea | 35777.76 (24774.46 to 49069.68) | 656.11 (454.32 to 899.86) | 31750.02 (21412.25 to 44231.34) | 623.87 (420.74 to 869.13) | -0.12% (-0.15 to -0.1) |
| Democratic Republic of the Congo | 205755.76 (139624.36 to 285986.04) | 1433.38 (972.68 to 1992.3) | 505905.02 (330553.94 to 714096.65) | 1472.95 (962.42 to 2079.11) | 0.06% (0.02 to 0.09) |
| Denmark | 34.45 (20.74 to 53.11) | 3.59 (2.16 to 5.53) | 31.65 (18.68 to 48.44) | 3.22 (1.9 to 4.93) | -0.29% (-0.91 to 0.34) |
| Djibouti | 2017.82 (1377.35 to 2787.1) | 1263.94 (862.76 to 1745.82) | 4983.35 (3376.58 to 6922.78) | 1302.24 (882.36 to 1809.05) | 0.12% (0.08 to 0.16) |
| Dominica | 230.8 (154.08 to 321.45) | 957.22 (639.02 to 1333.18) | 159.87 (108.85 to 221.73) | 1002.49 (682.57 to 1390.4) | 0.17% (0.14 to 0.2) |
| Dominican Republic | 22672.89 (15537.5 to 31979.34) | 904.64 (619.94 to 1275.96) | 26350.71 (17954.3 to 36262.29) | 922.14 (628.31 to 1268.99) | 0.15% (0.12 to 0.19) |
| Ecuador | 30963.82 (20640.41 to 43625.61) | 857.26 (571.45 to 1207.81) | 43711.01 (29203.57 to 61778.31) | 882.13 (589.36 to 1246.75) | 0.14% (0.11 to 0.17) |
| Egypt | 106829.92 (72289.56 to 146605.72) | 550.96 (372.82 to 756.09) | 182453.45 (121568.25 to 254926.29) | 544.43 (362.75 to 760.68) | 0.17% (0.05 to 0.28) |
| El Salvador | 17191.65 (11413.06 to 23883.5) | 865.94 (574.87 to 1203.01) | 15763.79 (10659.28 to 21922.85) | 884.94 (598.38 to 1230.69) | 0.19% (0.11 to 0.27) |
| Equatorial Guinea | 2255.99 (1517.45 to 3185.22) | 1430.38 (962.12 to 2019.54) | 8708.78 (5915.35 to 12144.4) | 1494.32 (1015 to 2083.83) | 0.09% (0.05 to 0.13) |
| Eritrea | 19271.66 (12518.67 to 26716.7) | 1434.66 (931.94 to 1988.89) | 34626.02 (22750.35 to 48922.33) | 1506.39 (989.74 to 2128.34) | 0.31% (0.08 to 0.55) |
| Estonia | 42.24 (26.91 to 63.67) | 12.47 (7.94 to 18.8) | 23.91 (15.24 to 35.67) | 11.32 (7.22 to 16.9) | -0.34% (-0.44 to -0.25) |
| Eswatini | 3820.43 (2557.26 to 5291.91) | 1151.73 (770.93 to 1595.33) | 4782.09 (3184.88 to 6781.35) | 1211.91 (807.14 to 1718.58) | 0.17% (0.13 to 0.21) |
| Ethiopia | 192883.11 (129801.48 to 267070.42) | 972.79 (654.64 to 1346.94) | 541621.89 (362954.46 to 761661.51) | 1313.57 (880.25 to 1847.22) | 1.18% (1.04 to 1.32) |
| Fiji | 1564.91 (883.65 to 2132.06) | 591.72 (334.13 to 806.18) | 2120.9 (1509.46 to 2832.91) | 817.04 (581.49 to 1091.33) | 1.83% (1.13 to 2.53) |
| Finland | 34.71 (20.82 to 52.08) | 3.63 (2.18 to 5.45) | 28.9 (17.53 to 44.26) | 3.19 (1.93 to 4.89) | -0.56% (-1.25 to 0.14) |
| France | 621.5 (392.38 to 906.23) | 5.07 (3.2 to 7.4) | 566.25 (344.34 to 813.08) | 4.63 (2.82 to 6.65) | -0.35% (-0.38 to -0.31) |
| Gabon | 4901.73 (3397.15 to 6815.68) | 1392.57 (965.13 to 1936.32) | 8962.81 (6068.56 to 12378.66) | 1460.91 (989.15 to 2017.68) | 0.16% (0.14 to 0.17) |
| Gambia | 3175.56 (2155.26 to 4396.08) | 844.34 (573.05 to 1168.86) | 8470.15 (5706.34 to 11783.17) | 931.78 (627.74 to 1296.24) | 0.26% (0.23 to 0.28) |
| Georgia | 12108.8 (8163.24 to 16787.49) | 911.63 (614.58 to 1263.88) | 5854.93 (4008.03 to 8186.88) | 853.49 (584.26 to 1193.42) | -0.1% (-0.21 to 0) |
| Germany | 693.42 (429.23 to 1016.96) | 5.4 (3.34 to 7.91) | 552.98 (338.28 to 812.53) | 4.65 (2.84 to 6.83) | -2.13% (-3.4 to -0.85) |
| Ghana | 47738 (32360.47 to 66238.47) | 848.59 (575.24 to 1177.45) | 102502.33 (68676.87 to 143470.58) | 878.05 (588.29 to 1228.99) | 0.12% (0.09 to 0.15) |
| Greece | 103.12 (64.35 to 148.45) | 4.57 (2.85 to 6.58) | 55.24 (35 to 79.53) | 3.73 (2.36 to 5.36) | -0.93% (-1.05 to -0.82) |
| Greenland | 1.62 (1.04 to 2.46) | 13.1 (8.39 to 19.87) | 1.34 (0.85 to 2.05) | 11.84 (7.47 to 18.08) | -0.38% (-0.4 to -0.36) |
| Grenada | 277.69 (188.25 to 381.18) | 921.01 (624.36 to 1264.26) | 229.85 (152.98 to 313.83) | 989.15 (658.35 to 1350.54) | 0.23% (0.19 to 0.27) |
| Guam | 375.85 (275.36 to 497.53) | 989.82 (725.16 to 1310.26) | 379 (274.36 to 499.93) | 1061.45 (768.38 to 1400.13) | 0.26% (0.23 to 0.29) |
| Guatemala | 25875.92 (17237.11 to 36444.74) | 766.3 (510.47 to 1079.29) | 45384.18 (29857.34 to 63303.1) | 897.69 (590.57 to 1252.12) | 0.61% (0.55 to 0.67) |
| Guinea | 12720.66 (8445.25 to 17717.88) | 598.16 (397.12 to 833.14) | 37737.4 (25374.5 to 53008.11) | 723.77 (486.66 to 1016.65) | 0.41% (0.31 to 0.51) |
| Guinea-Bissau | 3273.38 (2185.48 to 4627.11) | 818.3 (546.34 to 1156.72) | 6876.32 (4548.18 to 9671.49) | 875 (578.75 to 1230.68) | 0.18% (0.14 to 0.22) |
| Guyana | 2492.68 (1652.27 to 3521.66) | 927.39 (614.72 to 1310.21) | 1919.76 (1270.29 to 2686.33) | 931.02 (616.05 to 1302.78) | 0.2% (0.09 to 0.31) |
| Haiti | 20016.01 (13060.79 to 28039.04) | 875.7 (571.41 to 1226.7) | 38447.37 (25155.62 to 53821.58) | 952.16 (622.99 to 1332.91) | 0.24% (0.18 to 0.31) |
| Honduras | 15735.19 (10611.31 to 22034.07) | 832.29 (561.27 to 1165.46) | 29392.1 (19603.96 to 40873.37) | 902.99 (602.28 to 1255.72) | 0.29% (0.27 to 0.3) |
| Hungary | 152.91 (96.1 to 229.23) | 6.7 (4.21 to 10.04) | 81.1 (48.91 to 121.29) | 5.71 (3.44 to 8.54) | -0.63% (-0.79 to -0.47) |
| Iceland | 2.99 (1.92 to 4.35) | 4.71 (3.03 to 6.86) | 3.38 (2.18 to 5.06) | 5.04 (3.24 to 7.54) | 0.14% (0.07 to 0.2) |
| India | 1240815.17 (816230.25 to 1719987.39) | 421.8 (277.47 to 584.69) | 1878504.72 (1212072.14 to 2626986.99) | 482.61 (311.4 to 674.91) | 1.78% (1.2 to 2.36) |
| Indonesia | 88800.41 (63283.88 to 119676.62) | 136.32 (97.15 to 183.71) | 104865.99 (72496.64 to 144288.64) | 153.51 (106.13 to 211.22) | 0.29% (0.24 to 0.34) |
| Iran (Islamic Republic of) | 110951.58 (75549.68 to 155269.19) | 487.97 (332.27 to 682.88) | 99644.61 (67412.45 to 138717.31) | 505.82 (342.2 to 704.17) | 0.1% (-0.07 to 0.28) |
| Iraq | 35507.07 (23531.67 to 49751.03) | 496.2 (328.85 to 695.26) | 69169.88 (47145.99 to 96104.96) | 518.38 (353.33 to 720.24) | 0.14% (0.13 to 0.15) |
| Ireland | 52.45 (32.44 to 76.54) | 5.04 (3.12 to 7.36) | 47.28 (28.09 to 70.04) | 4.62 (2.75 to 6.85) | -0.41% (-0.46 to -0.35) |
| Israel | 81.62 (52.08 to 119.67) | 5.48 (3.5 to 8.03) | 118.37 (73.18 to 171.97) | 4.82 (2.98 to 7) | -0.55% (-0.6 to -0.5) |
| Italy | 1384.75 (1056.24 to 1798.07) | 12.73 (9.71 to 16.54) | 747.04 (634.21 to 875.95) | 8.99 (7.63 to 10.54) | -1.44% (-1.89 to -0.98) |
| Jamaica | 7425.26 (5079.07 to 10301.92) | 912.75 (624.35 to 1266.37) | 6467.17 (4431.71 to 8755.12) | 1003.93 (687.96 to 1359.1) | 0.44% (0.39 to 0.49) |
| Japan | 2645.09 (2019.44 to 3423.62) | 9.9 (7.56 to 12.82) | 1283.87 (1056.4 to 1551.06) | 7.71 (6.35 to 9.32) | -1.24% (-1.51 to -0.98) |
| Jordan | 249.54 (173.21 to 351.2) | 16.68 (11.58 to 23.47) | 614.45 (423.19 to 894.01) | 16.05 (11.06 to 23.36) | -0.21% (-0.29 to -0.13) |
| Kazakhstan | 1420.94 (970.88 to 1983.68) | 29.85 (20.4 to 41.67) | 998.32 (627.26 to 1455.65) | 20.9 (13.13 to 30.48) | -1.47% (-1.59 to -1.34) |
| Kenya | 108441.05 (73582.11 to 150805.47) | 1141.62 (774.64 to 1587.62) | 245180.86 (165825.85 to 340126.32) | 1314.62 (889.13 to 1823.71) | 0.37% (0.29 to 0.46) |
| Kiribati | 237.54 (164.33 to 322.52) | 954.86 (660.56 to 1296.46) | 389.75 (267.79 to 535.58) | 995.46 (683.98 to 1367.92) | 0.12% (0.09 to 0.15) |
| Kuwait | 87.44 (60.19 to 123.76) | 18.16 (12.5 to 25.7) | 131.19 (89.68 to 186.16) | 15.8 (10.8 to 22.42) | -0.5% (-0.57 to -0.44) |
| Kyrgyzstan | 12206.96 (8369.22 to 16951) | 831.17 (569.86 to 1154.19) | 16187.57 (11032.53 to 22440.12) | 797.84 (543.76 to 1106.01) | -0.08% (-0.21 to 0.06) |
| Lao People's Democratic Republic | 10472.74 (6969.58 to 14541.97) | 672.4 (447.48 to 933.66) | 15010.48 (9943.41 to 21111.44) | 694.29 (459.92 to 976.48) | 0.32% (-0.11 to 0.75) |
| Latvia | 84.61 (53.74 to 125.2) | 15.39 (9.77 to 22.77) | 37.09 (24.08 to 56.17) | 12.64 (8.21 to 19.15) | -1.32% (-1.79 to -0.83) |
| Lebanon | 180.75 (126.16 to 250.33) | 19.04 (13.29 to 26.36) | 209.58 (140.58 to 304.26) | 16.63 (11.15 to 24.14) | -0.53% (-0.62 to -0.44) |
| Lesotho | 6723.32 (4466.63 to 9371.66) | 1137.58 (755.75 to 1585.68) | 7858.19 (5204.48 to 11124.52) | 1237.3 (819.46 to 1751.59) | 0.22% (0.19 to 0.25) |
| Liberia | 7354.76 (4896.3 to 10251.22) | 815.62 (542.98 to 1136.82) | 18476.84 (12081.47 to 25550.82) | 913.05 (597.01 to 1262.61) | 0.23% (0.17 to 0.3) |
| Libya | 7838.33 (5352.04 to 10737.4) | 468.98 (320.22 to 642.43) | 8908.18 (5943.63 to 12367.21) | 535.2 (357.09 to 743.02) | 0.4% (0.37 to 0.44) |
| Lithuania | 100.89 (67.04 to 151.2) | 12.33 (8.19 to 18.47) | 44.01 (28.18 to 65.6) | 10.89 (6.97 to 16.23) | -1.21% (-1.76 to -0.66) |
| Luxembourg | 4.83 (3.05 to 7.03) | 7.39 (4.67 to 10.77) | 7.14 (4.65 to 10.71) | 7.01 (4.57 to 10.52) | -0.35% (-0.61 to -0.1) |
| Madagascar | 54496.04 (35837.58 to 76439.74) | 1192.37 (784.13 to 1672.5) | 140715.19 (92795.52 to 196805.56) | 1296.23 (854.81 to 1812.92) | 0.24% (0.22 to 0.26) |
| Malawi | 46112.53 (30681.29 to 64146.23) | 1254.91 (834.96 to 1745.68) | 108138.25 (71104.1 to 149989.96) | 1374.62 (903.85 to 1906.62) | 0.21% (0.16 to 0.27) |
| Malaysia | 37353.96 (25677.61 to 50941.8) | 626.68 (430.79 to 854.64) | 51851.1 (35266.98 to 71240.39) | 665.03 (452.33 to 913.71) | 0.29% (0.25 to 0.33) |
| Maldives | 498.48 (341.68 to 692.37) | 575.86 (394.72 to 799.85) | 589.57 (399.46 to 806.47) | 598.26 (405.35 to 818.35) | 0.16% (0.03 to 0.28) |
| Mali | 28637.31 (18987.56 to 40625.32) | 890.27 (590.28 to 1262.95) | 90277.12 (59311.36 to 126287.5) | 935.25 (614.45 to 1308.31) | 0.13% (0.1 to 0.15) |
| Malta | 3.16 (1.85 to 4.75) | 3.63 (2.12 to 5.46) | 2.1 (1.26 to 3.2) | 3.4 (2.03 to 5.17) | -0.4% (-0.59 to -0.21) |
| Marshall Islands | 184.29 (129.72 to 249.06) | 952.64 (670.59 to 1287.46) | 179.69 (125.81 to 248.12) | 1032.76 (723.08 to 1426.07) | 0.17% (0.13 to 0.2) |
| Mauritania | 6530.34 (4420.52 to 9183.93) | 855.9 (579.38 to 1203.7) | 14784.51 (10108.79 to 20684.26) | 882.57 (603.45 to 1234.76) | 0.07% (0.05 to 0.09) |
| Mauritius | 2084.13 (1418.32 to 2818.22) | 640.59 (435.94 to 866.23) | 1576.99 (1079.25 to 2147.58) | 677.96 (463.98 to 923.26) | 0.06% (0.02 to 0.1) |
| Mexico | 129366.8 (88370.74 to 176831.52) | 409.79 (279.93 to 560.14) | 123096.9 (83867.47 to 169093.54) | 369.95 (252.05 to 508.19) | -0.39% (-0.41 to -0.37) |
| Micronesia (Federated States of) | 410.09 (282.21 to 561.02) | 980.18 (674.53 to 1340.91) | 335.54 (237.77 to 460.98) | 1049.4 (743.65 to 1441.74) | 0.17% (0.15 to 0.18) |
| Monaco | 0.18 (0.11 to 0.26) | 4.91 (3.07 to 7.08) | 0.24 (0.15 to 0.34) | 4.66 (2.92 to 6.7) | -0.11% (-0.15 to -0.06) |
| Mongolia | 6885.45 (4564.25 to 9739.39) | 865.45 (573.69 to 1224.16) | 7412.45 (5010.23 to 10277.3) | 803.95 (543.41 to 1114.67) | -0.14% (-0.3 to 0.03) |
| Montenegro | 9.86 (6.1 to 15.21) | 6.08 (3.76 to 9.39) | 6.93 (4.2 to 10.5) | 6.07 (3.68 to 9.19) | -0.04% (-0.08 to 0) |
| Morocco | 45614.48 (30480.3 to 63933.92) | 511.4 (341.73 to 716.79) | 51251.69 (33670.84 to 71200.67) | 530.29 (348.38 to 736.7) | 0.1% (0.05 to 0.14) |
| Mozambique | 64538 (43325.38 to 89539.87) | 1250.32 (839.36 to 1734.7) | 164202.74 (109354.62 to 230465.99) | 1305.79 (869.62 to 1832.74) | 0.05% (-0.05 to 0.15) |
| Myanmar | 93200.97 (62540.56 to 129413.74) | 662.45 (444.53 to 919.85) | 106215.39 (71462.02 to 150370.48) | 683.42 (459.81 to 967.53) | 0.07% (0.05 to 0.09) |
| Namibia | 6466.56 (4335.98 to 9048.13) | 1207.98 (809.98 to 1690.23) | 9709.75 (6546.82 to 13423.04) | 1224.54 (825.64 to 1692.83) | 0.04% (0 to 0.07) |
| Nauru | 3.07 (2.39 to 3.9) | 85.79 (66.93 to 109.08) | 3.19 (2.53 to 4.05) | 85.57 (67.87 to 108.51) | -0.09% (-0.22 to 0.04) |
| Nepal | 24286.66 (16893.36 to 33597.97) | 344.23 (239.44 to 476.2) | 40371.63 (26710.39 to 55668.66) | 431.87 (285.73 to 595.51) | 1% (0.86 to 1.13) |
| Netherlands | 136.2 (83.55 to 195.61) | 4.7 (2.88 to 6.75) | 131.67 (80.37 to 191.89) | 4.65 (2.84 to 6.78) | -0.1% (-0.15 to -0.05) |
| New Zealand | 170.01 (124.66 to 227.72) | 20.7 (15.18 to 27.72) | 207.18 (168.65 to 249.82) | 20.91 (17.02 to 25.21) | 0.33% (0.19 to 0.47) |
| Nicaragua | 13784.07 (9284.65 to 19100.02) | 868.69 (585.13 to 1203.71) | 18278.4 (12449.89 to 25359.73) | 937.97 (638.87 to 1301.35) | 0.19% (0.11 to 0.27) |
| Niger | 25088.62 (16718.2 to 34841.01) | 792.79 (528.29 to 1100.96) | 89034.36 (59208.27 to 125046.29) | 848.72 (564.4 to 1192) | 0.12% (0.08 to 0.15) |
| Nigeria | 300596.97 (203471.55 to 422031.22) | 921.4 (623.69 to 1293.63) | 915955.13 (621506.9 to 1282266.6) | 1010.38 (685.58 to 1414.46) | 0.25% (0.24 to 0.27) |
| Niue | 0.49 (0.39 to 0.62) | 64.48 (51.08 to 80.82) | 0.26 (0.2 to 0.32) | 62.66 (49.51 to 78.84) | -0.25% (-0.32 to -0.19) |
| North Macedonia | 40.95 (25.53 to 63.89) | 7.78 (4.85 to 12.13) | 21.28 (13.1 to 32.12) | 6.15 (3.79 to 9.28) | -1.12% (-1.24 to -1) |
| Northern Mariana Islands | 120.34 (87.66 to 159.11) | 1049.75 (764.61 to 1387.92) | 129.66 (94.22 to 171.49) | 1099.74 (799.2 to 1454.56) | 0.29% (0.22 to 0.36) |
| Norway | 26.87 (19.38 to 35.9) | 3.21 (2.32 to 4.29) | 30.72 (22.43 to 39.96) | 3.2 (2.34 to 4.16) | -0.17% (-0.36 to 0.02) |
| Oman | 105.38 (73.02 to 148.5) | 15.62 (10.82 to 22.01) | 173.57 (119.83 to 247.51) | 16.25 (11.22 to 23.18) | 0.23% (0.17 to 0.29) |
| Pakistan | 233946.71 (154502.99 to 328113.25) | 550.44 (363.52 to 772) | 493225.18 (324139.93 to 690892.2) | 620.43 (407.73 to 869.07) | 0.23% (-0.13 to 0.59) |
| Palau | 3.34 (2.65 to 4.18) | 71.32 (56.48 to 89.09) | 2.32 (1.82 to 2.93) | 66.73 (52.29 to 84.23) | -0.35% (-0.38 to -0.32) |
| Palestine | 3916.38 (2637.12 to 5520.61) | 487.36 (328.17 to 687) | 9014.44 (6095.24 to 12603.12) | 502.17 (339.55 to 702.09) | 0.17% (0.13 to 0.21) |
| Panama | 7035.32 (4738.37 to 9654.66) | 877.79 (591.21 to 1204.61) | 9988.08 (6745.59 to 13688.57) | 879.23 (593.8 to 1204.98) | 0.02% (-0.01 to 0.04) |
| Papua New Guinea | 13529.03 (9551 to 18468.46) | 913.54 (644.92 to 1247.07) | 32175.05 (22402.08 to 44050.39) | 952.56 (663.23 to 1304.14) | 0.15% (0.11 to 0.2) |
| Paraguay | 13135.72 (8943.21 to 18168.67) | 904.61 (615.89 to 1251.21) | 19629.44 (13116.44 to 27021.98) | 971.47 (649.14 to 1337.33) | 0.24% (0.2 to 0.28) |
| Peru | 66112.92 (44464.66 to 91859.06) | 855.97 (575.69 to 1189.31) | 82677.87 (55417.27 to 115721.68) | 897.7 (601.71 to 1256.48) | 0.2% (0.18 to 0.23) |
| Philippines | 97231.69 (65218.04 to 135180.32) | 426.43 (286.03 to 592.86) | 179392.05 (121569.32 to 251883.91) | 534.47 (362.2 to 750.45) | 1.27% (1.02 to 1.53) |
| Poland | 911.9 (689.47 to 1230.97) | 9.57 (7.23 to 12.92) | 473.74 (391.06 to 572.12) | 8.14 (6.72 to 9.84) | -0.75% (-1.08 to -0.41) |
| Portugal | 178.44 (115.3 to 266.76) | 7.43 (4.8 to 11.1) | 81.83 (50.6 to 122.72) | 5.49 (3.39 to 8.23) | -1.23% (-1.39 to -1.06) |
| Puerto Rico | 136.6 (88.23 to 200.97) | 13.49 (8.71 to 19.84) | 72.15 (47.47 to 108.77) | 13.16 (8.66 to 19.84) | -0.16% (-0.25 to -0.07) |
| Qatar | 24.9 (17.85 to 34.62) | 24.35 (17.46 to 33.85) | 69.06 (46.86 to 98.51) | 16.73 (11.35 to 23.86) | -1.6% (-1.72 to -1.49) |
| Republic of Korea | 1060.11 (707.99 to 1500.89) | 8.41 (5.62 to 11.9) | 497.99 (314.62 to 722.35) | 7.29 (4.6 to 10.57) | -0.64% (-0.7 to -0.58) |
| Republic of Moldova | 234.54 (149.44 to 341.67) | 20.39 (12.99 to 29.7) | 65.81 (43.14 to 100.76) | 12.14 (7.96 to 18.59) | -2.05% (-2.16 to -1.94) |
| Romania | 627.37 (398.66 to 941.53) | 10.94 (6.95 to 16.42) | 273.88 (175.57 to 419.38) | 8.83 (5.66 to 13.52) | -1.87% (-2.7 to -1.03) |
| Russian Federation | 8936.21 (7012.16 to 10923.7) | 26.63 (20.89 to 32.55) | 6585.46 (5253.78 to 8020.15) | 25.16 (20.07 to 30.64) | -0.51% (-0.65 to -0.36) |
| Rwanda | 33548.24 (22180.69 to 46912.17) | 1202.1 (794.78 to 1680.95) | 60768.13 (40555.84 to 86165.68) | 1295.8 (864.8 to 1837.37) | 0.2% (0.14 to 0.27) |
| Saint Kitts and Nevis | 3.12 (2.09 to 4.42) | 22.79 (15.29 to 32.28) | 1.63 (1.11 to 2.4) | 14.78 (10.1 to 21.79) | -1.78% (-1.93 to -1.62) |
| Saint Lucia | 473.35 (318.65 to 663.9) | 963.7 (648.74 to 1351.63) | 340.35 (229.63 to 471.76) | 1027.47 (693.22 to 1424.2) | 0.29% (0.26 to 0.32) |
| Saint Vincent and the Grenadines | 383.9 (254.6 to 540.05) | 936.92 (621.36 to 1317.99) | 255.08 (170.85 to 350.1) | 962.28 (644.52 to 1320.75) | 0.09% (0.06 to 0.12) |
| Samoa | 680.99 (488.41 to 921.99) | 1030.17 (738.86 to 1394.76) | 757.24 (536.43 to 1021.57) | 1056.15 (748.17 to 1424.82) | 0.07% (0.06 to 0.09) |
| San Marino | 0.24 (0.15 to 0.35) | 5.08 (3.11 to 7.39) | 0.24 (0.15 to 0.35) | 4.83 (3.02 to 6.96) | -0.24% (-0.3 to -0.18) |
| Sao Tome and Principe | 438.67 (295.9 to 612.38) | 887.93 (598.95 to 1239.55) | 748.6 (502.45 to 1061.81) | 973.99 (653.72 to 1381.5) | 0.19% (0.14 to 0.24) |
| Saudi Arabia | 1258.73 (896.57 to 1724.91) | 21.73 (15.47 to 29.77) | 1288.78 (864.31 to 1812.51) | 16.74 (11.22 to 23.54) | -1.04% (-1.1 to -0.98) |
| Senegal | 26184.26 (17679.16 to 36717.85) | 878.11 (592.88 to 1231.36) | 54248.09 (36480.36 to 76093.47) | 928.29 (624.25 to 1302.11) | -0.03% (-0.13 to 0.06) |
| Serbia | 116.72 (71.3 to 173.74) | 5.26 (3.21 to 7.82) | 90.65 (54.7 to 140.28) | 6.02 (3.63 to 9.32) | -0.7% (-1.62 to 0.24) |
| Seychelles | 153.3 (105.66 to 206.32) | 661.24 (455.73 to 889.95) | 144.14 (99.4 to 194.16) | 636.27 (438.78 to 857.09) | -0.13% (-0.15 to -0.11) |
| Sierra Leone | 12120.24 (8142.23 to 16940.39) | 846.44 (568.63 to 1183.06) | 29458.31 (19532.63 to 41327.47) | 918.36 (608.93 to 1288.38) | 0.31% (0.28 to 0.33) |
| Singapore | 80.16 (52.96 to 117.36) | 11.18 (7.39 to 16.37) | 55.12 (36.05 to 80.29) | 7.28 (4.76 to 10.6) | -1.78% (-1.95 to -1.6) |
| Slovakia | 114.02 (74.65 to 170.84) | 8.47 (5.55 to 12.69) | 72.02 (45.45 to 110.32) | 8.63 (5.44 to 13.21) | 0% (-1.35 to 1.38) |
| Slovenia | 18.71 (11.02 to 27.78) | 4.31 (2.54 to 6.4) | 13.23 (7.96 to 19.62) | 4.31 (2.59 to 6.39) | -0.13% (-0.53 to 0.27) |
| Solomon Islands | 1283.6 (888.62 to 1736.06) | 951.08 (658.42 to 1286.33) | 2310 (1620.83 to 3174.18) | 981.29 (688.53 to 1348.39) | 0.04% (0.02 to 0.06) |
| Somalia | 37653.19 (24783.69 to 51956.9) | 1181.35 (777.58 to 1630.12) | 108957.77 (71038.14 to 153696.29) | 1258.85 (820.74 to 1775.74) | 0.09% (0.03 to 0.15) |
| South Africa | 173935.12 (117214.52 to 242073.67) | 1364.9 (919.8 to 1899.59) | 207630.94 (141182.12 to 288093.05) | 1388.21 (943.94 to 1926.18) | 0.01% (-0.06 to 0.08) |
| South Sudan | 27265.63 (18166.9 to 38264.47) | 1202.05 (800.92 to 1686.95) | 51140.49 (33575.4 to 71203.18) | 1302.94 (855.42 to 1814.09) | 0.19% (0.14 to 0.24) |
| Spain | 646.21 (418.44 to 937.69) | 7.13 (4.62 to 10.34) | 345.79 (226.66 to 482.71) | 4.96 (3.25 to 6.93) | -1.76% (-2.01 to -1.51) |
| Sri Lanka | 1013.11 (703.38 to 1415.52) | 18.51 (12.85 to 25.87) | 1003.14 (659.97 to 1468.38) | 18.81 (12.37 to 27.53) | 0.01% (-0.11 to 0.12) |
| Sudan | 31085.98 (21072.69 to 42879.82) | 410.91 (278.55 to 566.8) | 69118.37 (46449.49 to 95744.97) | 439.24 (295.18 to 608.45) | 0.22% (0.21 to 0.24) |
| Suriname | 1137.94 (768.34 to 1584.96) | 894.7 (604.1 to 1246.17) | 1310.19 (873.81 to 1824.18) | 901.62 (601.32 to 1255.32) | 0.02% (0 to 0.05) |
| Sweden | 54.58 (40.09 to 73.13) | 3.54 (2.6 to 4.74) | 51.16 (36.85 to 66.98) | 2.79 (2.01 to 3.66) | -0.98% (-1.65 to -0.3) |
| Switzerland | 79.77 (50.88 to 120.76) | 6.77 (4.32 to 10.24) | 82.61 (50.42 to 123.94) | 6.26 (3.82 to 9.39) | -0.86% (-1.35 to -0.37) |
| Syrian Arab Republic | 24514 (16537.62 to 34033.25) | 471.88 (318.34 to 655.12) | 24149.63 (15810.88 to 33524.25) | 544.52 (356.5 to 755.9) | 0.29% (0.21 to 0.38) |
| Taiwan (Province of China) | 633.37 (422.71 to 949.22) | 11.11 (7.41 to 16.65) | 504.23 (368.78 to 664.26) | 15.99 (11.7 to 21.07) | 1.75% (1.35 to 2.15) |
| Tajikistan | 16124.76 (10891.73 to 22646.48) | 835.94 (564.65 to 1174.04) | 25808.81 (17619.56 to 35888.13) | 828.09 (565.34 to 1151.5) | 0.13% (0.02 to 0.24) |
| Thailand | 107227.01 (72669.4 to 149615.97) | 611.1 (414.15 to 852.68) | 65997.73 (44746.29 to 90786.7) | 609.9 (413.51 to 838.97) | -0.01% (-0.03 to 0.02) |
| Timor-Leste | 1552.42 (1050.28 to 2172.24) | 582.27 (393.93 to 814.75) | 3114.67 (2079.51 to 4295.14) | 622.21 (415.42 to 858.03) | 0.22% (0.19 to 0.24) |
| Togo | 12323.08 (8356.66 to 17089.35) | 840.35 (569.87 to 1165.37) | 26585.27 (17832.71 to 37355.14) | 885.32 (593.85 to 1243.97) | 0.1% (0.07 to 0.12) |
| Tokelau | 0.36 (0.29 to 0.46) | 64.33 (51.2 to 81.82) | 0.23 (0.18 to 0.29) | 55.31 (42.64 to 70.85) | -0.7% (-0.77 to -0.62) |
| Tonga | 517.88 (361.38 to 703.14) | 1359.71 (948.82 to 1846.11) | 491.98 (352.78 to 664.18) | 1398.7 (1002.95 to 1888.24) | -0.19% (-0.61 to 0.23) |
| Trinidad and Tobago | 3439.03 (2331.26 to 4690.81) | 890.58 (603.71 to 1214.74) | 2711.54 (1813.53 to 3720.66) | 956.55 (639.76 to 1312.54) | 0.12% (-0.01 to 0.26) |
| Tunisia | 651.57 (474.05 to 891.04) | 22.29 (16.22 to 30.48) | 442.85 (299.66 to 636.61) | 16.44 (11.13 to 23.63) | -1.26% (-1.37 to -1.14) |
| Türkiye | 3814.53 (2731.2 to 5326.73) | 19.34 (13.85 to 27) | 3100.21 (2097.78 to 4416.32) | 16.18 (10.95 to 23.05) | -0.77% (-0.9 to -0.65) |
| Turkmenistan | 10818.97 (7327.81 to 15195) | 832.46 (563.83 to 1169.17) | 11959.06 (8020.51 to 16870.82) | 841.69 (564.49 to 1187.38) | 0.1% (0.05 to 0.15) |
| Tuvalu | 2.01 (1.56 to 2.61) | 72.79 (56.61 to 94.34) | 2.13 (1.66 to 2.82) | 58.81 (45.74 to 77.67) | -0.96% (-1.03 to -0.88) |
| Uganda | 83080.05 (56080.66 to 114732.27) | 1238.83 (836.24 to 1710.81) | 220479.81 (146324.12 to 306577.85) | 1250.89 (830.17 to 1739.37) | 0.24% (-0.04 to 0.52) |
| Ukraine | 1328.24 (972.02 to 1789.98) | 11.77 (8.62 to 15.86) | 828.16 (624.48 to 1085.92) | 12.08 (9.11 to 15.85) | 0.17% (0.1 to 0.23) |
| United Arab Emirates | 2261.25 (1538.99 to 3050.97) | 472.01 (321.25 to 636.85) | 6136.28 (4140.38 to 8388.08) | 486.08 (327.98 to 664.45) | 0.07% (-0.01 to 0.14) |
| United Kingdom | 618.01 (469.23 to 803.41) | 5.62 (4.27 to 7.31) | 592.76 (450.91 to 761.97) | 4.94 (3.76 to 6.35) | -0.45% (-0.49 to -0.42) |
| United Republic of Tanzania | 124339.07 (82074.14 to 172527.58) | 1229.23 (811.39 to 1705.63) | 296064.16 (197750.04 to 414064.37) | 1345.01 (898.37 to 1881.08) | 0.27% (0.24 to 0.3) |
| United States of America | 4837.41 (3721.36 to 6307.99) | 8.91 (6.85 to 11.62) | 5722.78 (4713.4 to 6962.24) | 9.12 (7.52 to 11.1) | 0.8% (0.42 to 1.19) |
| United States Virgin Islands | 5.31 (3.58 to 7.52) | 17.29 (11.64 to 24.48) | 1.92 (1.26 to 2.86) | 13.45 (8.8 to 19.97) | -1.3% (-1.45 to -1.15) |
| Uruguay | 95.39 (60.3 to 142.59) | 11.86 (7.5 to 17.73) | 84.52 (54.52 to 130.51) | 11.92 (7.69 to 18.4) | -0.01% (-0.08 to 0.06) |
| Uzbekistan | 58296.09 (39601.44 to 82091.16) | 798.27 (542.28 to 1124.1) | 73472.79 (49242.17 to 101863.23) | 837.31 (561.17 to 1160.85) | 0.36% (0.24 to 0.49) |
| Vanuatu | 592.27 (416.94 to 797.23) | 1049.98 (739.14 to 1413.32) | 1146.84 (809.15 to 1558.48) | 1089.25 (768.52 to 1480.22) | 0.08% (0.05 to 0.1) |
| Venezuela (Bolivarian Republic of) | 1204.97 (788.26 to 1707.02) | 18.41 (12.04 to 26.08) | 993.88 (652.41 to 1450.77) | 15.13 (9.93 to 22.09) | -0.85% (-1.02 to -0.69) |
| Viet Nam | 6069.66 (4294.03 to 8300.52) | 25.02 (17.7 to 34.22) | 5205.3 (3825.73 to 6926.05) | 22.17 (16.3 to 29.5) | -0.58% (-0.67 to -0.49) |
| Yemen | 26828.72 (18913.28 to 36863.08) | 476.4 (335.85 to 654.59) | 71441.23 (47470.76 to 99727.6) | 559.24 (371.6 to 780.66) | 0.33% (-0.38 to 1.04) |
| Zambia | 43999.61 (30687.86 to 59012.78) | 1378.73 (961.6 to 1849.16) | 100273.88 (66454.85 to 139538.71) | 1331.8 (882.63 to 1853.3) | -0.1% (-0.19 to -0.02) |
| Zimbabwe | 48563.34 (32713.66 to 67879.24) | 1140.3 (768.14 to 1593.85) | 68817.92 (46844.16 to 95728.52) | 1191.75 (811.22 to 1657.77) | 0.08% (0.01 to 0.14) |

Table S16. Deaths of Rheumatic heart disease in 1990 and 2021 for both sexes in 204 countries, with EAPC from 1990 and 2021.

| location | Num_1990 | ASR_1990 | Num_2021 | ASR_2021 | EAPC_CI |
| --- | --- | --- | --- | --- | --- |
| Afghanistan | 77.45 (38.48 to 122.78) | 2.01 (1 to 3.18) | 94.46 (49.84 to 155.94) | 0.77 (0.41 to 1.27) | -3.07% (-3.28 to -2.87) |
| Albania | 2.63 (2.02 to 3.5) | 0.25 (0.19 to 0.34) | 0.19 (0.12 to 0.28) | 0.04 (0.03 to 0.06) | -4.63% (-5.15 to -4.11) |
| Algeria | 99.11 (60.46 to 146.71) | 1.01 (0.62 to 1.5) | 22.38 (15.13 to 33.62) | 0.19 (0.13 to 0.29) | -5.07% (-5.19 to -4.94) |
| American Samoa | 0.25 (0.19 to 0.31) | 1.53 (1.18 to 1.91) | 0.18 (0.13 to 0.23) | 1.14 (0.82 to 1.5) | -1.09% (-1.16 to -1.02) |
| Andorra | 0 (0 to 0) | 0.03 (0.02 to 0.04) | 0 (0 to 0) | 0.01 (0.01 to 0.01) | -4% (-4.17 to -3.83) |
| Angola | 34.12 (21.13 to 51) | 0.89 (0.55 to 1.34) | 36.24 (22.9 to 57.43) | 0.28 (0.18 to 0.44) | -3.67% (-3.93 to -3.41) |
| Antigua and Barbuda | 0.06 (0.06 to 0.07) | 0.36 (0.31 to 0.41) | 0.03 (0.02 to 0.03) | 0.14 (0.12 to 0.17) | -3.69% (-4.17 to -3.2) |
| Argentina | 18.08 (15.98 to 20.52) | 0.19 (0.17 to 0.21) | 3.53 (3.02 to 4.1) | 0.03 (0.03 to 0.04) | -5.36% (-5.59 to -5.13) |
| Armenia | 4.54 (3.78 to 5.18) | 0.48 (0.4 to 0.55) | 0.55 (0.42 to 0.63) | 0.1 (0.07 to 0.11) | -4.19% (-4.99 to -3.38) |
| Australia | 4.13 (3.6 to 4.71) | 0.11 (0.09 to 0.12) | 1.54 (1.31 to 1.76) | 0.03 (0.03 to 0.04) | -3.79% (-4.04 to -3.55) |
| Austria | 0.58 (0.51 to 0.67) | 0.04 (0.04 to 0.05) | 0.23 (0.2 to 0.27) | 0.02 (0.02 to 0.02) | -2.86% (-3.07 to -2.65) |
| Azerbaijan | 9.72 (7.74 to 11.44) | 0.44 (0.35 to 0.51) | 4.83 (3.03 to 6.39) | 0.21 (0.13 to 0.27) | -2.87% (-3.45 to -2.29) |
| Bahamas | 0.25 (0.21 to 0.29) | 0.3 (0.26 to 0.36) | 0.1 (0.07 to 0.13) | 0.11 (0.08 to 0.14) | -3.79% (-4.25 to -3.32) |
| Bahrain | 0.96 (0.74 to 1.23) | 0.69 (0.53 to 0.88) | 0.32 (0.22 to 0.43) | 0.1 (0.07 to 0.14) | -6.49% (-7.13 to -5.84) |
| Bangladesh | 1184.1 (814.43 to 1691.21) | 2.86 (1.97 to 4.08) | 658.81 (476.51 to 876.52) | 1.41 (1.02 to 1.88) | -1.83% (-2.04 to -1.63) |
| Barbados | 0.28 (0.24 to 0.33) | 0.43 (0.37 to 0.51) | 0.06 (0.04 to 0.08) | 0.11 (0.08 to 0.15) | -3.95% (-4.29 to -3.59) |
| Belarus | 2.62 (2.07 to 3.24) | 0.11 (0.09 to 0.14) | 0.28 (0.22 to 0.34) | 0.02 (0.01 to 0.02) | -6.11% (-7.01 to -5.21) |
| Belgium | 0.72 (0.61 to 0.89) | 0.04 (0.03 to 0.05) | 0.28 (0.24 to 0.33) | 0.01 (0.01 to 0.02) | -3.59% (-3.83 to -3.34) |
| Belize | 0.34 (0.3 to 0.39) | 0.47 (0.41 to 0.53) | 0.18 (0.15 to 0.21) | 0.14 (0.12 to 0.16) | -3.96% (-4.34 to -3.58) |
| Benin | 10.92 (7.34 to 15.16) | 0.58 (0.39 to 0.81) | 10.38 (6.99 to 14.77) | 0.2 (0.14 to 0.29) | -3.12% (-3.42 to -2.83) |
| Bermuda | 0.02 (0.02 to 0.03) | 0.19 (0.16 to 0.22) | 0.01 (0 to 0.01) | 0.06 (0.05 to 0.08) | -4.53% (-4.85 to -4.21) |
| Bhutan | 6.16 (3.01 to 9.97) | 2.51 (1.23 to 4.07) | 2.08 (1.37 to 3.05) | 1.07 (0.71 to 1.57) | -2.91% (-3.11 to -2.72) |
| Bolivia (Plurinational State of) | 14.95 (9.4 to 25.03) | 0.64 (0.4 to 1.07) | 4.96 (3.24 to 7.69) | 0.15 (0.1 to 0.23) | -4.82% (-4.91 to -4.73) |
| Bosnia and Herzegovina | 0.72 (0.51 to 0.99) | 0.06 (0.04 to 0.09) | 0.06 (0.04 to 0.09) | 0.01 (0.01 to 0.02) | -5.27% (-5.84 to -4.69) |
| Botswana | 5.41 (3.68 to 7.66) | 1.02 (0.7 to 1.45) | 2.57 (1.85 to 3.57) | 0.38 (0.27 to 0.52) | -2.96% (-3.21 to -2.7) |
| Brazil | 321.27 (298.54 to 344.95) | 0.63 (0.59 to 0.68) | 64.04 (56.27 to 70.71) | 0.14 (0.12 to 0.15) | -4.71% (-4.91 to -4.51) |
| Brunei Darussalam | 0.1 (0.07 to 0.13) | 0.12 (0.09 to 0.17) | 0.03 (0.02 to 0.03) | 0.03 (0.02 to 0.03) | -4.88% (-5.24 to -4.52) |
| Bulgaria | 5.46 (4.71 to 6.24) | 0.3 (0.26 to 0.34) | 0.61 (0.47 to 0.75) | 0.06 (0.05 to 0.08) | -5.07% (-5.41 to -4.74) |
| Burkina Faso | 25.08 (17.12 to 34.95) | 0.66 (0.45 to 0.92) | 27.9 (17.44 to 40.63) | 0.32 (0.2 to 0.47) | -1.96% (-2.27 to -1.64) |
| Burundi | 20.15 (13.9 to 27.1) | 0.97 (0.67 to 1.3) | 13.56 (8.62 to 20.93) | 0.27 (0.17 to 0.41) | -4.43% (-4.57 to -4.29) |
| Cabo Verde | 0.84 (0.6 to 1.13) | 0.62 (0.45 to 0.83) | 0.2 (0.13 to 0.29) | 0.14 (0.09 to 0.2) | -4.98% (-5.63 to -4.33) |
| Cambodia | 73.86 (48.56 to 104.89) | 1.91 (1.26 to 2.71) | 28.34 (19.31 to 39.37) | 0.58 (0.4 to 0.81) | -4.22% (-4.43 to -4) |
| Cameroon | 21.87 (14.89 to 31.96) | 0.56 (0.38 to 0.81) | 26.48 (16.96 to 41.53) | 0.22 (0.14 to 0.34) | -2.64% (-2.82 to -2.45) |
| Canada | 2.98 (2.64 to 3.37) | 0.05 (0.05 to 0.06) | 0.83 (0.72 to 0.96) | 0.01 (0.01 to 0.02) | -4.54% (-5.06 to -4.01) |
| Central African Republic | 10.33 (6.53 to 14.45) | 1.04 (0.66 to 1.45) | 11.37 (6.26 to 19.62) | 0.55 (0.3 to 0.95) | -2.05% (-2.14 to -1.97) |
| Chad | 18.32 (11.88 to 26.38) | 0.8 (0.52 to 1.16) | 34.4 (22.91 to 49.68) | 0.47 (0.31 to 0.68) | -1.4% (-1.51 to -1.28) |
| Chile | 5.27 (4.58 to 6) | 0.14 (0.12 to 0.16) | 1.01 (0.84 to 1.19) | 0.03 (0.02 to 0.03) | -4.45% (-4.72 to -4.18) |
| China | 2187.19 (1824.88 to 2586.19) | 0.66 (0.55 to 0.78) | 169.27 (136.75 to 203.82) | 0.07 (0.05 to 0.08) | -7.3% (-7.46 to -7.13) |
| Colombia | 27.32 (24.14 to 30.85) | 0.25 (0.22 to 0.28) | 2.96 (2.36 to 3.59) | 0.03 (0.02 to 0.03) | -7.79% (-8.2 to -7.38) |
| Comoros | 0.96 (0.31 to 1.53) | 0.53 (0.17 to 0.84) | 0.41 (0.23 to 0.66) | 0.18 (0.1 to 0.28) | -4.17% (-4.88 to -3.46) |
| Congo | 5.51 (3.45 to 7.82) | 0.59 (0.37 to 0.84) | 4.26 (2.68 to 6.56) | 0.23 (0.14 to 0.35) | -3.24% (-3.57 to -2.91) |
| Cook Islands | 0.08 (0.06 to 0.11) | 1.27 (0.92 to 1.66) | 0.02 (0.01 to 0.02) | 0.45 (0.33 to 0.59) | -3.74% (-3.96 to -3.52) |
| Costa Rica | 1.9 (1.67 to 2.09) | 0.19 (0.16 to 0.21) | 0.34 (0.28 to 0.39) | 0.03 (0.03 to 0.04) | -5.71% (-6.02 to -5.4) |
| Côte d'Ivoire | 28.8 (18.87 to 42.96) | 0.62 (0.41 to 0.93) | 26.43 (17.79 to 38.79) | 0.26 (0.18 to 0.39) | -2.3% (-2.47 to -2.13) |
| Croatia | 1.25 (1.03 to 1.52) | 0.12 (0.1 to 0.15) | 0.09 (0.07 to 0.11) | 0.01 (0.01 to 0.02) | -7.22% (-7.75 to -6.7) |
| Cuba | 20.98 (18.97 to 23.36) | 0.76 (0.69 to 0.85) | 3.4 (2.94 to 3.95) | 0.18 (0.16 to 0.21) | -4.41% (-4.68 to -4.14) |
| Cyprus | 0.12 (0.08 to 0.2) | 0.06 (0.04 to 0.1) | 0.03 (0.02 to 0.04) | 0.01 (0.01 to 0.02) | -3.86% (-4.66 to -3.06) |
| Czechia | 3.41 (3.06 to 3.87) | 0.14 (0.13 to 0.16) | 0.26 (0.22 to 0.3) | 0.02 (0.01 to 0.02) | -7.3% (-7.65 to -6.95) |
| Democratic People's Republic of Korea | 17.94 (11.55 to 27.57) | 0.33 (0.21 to 0.51) | 6.72 (3.87 to 11.86) | 0.13 (0.08 to 0.23) | -2.75% (-2.93 to -2.56) |
| Democratic Republic of the Congo | 91.93 (56.52 to 128.75) | 0.64 (0.39 to 0.9) | 84.38 (43.75 to 160.55) | 0.25 (0.13 to 0.47) | -2.82% (-2.97 to -2.68) |
| Denmark | 0.43 (0.36 to 0.49) | 0.05 (0.04 to 0.05) | 0.19 (0.16 to 0.21) | 0.02 (0.02 to 0.02) | -3.51% (-4.02 to -3) |
| Djibouti | 0.71 (0.38 to 1.09) | 0.44 (0.24 to 0.68) | 0.56 (0.31 to 0.95) | 0.15 (0.08 to 0.25) | -3.7% (-4.01 to -3.4) |
| Dominica | 0.14 (0.1 to 0.17) | 0.56 (0.43 to 0.72) | 0.06 (0.04 to 0.08) | 0.36 (0.26 to 0.47) | -1.37% (-1.66 to -1.08) |
| Dominican Republic | 25.31 (20.68 to 31.15) | 1.01 (0.83 to 1.24) | 12.85 (9.06 to 18.17) | 0.45 (0.32 to 0.64) | -2.28% (-2.55 to -2.02) |
| Ecuador | 14.51 (12.88 to 16.44) | 0.4 (0.36 to 0.46) | 3.36 (2.57 to 4.09) | 0.07 (0.05 to 0.08) | -4.9% (-5.41 to -4.38) |
| Egypt | 1235.09 (763.79 to 1761.45) | 6.37 (3.94 to 9.08) | 306.83 (223.18 to 426.22) | 0.92 (0.67 to 1.27) | -6.03% (-6.14 to -5.91) |
| El Salvador | 3.36 (2.65 to 4.19) | 0.17 (0.13 to 0.21) | 0.57 (0.43 to 0.78) | 0.03 (0.02 to 0.04) | -5.09% (-5.61 to -4.57) |
| Equatorial Guinea | 1.41 (0.85 to 2.08) | 0.89 (0.54 to 1.32) | 0.85 (0.43 to 1.63) | 0.15 (0.07 to 0.28) | -7.03% (-7.53 to -6.53) |
| Eritrea | 10.11 (7 to 13.99) | 0.75 (0.52 to 1.04) | 5.85 (3.61 to 8.93) | 0.25 (0.16 to 0.39) | -3.38% (-3.5 to -3.27) |
| Estonia | 0.71 (0.6 to 0.84) | 0.21 (0.18 to 0.25) | 0.02 (0.02 to 0.03) | 0.01 (0.01 to 0.01) | -10.38% (-10.85 to -9.91) |
| Eswatini | 4.03 (2.9 to 5.46) | 1.22 (0.88 to 1.65) | 2.79 (1.81 to 3.97) | 0.71 (0.46 to 1.01) | -1.31% (-1.67 to -0.95) |
| Ethiopia | 140.22 (98.85 to 193.96) | 0.71 (0.5 to 0.98) | 78.65 (52.13 to 125.97) | 0.19 (0.13 to 0.31) | -4.78% (-4.98 to -4.58) |
| Fiji | 12.15 (9.95 to 14.82) | 4.59 (3.76 to 5.6) | 7.89 (5.98 to 10.45) | 3.04 (2.3 to 4.03) | -1.67% (-1.95 to -1.39) |
| Finland | 0.45 (0.39 to 0.51) | 0.05 (0.04 to 0.05) | 0.22 (0.18 to 0.25) | 0.02 (0.02 to 0.03) | -2.35% (-2.56 to -2.14) |
| France | 4.86 (4.31 to 5.52) | 0.04 (0.04 to 0.05) | 1.62 (1.39 to 1.85) | 0.01 (0.01 to 0.02) | -3.71% (-4 to -3.43) |
| Gabon | 1.42 (0.86 to 2.04) | 0.4 (0.24 to 0.58) | 0.89 (0.55 to 1.33) | 0.15 (0.09 to 0.22) | -3.26% (-3.45 to -3.08) |
| Gambia | 2.37 (1.58 to 3.43) | 0.63 (0.42 to 0.91) | 2.5 (1.61 to 3.77) | 0.28 (0.18 to 0.41) | -2.66% (-2.99 to -2.34) |
| Georgia | 14.08 (10.69 to 15.74) | 1.06 (0.81 to 1.19) | 1.31 (1.13 to 1.5) | 0.19 (0.16 to 0.22) | -6.21% (-7.67 to -4.72) |
| Germany | 8.36 (7.49 to 9.43) | 0.07 (0.06 to 0.07) | 3.38 (2.98 to 3.83) | 0.03 (0.03 to 0.03) | -2.64% (-2.95 to -2.32) |
| Ghana | 22.48 (15.82 to 30.79) | 0.4 (0.28 to 0.55) | 11.56 (7.73 to 16.62) | 0.1 (0.07 to 0.14) | -4.71% (-5.03 to -4.4) |
| Greece | 0.36 (0.32 to 0.41) | 0.02 (0.01 to 0.02) | 0.14 (0.12 to 0.17) | 0.01 (0.01 to 0.01) | -1.91% (-2.37 to -1.44) |
| Greenland | 0.01 (0 to 0.01) | 0.06 (0.04 to 0.08) | 0 (0 to 0) | 0.01 (0.01 to 0.01) | -6.53% (-6.95 to -6.12) |
| Grenada | 0.29 (0.25 to 0.34) | 0.97 (0.84 to 1.11) | 0.08 (0.07 to 0.1) | 0.36 (0.31 to 0.42) | -3.01% (-3.42 to -2.6) |
| Guam | 0.46 (0.37 to 0.56) | 1.2 (0.97 to 1.47) | 0.2 (0.13 to 0.26) | 0.55 (0.37 to 0.74) | -1% (-1.91 to -0.09) |
| Guatemala | 5.05 (4.5 to 5.69) | 0.15 (0.13 to 0.17) | 0.99 (0.79 to 1.21) | 0.02 (0.02 to 0.02) | -6.39% (-6.63 to -6.15) |
| Guinea | 19.93 (12.69 to 29.5) | 0.94 (0.6 to 1.39) | 18.72 (11.62 to 28.46) | 0.36 (0.22 to 0.55) | -2.67% (-2.91 to -2.43) |
| Guinea-Bissau | 5.44 (3.68 to 7.85) | 1.36 (0.92 to 1.96) | 3.49 (2.42 to 5.42) | 0.44 (0.31 to 0.69) | -3.31% (-3.45 to -3.17) |
| Guyana | 2.78 (2.15 to 3.31) | 1.03 (0.8 to 1.23) | 0.6 (0.47 to 0.79) | 0.29 (0.23 to 0.38) | -2.68% (-3.15 to -2.2) |
| Haiti | 86.27 (51.45 to 118) | 3.77 (2.25 to 5.16) | 70.3 (40.29 to 99.13) | 1.74 (1 to 2.46) | -2.13% (-2.36 to -1.9) |
| Honduras | 1.44 (1.07 to 1.92) | 0.08 (0.06 to 0.1) | 0.61 (0.35 to 0.95) | 0.02 (0.01 to 0.03) | -4.56% (-4.65 to -4.47) |
| Hungary | 3.26 (2.83 to 3.7) | 0.14 (0.12 to 0.16) | 0.23 (0.18 to 0.27) | 0.02 (0.01 to 0.02) | -6.8% (-7.3 to -6.3) |
| Iceland | 0.02 (0.02 to 0.02) | 0.03 (0.03 to 0.04) | 0.01 (0.01 to 0.01) | 0.01 (0.01 to 0.02) | -3.39% (-3.74 to -3.04) |
| India | 8098.21 (6558.47 to 10291.19) | 2.75 (2.23 to 3.5) | 3891.05 (3312.08 to 4470.38) | 1 (0.85 to 1.15) | -3.42% (-3.81 to -3.03) |
| Indonesia | 1034.63 (794.09 to 1264.51) | 1.59 (1.22 to 1.94) | 376.52 (263.88 to 502.46) | 0.55 (0.39 to 0.74) | -3.4% (-3.52 to -3.29) |
| Iran (Islamic Republic of) | 185.31 (133.8 to 249.27) | 0.82 (0.59 to 1.1) | 40.2 (30.8 to 52.45) | 0.2 (0.16 to 0.27) | -4.21% (-4.56 to -3.86) |
| Iraq | 80.39 (55.79 to 109.53) | 1.12 (0.78 to 1.53) | 33.77 (24.61 to 43.48) | 0.25 (0.18 to 0.33) | -4.47% (-4.82 to -4.11) |
| Ireland | 0.34 (0.29 to 0.39) | 0.03 (0.03 to 0.04) | 0.09 (0.07 to 0.11) | 0.01 (0.01 to 0.01) | -4.38% (-4.96 to -3.8) |
| Israel | 0.9 (0.79 to 1.03) | 0.06 (0.05 to 0.07) | 0.47 (0.41 to 0.55) | 0.02 (0.02 to 0.02) | -3.44% (-3.72 to -3.16) |
| Italy | 10.72 (10.19 to 11.27) | 0.1 (0.09 to 0.1) | 3.28 (3.02 to 3.54) | 0.04 (0.04 to 0.04) | -3.29% (-3.74 to -2.85) |
| Jamaica | 8.93 (8.09 to 10) | 1.1 (0.99 to 1.23) | 1.4 (1.09 to 1.81) | 0.22 (0.17 to 0.28) | -5.31% (-5.9 to -4.72) |
| Japan | 10.87 (10.48 to 11.22) | 0.04 (0.04 to 0.04) | 1.82 (1.73 to 1.92) | 0.01 (0.01 to 0.01) | -4.56% (-4.72 to -4.4) |
| Jordan | 6.46 (4.73 to 8.91) | 0.43 (0.32 to 0.6) | 5.22 (3.95 to 6.94) | 0.14 (0.1 to 0.18) | -4.04% (-4.34 to -3.73) |
| Kazakhstan | 36.81 (31.46 to 43.56) | 0.77 (0.66 to 0.92) | 3.57 (2.7 to 4.18) | 0.07 (0.06 to 0.09) | -8.61% (-9.21 to -8) |
| Kenya | 18.02 (13.18 to 23.27) | 0.19 (0.14 to 0.24) | 17.37 (12.12 to 25.07) | 0.09 (0.06 to 0.13) | -1.37% (-1.74 to -0.99) |
| Kiribati | 1.16 (0.94 to 1.43) | 4.65 (3.77 to 5.75) | 1.26 (0.93 to 1.71) | 3.22 (2.38 to 4.36) | -1.04% (-1.15 to -0.94) |
| Kuwait | 5 (4.41 to 5.65) | 1.04 (0.92 to 1.17) | 1.26 (1.04 to 1.51) | 0.15 (0.13 to 0.18) | -6.5% (-7.86 to -5.11) |
| Kyrgyzstan | 13.73 (10.84 to 15.31) | 0.93 (0.74 to 1.04) | 3.91 (3.32 to 4.5) | 0.19 (0.16 to 0.22) | -5.3% (-5.89 to -4.7) |
| Lao People's Democratic Republic | 60.4 (38.36 to 87.31) | 3.88 (2.46 to 5.61) | 28.16 (18.86 to 41.88) | 1.3 (0.87 to 1.94) | -3.49% (-3.63 to -3.36) |
| Latvia | 1.15 (0.87 to 1.35) | 0.21 (0.16 to 0.25) | 0.04 (0.03 to 0.04) | 0.01 (0.01 to 0.01) | -9.18% (-9.7 to -8.65) |
| Lebanon | 6.64 (4.61 to 9.65) | 0.7 (0.49 to 1.02) | 2.33 (1.81 to 3.02) | 0.19 (0.14 to 0.24) | -4.07% (-4.18 to -3.96) |
| Lesotho | 5.24 (3.47 to 7.52) | 0.89 (0.59 to 1.27) | 5.52 (3.72 to 8.01) | 0.87 (0.59 to 1.26) | 0.87% (0.51 to 1.23) |
| Liberia | 7.47 (4.94 to 10.54) | 0.83 (0.55 to 1.17) | 6.09 (3.78 to 9.65) | 0.3 (0.19 to 0.48) | -3.32% (-3.74 to -2.91) |
| Libya | 16.51 (10.21 to 25.14) | 0.99 (0.61 to 1.5) | 7.14 (4.38 to 10.3) | 0.43 (0.26 to 0.62) | -2.2% (-2.7 to -1.69) |
| Lithuania | 2.27 (1.98 to 2.67) | 0.28 (0.24 to 0.33) | 0.06 (0.05 to 0.07) | 0.02 (0.01 to 0.02) | -8.68% (-9.27 to -8.09) |
| Luxembourg | 0.03 (0.03 to 0.04) | 0.05 (0.04 to 0.06) | 0.01 (0.01 to 0.01) | 0.01 (0.01 to 0.01) | -5.7% (-6.19 to -5.2) |
| Madagascar | 47.22 (32.27 to 63.22) | 1.03 (0.71 to 1.38) | 62.23 (38.74 to 96.47) | 0.57 (0.36 to 0.89) | -1.7% (-1.81 to -1.59) |
| Malawi | 16.86 (11.8 to 22.42) | 0.46 (0.32 to 0.61) | 13.25 (8.12 to 21.24) | 0.17 (0.1 to 0.27) | -3.27% (-3.52 to -3.02) |
| Malaysia | 66.84 (53.5 to 84.19) | 1.12 (0.9 to 1.41) | 19.54 (14.75 to 25.11) | 0.25 (0.19 to 0.32) | -4.32% (-4.78 to -3.85) |
| Maldives | 1.6 (1.03 to 2.17) | 1.85 (1.19 to 2.51) | 0.23 (0.17 to 0.29) | 0.23 (0.17 to 0.29) | -6.36% (-6.61 to -6.12) |
| Mali | 25 (16.01 to 35.14) | 0.78 (0.5 to 1.09) | 29.28 (19.67 to 40.1) | 0.3 (0.2 to 0.42) | -2.69% (-2.96 to -2.41) |
| Malta | 0.04 (0.03 to 0.04) | 0.04 (0.03 to 0.05) | 0.02 (0.02 to 0.02) | 0.03 (0.02 to 0.04) | -2.31% (-2.84 to -1.79) |
| Marshall Islands | 0.54 (0.42 to 0.7) | 2.81 (2.18 to 3.64) | 0.48 (0.33 to 0.69) | 2.78 (1.89 to 3.97) | -0.35% (-0.75 to 0.05) |
| Mauritania | 4.85 (3.42 to 6.53) | 0.64 (0.45 to 0.86) | 2.57 (1.74 to 3.5) | 0.15 (0.1 to 0.21) | -4.46% (-4.65 to -4.27) |
| Mauritius | 2.73 (2.49 to 3.01) | 0.84 (0.76 to 0.92) | 0.7 (0.6 to 0.81) | 0.3 (0.26 to 0.35) | 0.22% (-1.17 to 1.63) |
| Mexico | 88.1 (84.03 to 92.77) | 0.28 (0.27 to 0.29) | 11.02 (8.2 to 12.61) | 0.03 (0.02 to 0.04) | -6.21% (-6.5 to -5.92) |
| Micronesia (Federated States of) | 1.66 (1.26 to 2.23) | 3.97 (3 to 5.34) | 0.71 (0.49 to 1.03) | 2.23 (1.54 to 3.22) | -1.91% (-1.96 to -1.86) |
| Monaco | 0 (0 to 0) | 0.02 (0.01 to 0.03) | 0 (0 to 0) | 0.01 (0.01 to 0.02) | -2.35% (-2.6 to -2.09) |
| Mongolia | 5.5 (3.28 to 8.47) | 0.69 (0.41 to 1.06) | 2.19 (1.52 to 3.15) | 0.24 (0.16 to 0.34) | -3.3% (-4.12 to -2.48) |
| Montenegro | 0.12 (0.09 to 0.17) | 0.08 (0.05 to 0.1) | 0.04 (0.03 to 0.06) | 0.04 (0.02 to 0.05) | -3.11% (-3.98 to -2.24) |
| Morocco | 114.63 (56.5 to 175.53) | 1.29 (0.63 to 1.97) | 25.45 (15.48 to 41.24) | 0.26 (0.16 to 0.43) | -4.77% (-4.97 to -4.57) |
| Mozambique | 17.97 (11.02 to 25.91) | 0.35 (0.21 to 0.5) | 25.53 (14.54 to 42.12) | 0.2 (0.12 to 0.33) | -1.33% (-1.55 to -1.1) |
| Myanmar | 537.11 (350.26 to 748.37) | 3.82 (2.49 to 5.32) | 172.17 (123.28 to 239.93) | 1.11 (0.79 to 1.54) | -4.4% (-4.66 to -4.14) |
| Namibia | 5.71 (3.93 to 8.1) | 1.07 (0.73 to 1.51) | 3.42 (2.28 to 5.16) | 0.43 (0.29 to 0.65) | -2.78% (-2.88 to -2.68) |
| Nauru | 0.15 (0.11 to 0.21) | 4.16 (3.03 to 5.76) | 0.13 (0.09 to 0.18) | 3.48 (2.39 to 4.74) | -0.64% (-1.19 to -0.09) |
| Nepal | 212.58 (153.34 to 293.68) | 3.01 (2.17 to 4.16) | 124.55 (87.3 to 175.38) | 1.33 (0.93 to 1.88) | -2.48% (-2.57 to -2.39) |
| Netherlands | 0.5 (0.44 to 0.56) | 0.02 (0.02 to 0.02) | 0.17 (0.15 to 0.2) | 0.01 (0.01 to 0.01) | -3.37% (-3.63 to -3.11) |
| New Zealand | 2.45 (2.19 to 2.71) | 0.3 (0.27 to 0.33) | 0.62 (0.54 to 0.71) | 0.06 (0.05 to 0.07) | -5.21% (-5.78 to -4.63) |
| Nicaragua | 4.05 (3.09 to 5.05) | 0.26 (0.19 to 0.32) | 0.7 (0.53 to 0.95) | 0.04 (0.03 to 0.05) | -6.21% (-6.38 to -6.03) |
| Niger | 31.3 (19.86 to 47.27) | 0.99 (0.63 to 1.49) | 33.55 (19.32 to 59.99) | 0.32 (0.18 to 0.57) | -3.63% (-4.03 to -3.23) |
| Nigeria | 291.11 (189.06 to 408.84) | 0.89 (0.58 to 1.25) | 222.4 (144.67 to 307.98) | 0.25 (0.16 to 0.34) | -4.2% (-4.35 to -4.04) |
| Niue | 0.02 (0.01 to 0.02) | 2.38 (1.76 to 3.24) | 0.02 (0.02 to 0.03) | 5.38 (4.16 to 6.88) | -0.05% (-0.95 to 0.86) |
| North Macedonia | 0.76 (0.57 to 1.01) | 0.14 (0.11 to 0.19) | 0.14 (0.09 to 0.21) | 0.04 (0.03 to 0.06) | -4.35% (-4.66 to -4.04) |
| Northern Mariana Islands | 0.08 (0.05 to 0.1) | 0.66 (0.47 to 0.9) | 0.06 (0.05 to 0.08) | 0.52 (0.39 to 0.65) | -0.79% (-2.15 to 0.6) |
| Norway | 0.13 (0.12 to 0.14) | 0.02 (0.01 to 0.02) | 0.02 (0.02 to 0.02) | 0 (0 to 0) | -7.8% (-8.92 to -6.66) |
| Oman | 4.62 (2.89 to 6.69) | 0.68 (0.43 to 0.99) | 1.22 (0.71 to 1.75) | 0.11 (0.07 to 0.16) | -4.3% (-4.76 to -3.84) |
| Pakistan | 1121.03 (803.3 to 1506.24) | 2.64 (1.89 to 3.54) | 1945.21 (1452.3 to 2653.44) | 2.45 (1.83 to 3.34) | -0.27% (-0.51 to -0.02) |
| Palau | 0.14 (0.1 to 0.19) | 2.94 (2.09 to 3.98) | 0.08 (0.06 to 0.11) | 2.33 (1.64 to 3.12) | -0.37% (-0.55 to -0.2) |
| Palestine | 7.53 (4.95 to 11.08) | 0.94 (0.62 to 1.38) | 4.83 (3.75 to 6.16) | 0.27 (0.21 to 0.34) | -3.53% (-4.02 to -3.05) |
| Panama | 2.19 (1.9 to 2.53) | 0.27 (0.24 to 0.32) | 0.54 (0.42 to 0.67) | 0.05 (0.04 to 0.06) | -5.53% (-5.85 to -5.21) |
| Papua New Guinea | 79.45 (35.34 to 123.27) | 5.36 (2.39 to 8.32) | 150.87 (97.3 to 225.18) | 4.47 (2.88 to 6.67) | -0.5% (-0.69 to -0.32) |
| Paraguay | 6 (4.82 to 7.26) | 0.41 (0.33 to 0.5) | 3.76 (2.64 to 5.01) | 0.19 (0.13 to 0.25) | -2.66% (-2.96 to -2.35) |
| Peru | 20.28 (15.58 to 26.04) | 0.26 (0.2 to 0.34) | 6.94 (4.63 to 9.93) | 0.08 (0.05 to 0.11) | -3.89% (-4.14 to -3.63) |
| Philippines | 315.61 (224.55 to 412.37) | 1.38 (0.98 to 1.81) | 218.17 (141.61 to 293.05) | 0.65 (0.42 to 0.87) | -1.66% (-1.97 to -1.36) |
| Poland | 17.83 (17.04 to 18.72) | 0.19 (0.18 to 0.2) | 0.92 (0.79 to 1.02) | 0.02 (0.01 to 0.02) | -7.25% (-7.82 to -6.68) |
| Portugal | 1.87 (1.62 to 2.14) | 0.08 (0.07 to 0.09) | 0.31 (0.26 to 0.37) | 0.02 (0.02 to 0.02) | -5.03% (-5.59 to -4.47) |
| Puerto Rico | 2.1 (1.85 to 2.39) | 0.21 (0.18 to 0.24) | 0.33 (0.26 to 0.41) | 0.06 (0.05 to 0.08) | -5.3% (-5.85 to -4.75) |
| Qatar | 0.61 (0.45 to 0.81) | 0.6 (0.44 to 0.79) | 0.52 (0.36 to 0.7) | 0.13 (0.09 to 0.17) | -4.22% (-4.59 to -3.84) |
| Republic of Korea | 8.47 (6.13 to 11.09) | 0.07 (0.05 to 0.09) | 0.58 (0.42 to 0.78) | 0.01 (0.01 to 0.01) | -6.88% (-7.49 to -6.25) |
| Republic of Moldova | 5.02 (4.38 to 5.83) | 0.44 (0.38 to 0.51) | 0.23 (0.19 to 0.28) | 0.04 (0.03 to 0.05) | -7.88% (-8.35 to -7.41) |
| Romania | 11.33 (9.82 to 13) | 0.2 (0.17 to 0.23) | 1.2 (0.91 to 1.48) | 0.04 (0.03 to 0.05) | -5.42% (-5.86 to -4.98) |
| Russian Federation | 92.25 (90.52 to 94.28) | 0.27 (0.27 to 0.28) | 4.28 (3.92 to 4.55) | 0.02 (0.01 to 0.02) | -9.26% (-9.87 to -8.66) |
| Rwanda | 21.9 (13.13 to 33.15) | 0.78 (0.47 to 1.19) | 6.52 (3.9 to 10.15) | 0.14 (0.08 to 0.22) | -6.32% (-6.69 to -5.94) |
| Saint Kitts and Nevis | 0.06 (0.05 to 0.07) | 0.44 (0.39 to 0.5) | 0.02 (0.01 to 0.02) | 0.15 (0.12 to 0.18) | -3.86% (-4.22 to -3.5) |
| Saint Lucia | 0.29 (0.25 to 0.33) | 0.59 (0.52 to 0.67) | 0.08 (0.06 to 0.1) | 0.25 (0.19 to 0.31) | -3.35% (-3.71 to -2.99) |
| Saint Vincent and the Grenadines | 0.43 (0.38 to 0.49) | 1.06 (0.92 to 1.19) | 0.12 (0.1 to 0.14) | 0.44 (0.36 to 0.53) | -2.89% (-3.26 to -2.52) |
| Samoa | 1.37 (0.96 to 1.85) | 2.07 (1.45 to 2.79) | 1.01 (0.73 to 1.4) | 1.41 (1.02 to 1.95) | -1.09% (-1.21 to -0.98) |
| San Marino | 0 (0 to 0) | 0.04 (0.03 to 0.05) | 0 (0 to 0) | 0.01 (0.01 to 0.02) | -3.33% (-3.56 to -3.11) |
| Sao Tome and Principe | 0.41 (0.28 to 0.59) | 0.83 (0.58 to 1.2) | 0.22 (0.12 to 0.36) | 0.28 (0.16 to 0.47) | -3.4% (-3.83 to -2.96) |
| Saudi Arabia | 35.89 (21.81 to 55.63) | 0.62 (0.38 to 0.96) | 6.29 (4.21 to 8.84) | 0.08 (0.05 to 0.11) | -6.47% (-6.58 to -6.36) |
| Senegal | 24.79 (16.53 to 36.27) | 0.83 (0.55 to 1.22) | 13.23 (8.64 to 20.78) | 0.23 (0.15 to 0.36) | -3.87% (-4.16 to -3.57) |
| Serbia | 1.95 (1.45 to 2.58) | 0.09 (0.07 to 0.12) | 0.17 (0.12 to 0.24) | 0.01 (0.01 to 0.02) | -6.8% (-7.18 to -6.42) |
| Seychelles | 0.1 (0.08 to 0.13) | 0.42 (0.33 to 0.55) | 0.01 (0 to 0.01) | 0.03 (0.02 to 0.04) | -2.6% (-4.12 to -1.06) |
| Sierra Leone | 12.71 (8.36 to 17.82) | 0.89 (0.58 to 1.24) | 11.81 (7.78 to 17.48) | 0.37 (0.24 to 0.54) | -2.25% (-2.47 to -2.02) |
| Singapore | 0.47 (0.42 to 0.54) | 0.07 (0.06 to 0.08) | 0.13 (0.11 to 0.15) | 0.02 (0.01 to 0.02) | -4.71% (-5.05 to -4.36) |
| Slovakia | 1.26 (0.95 to 1.63) | 0.09 (0.07 to 0.12) | 0.18 (0.12 to 0.25) | 0.02 (0.01 to 0.03) | -4.6% (-4.8 to -4.41) |
| Slovenia | 0.58 (0.5 to 0.67) | 0.13 (0.11 to 0.15) | 0.03 (0.02 to 0.04) | 0.01 (0.01 to 0.01) | -9.25% (-9.72 to -8.78) |
| Solomon Islands | 3.16 (1.78 to 4.94) | 2.34 (1.32 to 3.66) | 3.96 (2.7 to 5.58) | 1.68 (1.15 to 2.37) | -1.05% (-1.16 to -0.94) |
| Somalia | 18.89 (12.1 to 27.45) | 0.59 (0.38 to 0.86) | 24.79 (14.53 to 43.13) | 0.29 (0.17 to 0.5) | -2.67% (-2.94 to -2.41) |
| South Africa | 93.25 (79.88 to 107.27) | 0.73 (0.63 to 0.84) | 46.29 (38.38 to 54.63) | 0.31 (0.26 to 0.37) | -3.63% (-4.38 to -2.89) |
| South Sudan | 11.46 (6.13 to 16.94) | 0.51 (0.27 to 0.75) | 15.25 (9.03 to 23.34) | 0.39 (0.23 to 0.59) | -1.26% (-1.81 to -0.71) |
| Spain | 9.64 (8.67 to 10.84) | 0.11 (0.1 to 0.12) | 2.27 (2 to 2.6) | 0.03 (0.03 to 0.04) | -3.98% (-4.22 to -3.73) |
| Sri Lanka | 56.77 (42.89 to 71.8) | 1.04 (0.78 to 1.31) | 12.56 (8.41 to 18.12) | 0.24 (0.16 to 0.34) | -5% (-5.4 to -4.6) |
| Sudan | 167.8 (91.15 to 264.99) | 2.22 (1.2 to 3.5) | 102.9 (57.91 to 172.93) | 0.65 (0.37 to 1.1) | -3.59% (-3.75 to -3.42) |
| Suriname | 1.03 (0.57 to 1.34) | 0.81 (0.45 to 1.06) | 0.47 (0.33 to 0.64) | 0.32 (0.23 to 0.44) | -2.93% (-3.42 to -2.44) |
| Sweden | 0.32 (0.28 to 0.36) | 0.02 (0.02 to 0.02) | 0.04 (0.04 to 0.05) | 0 (0 to 0) | -5.46% (-5.95 to -4.97) |
| Switzerland | 0.68 (0.58 to 0.78) | 0.06 (0.05 to 0.07) | 0.18 (0.15 to 0.2) | 0.01 (0.01 to 0.02) | -4.87% (-5.03 to -4.71) |
| Syrian Arab Republic | 129 (89.63 to 177.68) | 2.48 (1.73 to 3.42) | 20.6 (13.36 to 29.64) | 0.46 (0.3 to 0.67) | -6.2% (-6.81 to -5.59) |
| Taiwan (Province of China) | 4.95 (4.38 to 5.62) | 0.09 (0.08 to 0.1) | 0.45 (0.38 to 0.53) | 0.01 (0.01 to 0.02) | -5.88% (-6.54 to -5.21) |
| Tajikistan | 10.83 (7.33 to 14.92) | 0.56 (0.38 to 0.77) | 9.16 (6.54 to 12.42) | 0.29 (0.21 to 0.4) | -3.02% (-3.49 to -2.55) |
| Thailand | 114.12 (71.76 to 170.82) | 0.65 (0.41 to 0.97) | 20.39 (14.53 to 27.89) | 0.19 (0.13 to 0.26) | -4.36% (-4.64 to -4.07) |
| Timor-Leste | 5.99 (3.92 to 8.14) | 2.25 (1.47 to 3.05) | 5.83 (3.76 to 8.37) | 1.17 (0.75 to 1.67) | -2.17% (-2.71 to -1.63) |
| Togo | 10.75 (7.3 to 14.95) | 0.73 (0.5 to 1.02) | 7.24 (4.54 to 11.5) | 0.24 (0.15 to 0.38) | -3.35% (-3.49 to -3.21) |
| Tokelau | 0.02 (0.01 to 0.02) | 2.85 (2.1 to 3.71) | 0.02 (0.02 to 0.03) | 5.29 (3.94 to 7.17) | -1.07% (-2.2 to 0.09) |
| Tonga | 0.33 (0.25 to 0.42) | 0.87 (0.67 to 1.11) | 0.22 (0.15 to 0.29) | 0.61 (0.42 to 0.84) | -1.02% (-1.35 to -0.69) |
| Trinidad and Tobago | 3.09 (2.75 to 3.47) | 0.8 (0.71 to 0.9) | 0.8 (0.61 to 1.03) | 0.28 (0.21 to 0.36) | -3.18% (-3.44 to -2.91) |
| Tunisia | 19.7 (12.08 to 27.82) | 0.67 (0.41 to 0.95) | 3.79 (2.2 to 6.07) | 0.14 (0.08 to 0.23) | -4.53% (-4.84 to -4.23) |
| Türkiye | 95.78 (61.88 to 150.73) | 0.49 (0.31 to 0.76) | 23.89 (17.75 to 31.26) | 0.12 (0.09 to 0.16) | -3.69% (-4 to -3.37) |
| Turkmenistan | 16.24 (13.47 to 18.72) | 1.25 (1.04 to 1.44) | 5.01 (3.68 to 6.46) | 0.35 (0.26 to 0.45) | -5.59% (-6.19 to -4.98) |
| Tuvalu | 0.13 (0.1 to 0.18) | 4.84 (3.77 to 6.38) | 0.08 (0.06 to 0.1) | 2.1 (1.53 to 2.86) | -2.46% (-2.56 to -2.35) |
| Uganda | 24.24 (14.5 to 39.85) | 0.36 (0.22 to 0.59) | 25.78 (16.69 to 40.95) | 0.15 (0.09 to 0.23) | -3.75% (-4.11 to -3.38) |
| Ukraine | 6.18 (4.82 to 7.31) | 0.05 (0.04 to 0.06) | 1.05 (0.81 to 1.35) | 0.02 (0.01 to 0.02) | -4.41% (-5.31 to -3.51) |
| United Arab Emirates | 5.32 (3.62 to 7.62) | 1.11 (0.76 to 1.59) | 2.67 (1.9 to 3.72) | 0.21 (0.15 to 0.29) | -4.7% (-5.3 to -4.1) |
| United Kingdom | 4.41 (4.31 to 4.53) | 0.04 (0.04 to 0.04) | 0.63 (0.57 to 0.68) | 0.01 (0 to 0.01) | -6.44% (-6.68 to -6.21) |
| United Republic of Tanzania | 38.39 (26.08 to 54.21) | 0.38 (0.26 to 0.54) | 41.86 (25.47 to 65.68) | 0.19 (0.12 to 0.3) | -1.99% (-2.12 to -1.86) |
| United States of America | 27 (26.3 to 27.75) | 0.05 (0.05 to 0.05) | 8.38 (7.72 to 8.88) | 0.01 (0.01 to 0.01) | -4.51% (-5.1 to -3.91) |
| United States Virgin Islands | 0.15 (0.11 to 0.19) | 0.48 (0.35 to 0.61) | 0.02 (0.01 to 0.03) | 0.11 (0.06 to 0.19) | -4.15% (-4.46 to -3.83) |
| Uruguay | 0.51 (0.45 to 0.58) | 0.06 (0.06 to 0.07) | 0.11 (0.09 to 0.12) | 0.01 (0.01 to 0.02) | -4.94% (-5.28 to -4.6) |
| Uzbekistan | 71.99 (59.51 to 86.03) | 0.99 (0.81 to 1.18) | 39.75 (31.23 to 48.56) | 0.45 (0.36 to 0.55) | -2.09% (-2.6 to -1.58) |
| Vanuatu | 2.12 (1.48 to 2.81) | 3.76 (2.62 to 4.98) | 3.16 (2.26 to 4.28) | 3 (2.14 to 4.07) | -0.92% (-1.16 to -0.69) |
| Venezuela (Bolivarian Republic of) | 16.24 (14.57 to 18.13) | 0.25 (0.22 to 0.28) | 2.99 (2.13 to 3.89) | 0.05 (0.03 to 0.06) | -5.88% (-6.26 to -5.5) |
| Viet Nam | 144.09 (98.39 to 201.23) | 0.59 (0.41 to 0.83) | 55.83 (37.1 to 77.47) | 0.24 (0.16 to 0.33) | -2.49% (-2.68 to -2.29) |
| Yemen | 84.06 (42.82 to 153.25) | 1.49 (0.76 to 2.72) | 63.66 (34.35 to 108.55) | 0.5 (0.27 to 0.85) | -3.75% (-3.89 to -3.6) |
| Zambia | 12.92 (8.44 to 19.48) | 0.4 (0.26 to 0.61) | 10.58 (6.01 to 17.34) | 0.14 (0.08 to 0.23) | -3.63% (-4.03 to -3.23) |
| Zimbabwe | 71.57 (49.28 to 94.8) | 1.68 (1.16 to 2.23) | 171.41 (126.29 to 230.28) | 2.97 (2.19 to 3.99) | 2.93% (2.33 to 3.52) |
